# Paired mutation calling and spatial transcriptomics identify cellular neighborhoods associated with the neoplastic outcome of mouse colitis

**DOI:** 10.1038/s41588-026-02673-0

**Published:** 2026-07-28

**Authors:** Elisa B. Moutin, Linus L. H. Chang, Giada Giavara, Shenay Mehmed, Mathilde Colombé, Carla Boquetale, Kate Marks, Filipe C. Lourenço, Nefeli Skoufou-Papoutsaki, Richard Kemp, Philippe Gascard, Thea D. Tlsty, David S. Tourigny, Douglas J. Winton

**Affiliations:** 1 CRUK Cambridge Institute, University of Cambridge, Cambridge, UK; 2 School of Mathematics, University of Birmingham, Birmingham, UK; 3 Leeds Institute of Medical Research, University of Leeds, Leeds, UK; 4 Department of Pathology, University of California, San Francisco, San Francisco, CA USA

**Keywords:** Colon cancer, DNA sequencing, RNA sequencing, Inflammatory bowel disease

## Abstract

In the progression from inflammatory bowel disease to associated cancer, the clonal mutational landscape shifts from selection of mutations in inflammatory genes to selection for cancer-driver mutations. How prevalence and expansion of either type of mutant clones could be impacted by the cellular environments in which they arise and how this affects the neoplastic outcome of colitis remains unknown. Here we combine in vivo lineage tracing, in silico modeling, mutational profiling and spatial transcriptomics in a mouse model of colitis-associated tumorigenesis to capture clone fates associated with chronic inflammation. We identify epithelial- and immune-enriched neighborhoods and propose a model in which establishment of a reparative tissue environment facilitates tumor initiation by promoting the selection and expansion of pro-oncogenic clones, reducing the span of inflammation-resistant neighborhoods containing nononcogenic clones.

## Abstract (web summary)

This study uses a mouse model of inflammatory bowel disease to explore how mutations and cellular context combine to drive the transition to malignancy.

## Main

Inflammatory bowel disease (IBD) affects 7 million people worldwide and is associated with many co-morbidities, including the development of colitis-associated cancer (CAC)^1^. Previous studies have shown that, during the progression of IBD, mutations affecting genes implicated in mounting an inflammatory response are under positive selection in the colonic epithelium^2–4^. In these studies, clones bearing such mutations exhibited an increased capacity to survive and expand in colonic tissue, which was linked with resistance to inflammatory insult. Mechanisms thought to underlie this resistance are diverse: mutations in the IL17 and NF-κB pathways act directly by dampening inflammatory signaling, while others such as *Arid1a* inactivation act through chromatin remodeling to alter the transcriptional response to inflammatory cues^5^. Extensive characterization of the mutational landscape of CAC has revealed a reduced prevalence of inflammation-resistance mutations in tumors arising in the context of IBD^6–10^, hinting at a protective role against cancer. This could explain why most patients are only diagnosed with colorectal neoplasia more than a decade after their initial IBD diagnosis^11,12^. How selection of inflammation-resistant clones is influenced by the environment in which they arise and how this affects the balance between IBD pathogenesis and neoplastic transformation has not yet been defined, in part due to a lack of model systems.

Using lineage tracing to profile clone dynamics in the well-characterized *Muc2*^KO^ model of colitis^13–15^, we observe discrete areas of the tissue in which clonal expansion is favored. In silico modeling of clone dynamics and inference of effective crypt fission rate defines the tissue repair process. The expansion of mutated clones is studied using targeted amplicon sequencing after chemical mutagen treatment of *Muc2*^KO^ mice. Importantly, clones bearing mutations found in human IBD and associated cancer are identified. Combination with spatial transcriptomics data originating from the same samples allows pairwise identification and phenotypic characterization of cellular neighborhoods enriched for clones with pro-oncogenic or anti-inflammatory mutations, respectively, positively or negatively associated with the neoplastic outcome of chronic inflammation.

## Results

### Lineage tracing captures increased clonal expansion in colitis

**Extended Data Fig. 1:**
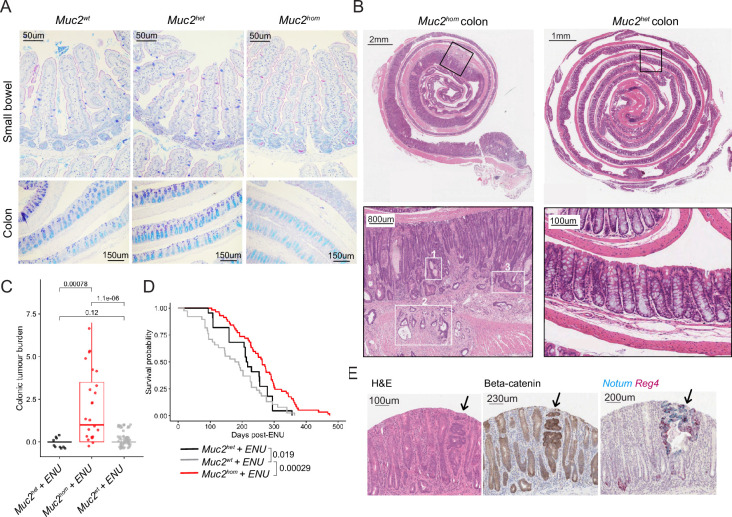
Modelling colitis and associated-cancer. **A**) Images showing AB/Pas staining on *Muc2*^wt^, *Muc2*^het^ and *Muc2*^hom^ small bowel and colon. **B**) Swiss-rolled *Muc2*^hom^ and *Muc2*^het^ colon stained with haematoxylin and eosin (H&E). Panel on the bottom zooms in representative areas and reveals signs of pathology in *Muc2*^hom^ colon (1. abscess, 2. epithelial foci invading muscle layer, 3. loss of crypt polarity). **C**) Number of colonic nuclear ß-catenin tumours found post-ENU treatment in a cohort of n=23 *Muc2*^hom^, n=11 *Muc2*^het^ and n=57 wild-type mice (p value for Wilcoxon signed rank-test showed on graph). **D**) Kaplan-Meier survival curve for wild-type, *Muc2*^hom^ and *Muc2*^het^ mice post-ENU treatment (n=57, 38 and 22 respectively, p values for pairwise log-rank with BH adjustment shown). **E**) Images showing H&E staining, beta-catenin IHC and Notum/Reg4 RNAscope on an on-edge piece of tissue from the colon of a *Muc2*^hom^ mouse treated with ENU. Arrow points to a tumour.

Depletion of the intestinal mucus barrier due to constitutive knockout (KO) of both alleles of the *Muc2* gene leads to chronic inflammation restricted to the mouse colon^16^ (Extended Data Fig. 1a,b). As previously described by us and others, this model recapitulates the pathology seen in patients suffering from IBD^14,15^, including the development of epithelial dysplasia and emergence of invasive foci with age (Extended Data Fig. 1b).

**Fig. 1:**
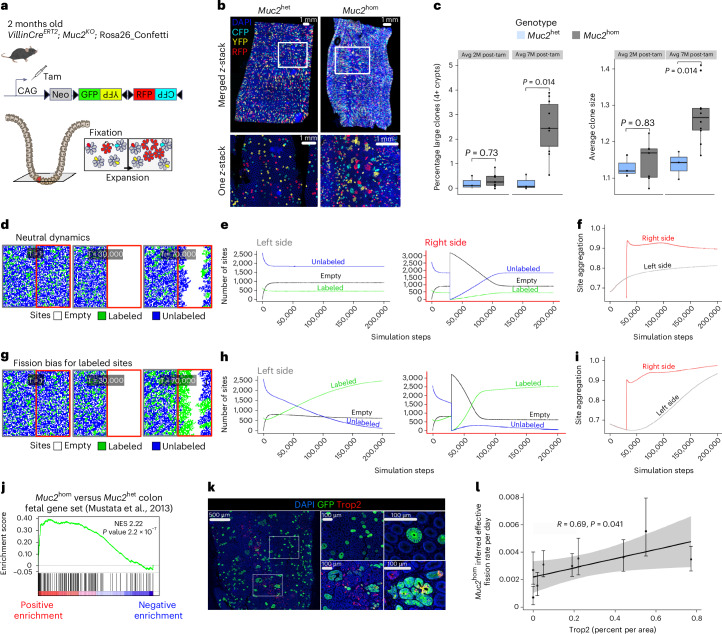
Impact of colitis-associated tissue repair on clonal fates. **a**, In vivo genetic lineage tracing using Villincre^ERT^; *Muc2*^KO^; Rosa26^fl^Confetti reporter mice. Tamoxifen treatment leads to Cre-mediated excision of the Neomycin cassette, resulting in heritable expression of either GFP, YFP, RFP or CFP in intestinal epithelial cells. Clones can achieve high intestinal epithelial burden by two processes: (1) fixation, relating to the capacity of stem cells to compete with others at the crypt base to achieve monoclonality; (2) expansion, relating to the capacity of a clone to fission into more crypts. **b**, Three-dimensional images (top) and one *z*-stack pictures (bottom) showing increased clone sizes in the *Muc2*^hom^ compared with *Muc2*^het^ mouse colon. **c**, Box plot showing percentage of large clones (left) and average clone size (right) in animal groups for which average survival is either 2 months post tamoxifen (*Muc2*^het^, *n* = 3; *Muc2*^hom^, *n* = 7) or 7 months post tamoxifen (*Muc2*^het^, *n* = 3; *Muc2*^hom^, *n* = 10). For each box plot, the center line represents the median and the box bounds show the 25th and 75th percentiles. Whiskers extend up to 1.5× IQR from the box. Outliers are plotted as individual points. *P* values for two-sided Wilcoxon tests on the median are showed on the plot. 2M, 2 months; 7M, 7 months. **d**, Representative grid at simulation steps corresponding to beginning of simulation (*T* = 1), introduction of damage (*T* = 30,000) and ongoing repair (*T* = 70,000). **e**, Line plots showing average number of labeled, unlabeled or empty sites per simulation step in each side of the lattice (no damage (left) and damage (right) at *T* = 30,000) for 1,000 simulations in neutral dynamics conditions. **f**, Line plot showing aggregation of sites for each simulation step in both sides of the lattice, averaged over 1,000 simulations. **g**, Representative snapshots as in **d** with fission bias in labeled sites (0.95 versus 0.5 unlabeled). **h**, Line plots as in **d**, for fission bias condition. **i**, Line plot showing aggregation of sites as in **f** for fission bias condition. **j**, GSEA on bulk RNA sequencing material from *Muc2*^hom^ and *Muc2*^het^ mouse colon using a fetal gene set^57^ (*n* = 3 mice per condition; statistical significance was tested with a permutation-based test with 1,000 permutations, with the false discovery rate adjusted for multiple comparisons). NES, normalized enrichment score. **k**, Immunofluorescence pictures showing confetti clones stained with GFP in a Trop2-positive and Trop2-negative area of the *Muc2*^hom^ mouse colon. **l**, Dot plot showing linear correlation between percentage of area expressing Trop2 and inferred effective fission rate. Points represent median and error bars indicate the 2.5th and 97.5th quantiles of *N* = 3,000 samples capturing the 95% credible interval of the posterior distribution for each effective fission rate (*n* = 3 *Muc2*^hom^ mice, three regions per mouse colon, coefficient and *P* value for two-sided Pearson correlation on plot).

Acute colonic inflammation was previously shown to induce massive clonal expansion associated with tissue repair^17^. To observe whether similar processes are at play during disease progression in the *Muc2*^KO^ model of colitis, Cre-mediated activation of the Confetti lineage-tracing cassette was performed by tamoxifen treatment of mice containing inter-crossed *R26*^*Confetti*^ and *VillinCre*^*ERt2*^ alleles^18^ on a *Muc2*^KO^ background (Fig. 1a). Large clones containing multiple crypts sharing a common Confetti color and that are known to arise through crypt fission^19^ could be observed in discrete areas of the *Muc2*^hom^ mouse colon (Fig. 1b). Quantification of clone sizes at 7 months post tamoxifen injection revealed a significant increase in average clone size and number of large clones in the colonic epithelium of *Muc2*^hom^ mice compared with control *Muc2*^het^ mice, which was not apparent at 2 months post tamoxifen injection (Fig. 1c). This indicates a clear relationship between duration of inflammation and extent of clonal expansion.

### Increased crypt fission drives reparative neutral clonal expansion

An in silico model was developed to test the hypothesis that repair following tissue damage enhances the extent of clone expansion seen under normal homeostatic conditions. A discrete two-dimensional lattice with a hexagonal arrangement of sites and periodic boundary conditions representing a cylindrical geometry in the tangential direction was randomly populated with 80% unlabeled and 20% labeled crypts and evolved forward in time in a sequence of steps allowing the transition of lattice sites between three possible states: either empty or occupied by an unlabeled or labeled crypt (Fig. 1d). In this model, transitions between states at each lattice site are governed by the probability of clone fixation, crypt replication by fission and crypt fusion, based on the identities of the six neighboring sites^20,21^ (Methods and Supplementary Fig. 1). To replicate the effects of acute damage, the entire right side of the lattice was emptied and sites from the undamaged side allowed to repopulate the damaged side of the lattice.

Before damage, the number of empty sites is maintained at a plateau due to a balancing of fission and fusion events involving labeled and unlabeled crypts, considered here as homeostasis (Fig. 1e). To capture the extent of clonal expansion seen in vivo, we derived a metric quantifying aggregation of either labeled or unlabeled sites (Methods). Increased aggregation was seen in the right (damaged) side of the lattice postdamage and persists at an elevated level compared with the left (undamaged) side even after a return to homeostasis (Fig. 1e,f), illustrating how the process of repair following damage drives a neutral expansion of clones in proximity to injury.

### Tissue repair favors fission-biased clones

Mutations in cancer-driving genes such as *Kras* have been shown to confer a large fission bias to mutant clones^22^. To study the potential impact of such biases on expansion in tissue repair conditions, in silico simulations were adapted to give a fission advantage to labeled crypts (fission probability of 0.95 for labeled crypts versus 0.5 for unlabeled) (Fig. 1g). This led to faster repair of the damaged right side (*T* = 80,000 simulation steps with fission advantage versus 115,315 without) (Fig. 1g,h), with fission-biased crypts consistently outcompeting wild-type crypts in both right and left sides over time. This was linked to a stable increase in aggregation of sites on both sides of the lattice that was, importantly, more pronounced in the damaged right side (Fig. 1i). This highlights how tissue repair conditions can accelerate the expansion and accumulation of fission-biased clones in damaged tissue.

### Fetal gene expression marks repair-associated crypt fission

To identify markers associating with epithelial repair, transcriptional profiles of *Muc2*^hom^ mouse colons were derived. Previous observations in a chemically induced colitis model using dextran sulfate sodium (DSS) have identified a fetal gene signature characteristic of the repairing epithelium^23^. Enrichment for this signature was confirmed in the *Muc2*^hom^ model by gene set enrichment analysis (GSEA) following transcriptomic profiling of *Muc2*^hom^ and *Muc2*^het^ colon samples from ten-month-old animals (Methods) (Fig. 1j). Immunodetection revealed presence of large clones in areas where the fetal marker Trop2 (*Tacstd2*) is expressed (Fig. 1k). Estimates of effective crypt fission rates derived from manual clone size measurements (Methods) showed a significant positive correlation with the extent of Trop2 expression within the areas profiled (Fig. 1l). This result establishes repairing areas of the mouse colonic epithelium as environments where clonal expansion through crypt fission is favored. Notably, restriction of this response to damaged areas reflects the localized pathology observed in patients with ulcerative colitis.

### Identifying cellular neighborhoods in CAC

To model the tissue environment driving accumulation and selection of mutations leading to the development of CAC, *Muc2*^hom^ mice were treated with the chemical mutagen *N*-ethyl-*N*-nitrosourea (ENU). This alkylating agent has been shown by us and others to trigger tumor initiation in genetically primed contexts^24–26^. Following ENU treatment, tumors arise in the colon of *Muc2*^hom^ but not *Muc2*^het^ mice (Extended Data Fig. 1c), resulting in reduced survival in the former (Extended Data Fig. 1d). These tumors arise as flat dysplastic lesions, phenocopying CAC (Extended Data Fig. 1e). Notably, tumors express *Notum* and *Reg4*, which are documented markers of Wnt pathway activation and CAC, respectively^27,28^.

**Fig. 2:**
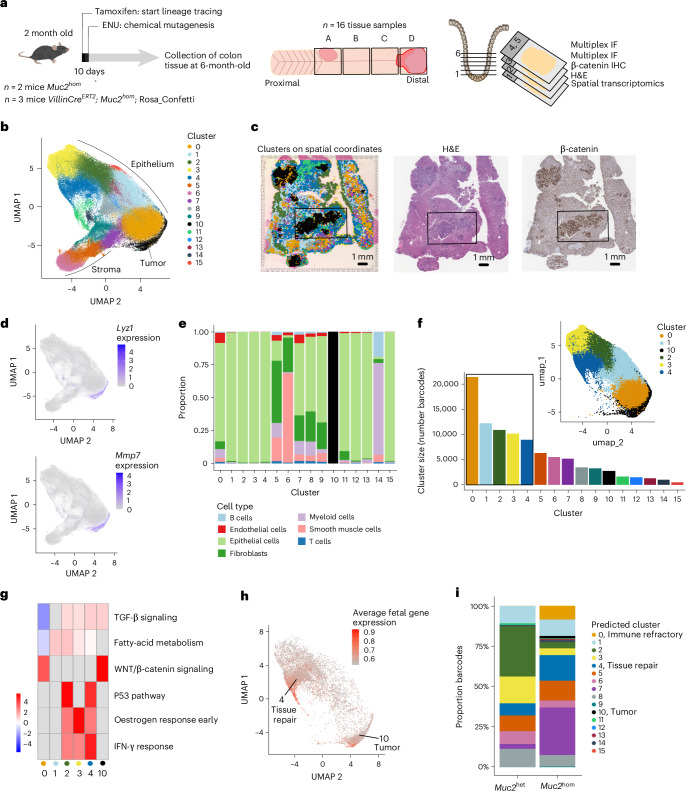
Spatial transcriptomics detects cellular neighborhoods in colitis and associated neoplasia. **a**, *Muc2*^hom^ mice received 1 IP injection of ENU at 8-weeks-old; 4 months post-ENU, proximal and distal pieces of colon tissue were collected and fixed. Serial sections were cut starting at the base of the crypt and going up and used for: (1) spatial transcriptomic profiling with 10x Visium, (2) H&E staining, (3) immunohistochemistry (IHC) for β-catenin, followed by multiplex immunofluorescence (*n* = 5 mice, 16 tissue pieces). IF, immunofluorescence. **b**, UMAP of clusters detected with Visium after integration of all slides from *Muc2*^hom^ + ENU colon tissue. **c**, Clusters plotted on spatial coordinate, H&E and β-catenin IHC on the same 11-mm^2^ piece of *Muc2*^hom^ + ENU mouse colon (see Extended Data Fig. 4a for cluster coordinates for all slides). **d**, UMAPs showing distribution of expression of *Lyz1* and *Mmp7*, markers of the tumor cluster. **e**, Filled bar plots showing proportion of assigned cell types in each cluster, using the DWLS deconvolution tool (Methods). **f**, Bar plot depicting the number of Visium barcodes per cluster. Inlet shows the UMAP restricted to the five main epithelial clusters (0–4) and tumor cluster (10). **g**, Results of GSVA showing pathway enrichment for each of the main epithelial clusters, as well as the tumor cluster (top enriched pathway for each cluster showed on plot, where the gray means no significant change). **h**, Plot showing average expression of the fetal gene set^57^ on UMAP space. **i**, Filled bar plots showing the proportion of barcoded spots assigned to each cluster in *n* = 3 *Muc2*^het^ and *n* = 3 *Muc2*^hom^ colon samples.

**Extended Data Fig. 2:**
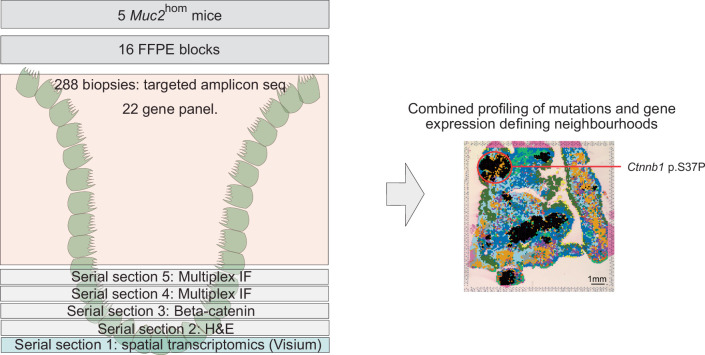
Approach for joint mutation calling and profiling of cellular neighbourhoods. 6 FFPE colon tissue serial sections were obtained from 5 *Muc2*^hom^ mice treated with ENU. Sections were cut from the base of the crypts towards the lumen and used for 1. spatial transcriptomic profiling with the 10x Visium FFPE protocol, 2. Haematoxylin and Eosin (H&E) staining, 3. Immunohistochemistry (IHC) for ß-catenin, followed by multiplex immunofluorescence (IF) in 4. E-cadherin, GFP, RFP, Trop2 and 5. DAPI, E-cadherin, Arid1a (n=5 mice, 16 tissue pieces). Tissue directly above the serial sections used for spatial transcriptomic and multiplex IFs was sampled in series of 2mm diameter biopsies, to which was applied multiplexed targeted amplicon sequencing covering 22 genes previously identified in human Inflammatory Bowel Disease and/or colitis associated cancer.

**Extended Data Fig. 3:**
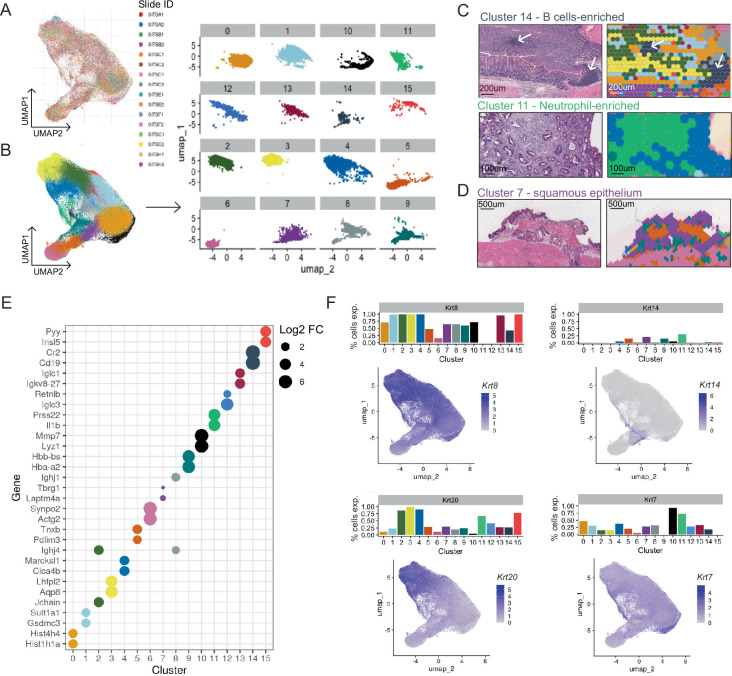
Spatial transcriptomics detects cellular neighbourhoods in colitis and associated neoplasia. **A**) UMAP of barcodes coloured by slide ID, showing overlap in samples after Harmony integration. **B**) Clusters represented in UMAP space with and without overlay. **C**) Clusters on spatial coordinates (right) and representative H&E image (left), arrows pointing to isolated lymphoid follicles marked by cluster 14. **D**) Clusters on spatial coordinates (top) and representative H&E image (bottom) in an area containing muscle and squamous epithelium. **E**) Dot plot showing top 2 markers for each cluster (see Methods), with size of dots representing log2 fold change in the relevant cluster compared to all others. **F**) Bar plots showing percentage expression of different keratins in the cells assigned to each cluster (top) and UMAPs (below) showing distribution of expression of the same keratins (*Krt8*: general epithelial marker, *Krt14*: squamous epithelium, *Krt7*: epithelial carcinogenesis, *Krt20*: healthy intestinal epithelium).

**Extended Data Fig. 4:**
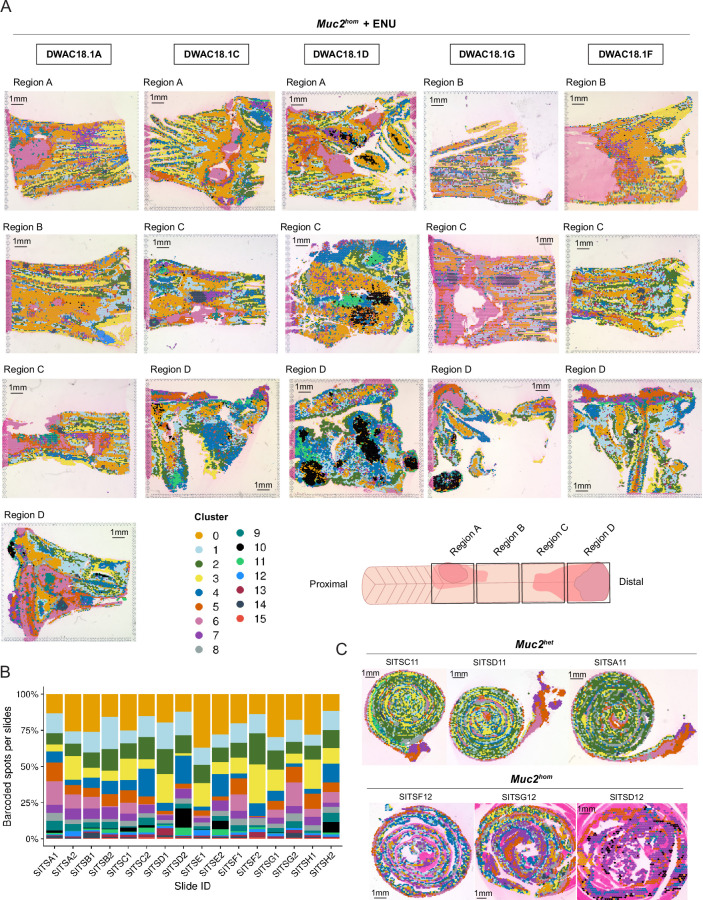
Spatial representation of clusters. **A**) Clusters plotted onto spatial coordinates for colon tissue from *Muc2*^hom^ mice treated with ENU. Mouse ID is indicated at the top of each column, and region indicated at the top of each picture for *en face* tissue. A drawing shows the location of each region along the mouse colon. **B**) Filled bar plot showing the distribution of clusters for each slide from the *Muc2*^hom^+ENU cohort. **C**) Clusters plotted onto spatial coordinates for colon tissue from *Muc2*^het^ (top) and *Muc2*^hom^ mice (bottom).

To determine how expansion of mutated clones associates with the environment in which they arise, we designed an experimental approach allowing spatial transcriptomics and mutation detection to be overlaid, for spatial molecular characterization of cellular neighborhoods arising in colitis (Extended Data Fig. 2). Gene expression data were obtained from crypt-sized barcoded spots (10x Genomics Visium) covering sixteen tissue samples from proximal and distal regions of the *Muc2*^hom^ mouse colon showing the most advanced chronic inflammation and associated neoplasia pathologies^15^ (Fig. 2a). Transcriptomic data from all samples were integrated into a single dataset before unsupervised clustering and uniform manifold approximation and projection (UMAP) reduction (Methods; Extended Data Fig. 3a). Sixteen phenotypic clusters were identified (Fig. 2b and Extended Data Fig. 3b). Mapping barcoded spots within each cluster to their spatial coordinates revealed the organization of cellular neighborhoods in each tissue sample (Extended Data Fig. 4a,b).

### Cell-type composition of cellular neighborhoods

To characterize the cell-type composition of neighborhoods, we first examined the genes that mark each cluster. Several discrete phenotypic clusters expressed immune cell markers, such as the B cell markers *Iglc1* in cluster 13 and *Cd19* in cluster 14^29^ or the myeloid cells marker *Il1b*^30^ in cluster 11, indicating that they represent immune-enriched neighborhoods (Extended Data Fig. 3b,c,e). Clusters 5 and 6 show abundant expression of myopodin (*Synpo2*) and actin (*Actg2*), indicating that these neighborhoods are enriched for muscle cells (Extended Data Fig. 3b,d,e). Clusters 12 and 15 are marked by the goblet cell marker resistin-like β (*Retlnb*) and the enteroendocrine cell marker peptide YY (*Pyy*), respectively (Extended Data Fig. 3e). Of note, the presence of specialized intestinal cell populations is likely to be affected by tissue orientation.

Similarly, expression patterns of keratins were used to identify neighborhoods enriched with intestinal epithelial cells^31,32^ (Extended Data Fig. 3f). Widespread expression of keratin 8 indicates good epithelial representation across the dataset, with a gradient of keratin 20 suggesting higher colonocyte differentiation, indicative of healthy epithelium, in clusters 2, 3 and 4 compared with other clusters. Keratin 14 is high in cluster 7 and overlays squamous epithelium (Extended Data Fig. 3d,f). Cluster 10 shows high keratin 7 and low keratin 20 expression. Notably, keratin 7 is not expressed in healthy colonic epithelium but has been found elevated in patients with IBD^33^, whereas low expression of keratin 20 indicates poor colonocyte differentiation. These observations are aligned with pathological assessment using hematoxylin and eosin (H&E), as well as β-catenin immunohistochemistry showing elevated nuclear β-catenin overlaying cluster 10, identifying it unambiguously as a cluster containing tumor cells (Fig. 2c). Markers distinguishing the tumor cluster 10 from the major epithelial neighborhoods include *Mmp7* and *Lyz1*, two genes normally associated with Wnt-producing Paneth cells in small intestinal crypts that are commonly ectopically expressed in intestinal tumors^34–37^ (Fig. 2d and Extended Data Fig. 3e).

To further refine predictions of the main cell types contributing to each nontumor phenotypic cluster, deconvolution of cell-type compositions was performed based on the dampened weighted least squares (DWLS) method^38^ using cell-type signatures generated from a single-cell RNA-sequencing experiment with mouse colon^39^ (Methods). This analysis confirmed the assignment of the major phenotypic clusters 0–4 as enriched with epithelial cells, while the full set of 16 clusters display varying proportions of endothelial, fibroblast, smooth muscle and immune cell types (Fig. 2e). Importantly, this analysis revealed which of the minor clusters represent immune cell islands (that is, *Cd19*^+^ cluster 14) as opposed to epithelium enriched in immune cells (that is, *Iglc1*^+^ cluster 13 and *Il1b*^+^ cluster 11).

### Biological pathways separating epithelial and tumor neighborhoods

Epithelial-rich clusters 0–4 contain the largest percentages of barcoded spots (Fig. 2f and Extended Data Fig. 4a,b), indicating that these cellular neighborhoods cover most of the tissue. To identify biological pathways active in these major epithelial-rich clusters and the tumor cluster, we performed gene set variation analysis (GSVA)^40^ using the MSigDB hallmark gene sets (Fig. 2g and Supplementary Table 2). Tumor cluster 10 showed enrichment for Wnt signaling pathway activation, consistent with elevated nuclear β-catenin staining (Fig. 2c). The five nontumor, major epithelial-rich neighborhoods display an assortment of pathway activities that reflects varied responses to inflammation. Cluster 4 shows enrichment for several inflammatory pathways including interferon gamma (IFN-γ), as compared with the other clusters. This cluster also shows the highest enrichment for the fetal epithelial repair signature, which includes Trop2 (Fig. 2h), establishing it as a neighborhood associated with epithelial repair, probably in response to inflammatory cues. At the other extreme, cluster 0, which contains the largest proportion of infiltrating immune cells out of the five major epithelial-rich clusters (Fig. 2e), is characterized by negative enrichment of inflammation-related pathways such as TGF-β and interferon-α signaling relative to other clusters (Supplementary Table 2). This suggests that cells in this neighborhood are less responsive to immune activation, that is, immune refractory. Cluster 0 is also enriched for Wnt signaling alongside the tumor cluster, indicative of their relative transcriptional similarity seen in the UMAP (Fig. 2f). By comparison, pathways active in the remaining epithelial-rich clusters 1, 2 and 3 indicate normal intestinal epithelium function (for example, fatty acid metabolism) and some enrichment for IFN-γ response as seen in the tissue repair cluster 4. To validate this interpretation, additional spatial gene expression data was obtained from colonic tissues from three *Muc2*^het^ and three *Muc2*^hom^ mice that were not treated with ENU. This confirmed that epithelial-rich barcoded spots from control *Muc2*^het^ mice mapped predominantly to clusters 1, 2 and 3, suggesting these clusters represent neighborhoods that are not impacted by inflammation. On the other hand, *Muc2*^hom^ tissue contained a larger proportion of barcoded spots mapping to the immune-refractory clusters 0 and repair cluster 4 (Fig. 2i and Extended Data Fig. 4b).

### Evolution of cellular neighborhoods with neoplasia

**Fig. 3:**
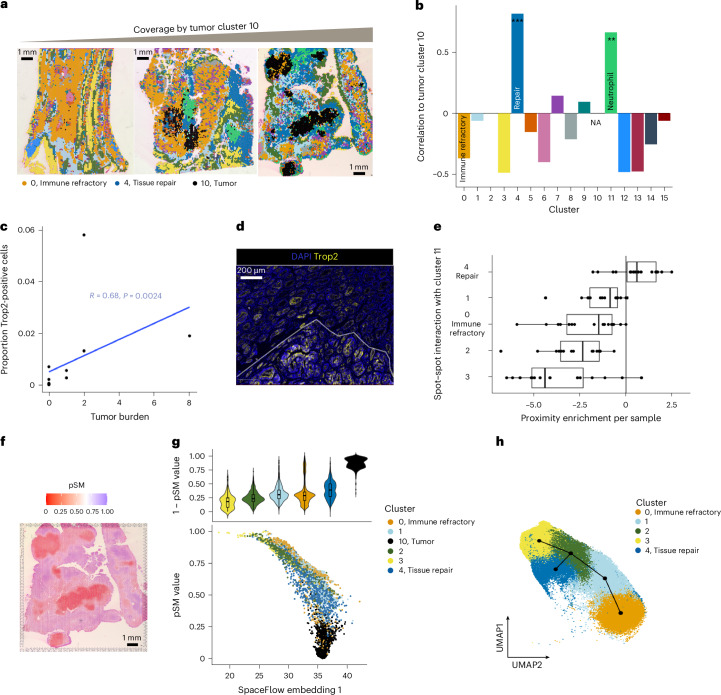
Evolution of cellular neighborhoods in inflammation-associated carcinogenesis. **a**, Clusters mapped onto spatial coordinates showing the relative coverage by each of the clusters as the coverage by the tumor cluster 10 increases. **b**, Bar plot showing correlation based on symmetric balance in slide coverage by the tumor cluster (10) and the coverage by each of the other clusters. A two-sided Pearson correlation test was used with no adjustment for multiple comparison, **P < 0.01, ***P < 0.001. NA, non applicable. **c**, Dot plot showing the relationship between tumor burden and number of Trop2-positive cells (*n* = 5 mice, 14 tissue samples). Correlation was assessed with a two-sided Kendall rank test, with coefficient and *P* value showed on plot. **d**, Representative immunofluorescence image relating to **c**, showing a tumor arising in a Trop2-positive epithelial field. **e**, Box plots showing proximity enrichment calculated with Giotto between each of the main epithelial cluster and the neutrophil cluster 11 in each *Muc2*^hom^ + ENU slide profiled with Visium (*n* = 5 *Muc2*^hom^ + ENU mice, 16 slides). For each box plot, the center line represents the median and the box bounds show the 25th and 75th percentiles. Whiskers extend up to 1.5× IQR from the box. Outliers are plotted as individual points. **f**, pSM values plotted on spatial coordinates for sample SITSD2. **g**, Top: box plots summarizing pSM values for each epithelial cluster in sample SITSD2 (box plot statistics derived from cells mapped to each cluster in one *Muc2*^hom^ + ENU slide, box plot elements as in **e**). Bottom: pSM values for each barcoded spot in sample SITSD2 mapping to the main epithelial clusters and the tumor cluster, plotted with regards to the Spaceflow embedding 1. **h**, UMAP showing the main epithelial clusters, overlayed with lineage topology precited by SlingShot (black lines).

Different cellular neighborhoods may contract or expand with the progression of disease, being displaced by or displacing others. To identify trends between the proportion of tissue covered by barcoded spots (tissue coverage) mapping to a given cellular neighborhood and tissue coverage by the tumor cluster, neighborhoods were correlated based on symmetric balances^41^ (Fig. 3a,b; Methods). The strongest positive symmetric balance correlations in tissue coverage with the tumor cluster were observed for epithelial repair cluster 4 (R = 0.81, *P* = 1.21 × 10^−4^) and cluster 11 (*R* = 0.66, *P* = 5.22 × 10^−3^) (Fig. 3b), which is enriched in several genes marking neutrophils (*Il1b*, *Cd11b*, *Cd18*, *Eln*, *S100a9* and *Mmp9*) (Supplementary Table 1). These positive correlations between tumor, epithelial repair and neutrophil-enriched neighborhoods may be indicative of an association between the establishment of a tissue repair environment and tumor formation. Accordingly, *Tacstd2* (Trop2) is upregulated in cluster 11 (log_2_ fold change 3.52, adjusted *P* < 1.5 × 10^−300^; Supplementary Table 1). Using immunofluorescence for the marker of tissue repair Trop2, we could find a positive correlation between the proportion of Trop2-positive cells in a tissue sample and the number of tumors detected by nuclear β-catenin staining (*R* = 0.68, *P* = 0.0024) (Fig. 3c,d). Moreover, repair cluster 4 was found to be the only major nontumor neighborhood to display a positive spatial association with the neutrophil-enriched cluster 11 (Fig. 3e). Together, these findings suggest that tumor initiation is accompanied by the establishment of epithelial repair and neutrophil-enriched neighborhoods, highlighting a spatial interplay between tissue repair and neoplasia.

To understand whether dynamical processes such as cell differentiation or disease progression could underlie the relationship between the tumor cluster and the major epithelial-rich neighborhoods, we used SpaceFlow^42^, a spatially regularized deep graph model. This approach, previously used to identify pre-neoplastic lesions in human breast spatial transcriptomics data^42^, infers a pseudo-spatiotemporal ordering of transcriptomic states across tissue sections (Fig. 3f). In the sample with the highest tumor burden, pseudo-spatiotemporal values suggested an ordering beginning with cluster 3, probably representing the healthiest epithelial-rich neighborhood, and progressing through clusters 1 and 2, followed by the immune-refractory cluster 0 and finally the repair cluster 4 (Fig. 3g). To further assess lineage relationships between the major epithelial-rich neighborhoods, we applied Slingshot^43^, a computational tool used in single-cell analyses to infer development trajectories and order cell states along differentiation paths. Here, Slingshot identified two branching epithelial trajectories originating at cluster 3 and diverging at cluster 2. Integrating these results with the SpaceFlow-derived pseudo-spatiotemporal ordering suggests that epithelial-rich neighborhoods follow a common progression from the healthy state (cluster 3) that bifurcates into two distinct paths: one leading to the repair lineage (cluster 4) and the other to the immune-refractory lineage (cluster 0) (Fig. 3h).

The positive symmetric balance correlations between repair cluster 4, neutrophil-enriched cluster 11 and the tumor cluster, implies that the progression along the repair lineage identified by Slingshot is highly correlated with increased tumor burden. By contrast, while not achieving statistical significance, negative symmetric balance correlations observed for cluster 0 with the tumor cluster (Fig. 3b) are consistent with progression along the immune-refractory lineage, being negatively associated with the evolution of neoplastic disease.

### Exploring cellular neighborhoods in alternative tumor models

**Extended Data Fig. 5:**
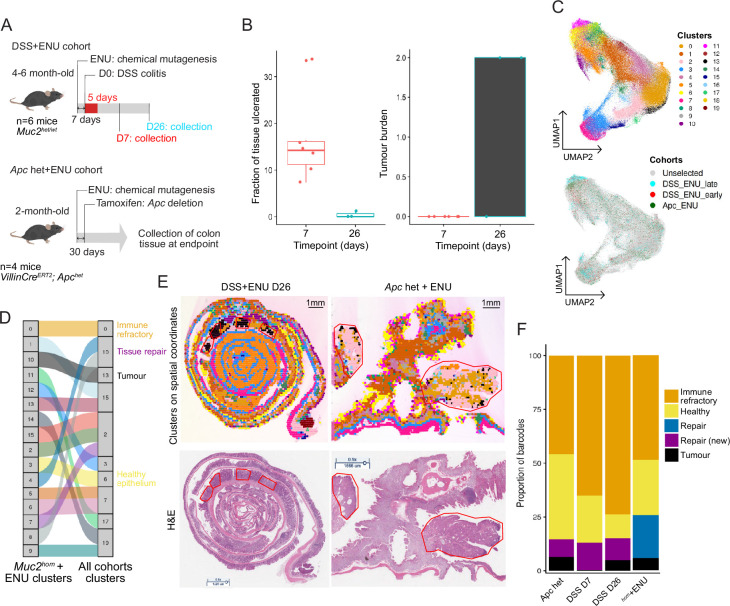
Cellular neighbourhoods in alternative tumour models. **A**) Top: n=6 VillinCre^ERT2^; *Muc2*^het^/^wt^; Rosa_Confetti mice received 1 intraperitoneal injection of ENU at 4-months-old. 7 days later, the mice were provided 2% DSS in drinking water for a total of 5 days. n=3 mice were collected 7 days after the start of the DSS treatment (active inflammation) and n=3 mice were collected 26 days after the start of the DSS treatment (repair/tumourigenesis). Bottom: n=4 VillinCre^ERT2^; *Apc*^het^ mice received 1 IP injection of ENU at 2-months-old. 30 days later, the mice were treated with Tamoxifen to delete one copy of the *Apc* gene. The colon tissue was collected as mice reached a humane endpoint. Swiss-rolled colon sections (DSS+ENU cohort) or *en face* colon sections (*Apc*+ENU cohort) were used for spatial transcriptomics (10X Visium) and H&E staining. **B**) Boxplot showing the fraction of colon tissue ulcerated (left) and bar plot the number of nuclear ß-catenin tumours detected (right) at each timepoint. **C**) UMAP showing cluster distribution after integration of all cohorts (top) and distribution of the DSS+ENU and *Apc*+ENU samples (bottom). **D**) Alluvial plot showing correspondence between clusters from the *Muc2*^hom^+ENU cohort and clusters obtained after integration of all cohorts (*Muc2*^hom^+ENU, *Muc2*^hom^, *Muc2*^het^, DSS+ENU, *Apc*+ENU), with the width of the flows reflecting the correlation in gene expression between the matched clusters (see methods). **E**) Clusters on spatial coordinates for one representative sample of the DSS+ENU cohort at day 26 post-DSS (left) and the *Apc*+ENU cohort (right). Tumours are circled in black. Bottom pictures are the relevant H&E pictures. **F**) Filled bar plot showing the proportion of barcodes assigned to several clusters previously identified in the *Muc2*^hom^+ENU cohort.

To assess whether the cellular neighborhoods identified in *Muc2*^hom^ mice treated with ENU are found in other colorectal tumorigenesis contexts, we analyzed two additional cohorts. First, six *Muc2*^het^ or *Muc2*^wt^ mice were treated with ENU followed by DSS, mimicking classical chemical-driven inflammation and tumorigenesis (Extended Data Fig. 5a). Three mice were collected per time point. At 7 days post-DSS treatment, the tissue was largely ulcerated, while at 26 days post-DSS treatment, the tissue had been repaired, and nuclear β-catenin tumors were detected in two out of three mice (Extended Data Fig. 5b). Second, to examine the scenario in which tumorigenesis precedes inflammation (sporadic tumorigenesis), we collected colon tissue from four VillinCre^ERT^; *Apc*^+/−^ mice which were treated with ENU 30 days before the deletion of one copy of the *Apc* gene (*Apc*^*het*^, Extended Data Fig. 5a). All mice in this cohort developed several large colonic tumors that were morphologically akin to sporadic colon cancer.

Spatial gene expression data for both cohorts were integrated with the *Muc2*^hom^ + ENU cohort into a single dataset before unsupervised clustering and UMAP reduction (Extended Data Fig. 5c), identifying 19 phenotypic clusters. To assess the correspondence between these clusters and cellular neighborhoods identified in *Muc2*^hom^ + ENU mice, we calculated correlations between averaged gene expression profiles (Methods). Based on a correlation coefficient cutoff of 0.8, 10 out of 19 clusters could be confidently mapped to clusters observed in the *Muc2*^hom^ + ENU cohort (Extended Data Fig. 5d). The proportion of barcoded spots corresponding to healthy epithelium decreases from day 7 to day 26 in the DSS + ENU cohort, while the proportion of barcoded spots mapping to the immune-refractory neighborhood increases, accompanied by the emergence of barcoded spots mapping to the tumor cluster (Extended Data Fig. 5f). Both immune-refractory (0) and the closest correspondent to the tissue repair cluster (10) could be found in close proximity to tumors (Extended Data Fig. 2e).

The proportion of barcoded spots mapping to healthy epithelium was higher in the sporadic colon cancer model (*Apc*^*het*^ + ENU) compared either DSS + ENU or *Muc2*^hom^ + ENU cohorts (Extended Data Fig. 3f). In this cohort, barcoded spots mapping to the original tumor cluster identified in *Muc2*^hom^ + ENU mice did not largely overlay tumors detected by H&E staining (Extended Data Fig. 3e). This was instead the case for cluster 2, representing another cellular neighborhood associated with sporadic tumors (Extended Data Fig. 3c,e). Top markers for this cluster include *Col1/2*, *Dcn*, *Vim* and *Timp3*, previously linked with cancer-associated fibroblasts present in the tumor stroma and promoting colon cancer metastasis^44,45^ (Supplementary Table 3). Together, these data suggest shared mechanisms of tumorigenesis between chemical inflammation-driven neoplasia and spontaneous colitis-associated neoplasia, including the emergence of tissue repair neighborhoods adjacent to tumors. By contrast, sporadic *Apc*-driven tumors exhibit a distinct stromal composition, characterized by the presence of a CAF-rich neighborhood not observed in *Muc2*^hom^ + ENU lesions, highlighting context-specific stromal adaptations that depend on the initiating insult.

### Detection of mutations associated with IBD and CAC

**Fig. 4:**
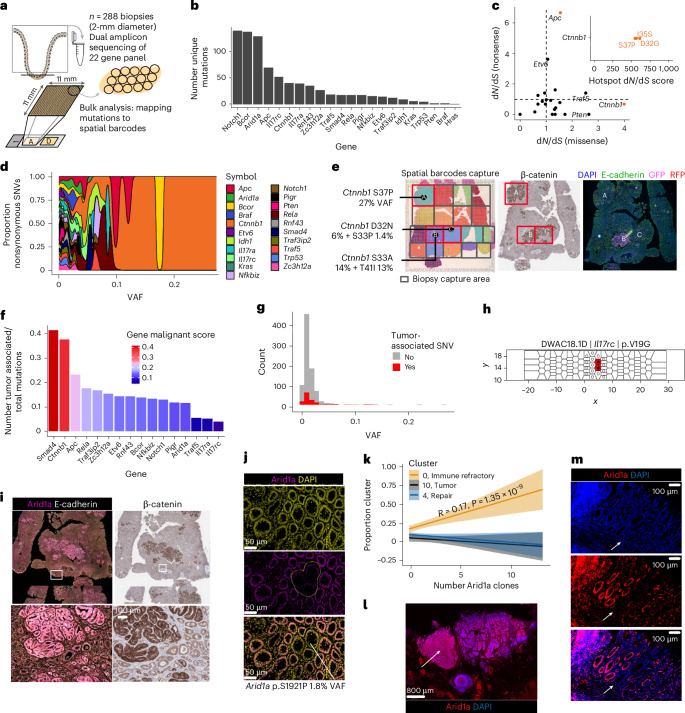
Selection of malignant and nonmalignant clones in epithelial neighborhoods. **a**, Tissue directly above the serial sections used for transcriptomic and multiplex IF profiling was sampled in series of 2-mm-diameter biopsies, to which was applied multiplexed targeted sequencing covering 22 genes previously identified in human IBD and/or CAC. **b**, Bar plot showing the number of mutations detected for genes covered in the panel (genes showed only if mutations were detected). **c**, Dot plot showing selection for nonsense mutations (*y* axis) and missense mutations (*x* axis). Red dots denote significant positive selection (d*N*/d*S* analysis^46^; Methods). Inset shows results from d*N*/d*S* hotspot analysis. **d**, Density plot showing proportion of nonsynonymous and stop-gained SNVs at different VAF. **e**, Grid and colors depict biopsies used for targeted amplicon sequencing (three biopsies are highlighted in red and SNVs in *Ctnnb1* annotated with VAF). The same biopsies are represented on a β-catenin immunohistochemistry, and the corresponding immunofluorescence image shows lineage tracing with GFP detecting one clone corresponding to tumor B (right). **f**, Bar plot showing number of tumor-associated/total nonsynonymous mutations per gene, representing the malignant potential of each gene. **g**, Histogram showing VAF distribution for tumor-associated SNVs (red) or nontumor-associated SNVs (gray). **h**, Tessellation mask for a piece of tissue showing expansion of an *Il17rc* mutated nonmalignant clone. **i**, Immunofluorescence images stained for Arid1a (left) and associated β-catenin staining (right) showing upregulated Arid1a protein expression in tumors. **j**, Immunofluorescence images stained for Arid1a, with DAPI nuclear counterstain, showing loss of Arid1a staining corresponding to an *Arid1a* missense mutation detected at 1.8% VAF. **k**, Line plot showing the linear relationship between number of Arid1a-depleted clones detected with immunostaining in a biopsy and proportion of each cluster (the line is the global linear model fit, the shading is the standard error; model estimate and *P* value shown when *P* < 0.05. Statistical significance was assessed using a two-sided Wald test on global linear model coefficients, with no additional adjustment made for multiple comparison). **l**, Immunofluorescent pictures showing Arid1a upregulation in a colitis-associated tumor in a 76-year-old male patient with CAC. **m**, Immunofluorescence pictures showing an Arid1a-depleted clone in the inflamed mucosa of the same patient.

**Extended Data Fig. 6:**
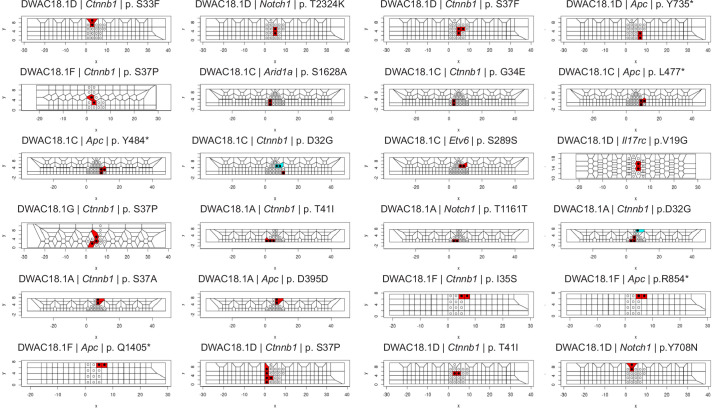
Voronoi Tessellation of tissue sections for clone calling. Plots representing each piece of tissue where the same mutation was detected in at least 2 adjacent biopsies. Black dots represent biopsies taken for the relevant piece of tissue, filled dots represent the mutation called in that biopsy, and colour represent clones (considered distinct if distance > 2mm). Repeated patterns on either side show the Tessellation that was applied in order to detect adjacent cores at tissue borders.

To determine whether the accumulation and expansion of mutant clones found in human IBD and CAC is associated with specific epithelial neighborhoods, we profiled the mutational landscape in the colonic epithelium of the *Muc2*^hom^ + ENU cohort. Colon tissue directly overlaying the sections used for spatial transcriptomics and immunofluorescence was sub-sampled in a series of 2-mm-diameter biopsies (Fig. 4a). Replicate targeted amplicon sequencing for 22 genes commonly found mutated in IBD and/or CAC (Supplementary Table 6) was applied to each of the 288 biopsies. A tessellation algorithm using spatial coordinates was used to identify adjacent biopsies with the same mutation and parsimoniously assign these to the same clonal event (Extended Data Fig. 6; Methods). Single-nucleotide variants (SNVs) were detected in all genes covered by the amplicon panel, including known CAC drivers *Trp53*, *Kras* and *Smad4*^6^ but also several genes associated with the IL-17 pathway (*Il17rc*, *Il17ra* and *Pigr*) and chromatin remodelers *Arid1a* and *Bcor* commonly found mutated in the nondysplastic colonic epithelium of patients with IBD^2–4^ (Fig. 4b and Supplementary Table 5).

Analysis of d*N*/d*S*^46^ ratios identified *Ctnnb1* and *Apc* as genes under significant positive selection for missense and nonsense mutations, respectively (Fig. 4c; Methods). This selection is also reflected in the variant allele frequencies (VAFs) reached by *Ctnnb1* mutations, which dominate the SNV landscape above 10% VAF (Fig. 4d). Such mutations could be mapped to tumors with elevated nuclear β-catenin staining, with VAFs reaching up to 27% in a single biopsy (Fig. 4e), consistent with Wnt signaling activation as a driver of tumor growth.

**Extended Data Fig. 7:**
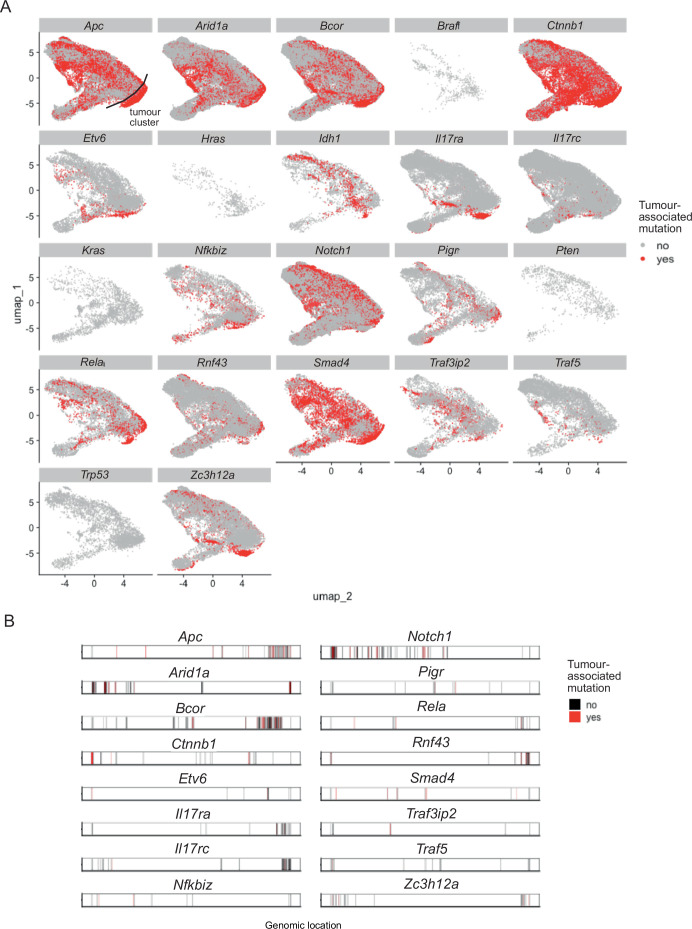
Identifying tumour associated mutations. **A**) Distribution of tumour-associated and non-tumour-associated SNVs in each gene plotted on UMAP space (tumour cluster is circled in black in top left UMAP, size of dot indicates VAF). **B**) Tile plots showing distribution of tumour-associated and non-tumour-associated SNVs along each gene length (features genes with >10 mutations detected).

A malignancy score contextualizing each gene by its tumor association was defined by dividing the number of nonsynonymous SNVs detected for a given gene in a biopsy containing at least five barcoded spots from the tumor cluster (tumor-associated mutations) by the total number of nonsynonymous SNVs detected in that gene overall (Fig. 4f, Extended Data Fig. 7a and Supplementary Table 6). Correspondingly, *Smad4* and *Ctnnb1* have the highest malignancy scores (41% and 37%, respectively). This is consistent with d*N*/d*S* identifying hotspots at amino-acid positions 32-41 in the *Ctnnb1* gene (Fig. 4c and Extended Data Fig. 7b) and previous reports showing *Smad4* mutations are associated with poor-prognosis, recurrence and resistance to treatment in patients with colorectal cancer^47,48^ and found in 13% of CACs^6^.

### IBD mutations accumulate outside the tumor neighborhood

Although known CRC and CAC driver mutations were found enriched within tumor biopsies and corresponded to the highest VAFs (Fig. 4g), genes with low malignancy scores, included *Pigr*, *Arid1a*, *Nfkbiz*, *Il17ra* and *Il17rc* (12%, 12%, 13%, 6% and 4%, respectively) (Fig. 4f), which all display strong positive selection in nondysplastic colonic epithelium from patients with IBD^2–4^. Such genes could be found mutated in clones spanning several biopsies (Fig. 4h), showing a capacity for either selectively neutral or positive expansions mirroring human IBD.

Inactivation of *Arid1a* has been found to be under the strongest positive selection in three separate studies of IBD epithelium^2–4^. To examine whether preferential selection for mutations in genes with low malignant scores could explain the negative association of the immune-refractory lineage with neoplastic disease, as previously revealed by pseudo-spatiotemporal ordering and trajectory inference, immunostaining was used to detect variation in Arid1a protein expression in the *Muc2*^hom^ + ENU model of CAC. Arid1a upregulation was detected in tumors (Fig. 4i), suggesting that nonmutational stabilization of Arid1a could be required for initiation or growth of β-catenin-driven tumors. Conversely, the same antibody allowed the detection of loss or downregulated Arid1a expression in mutant clones located outside of tumors (Fig. 4j). Indeed, the proportion of Arid1a-deficient clones detected via immunostaining displays a significant positive correlation with the proportion of the biopsy covered by the immune-refractory cluster 0, while being negatively correlated with the tumor cluster 10 and associated repair cluster 4 (global linear model intercept of 0.17, *P* = 1.35 × 10^−9^) (Fig. 4k). These results support an interpretation where clones carrying *Arid1a* inactivating mutations are selected for in environments where responses to inflammatory cues are dampened and possibly outcompete neoplastic founder clones in such neighborhoods.

Consistent with the discovery that Arid1a-depleted clones accumulate in immune-refractory neighborhoods, away from tumors, clonal Arid1a depletion was identified in epithelium spatially distinct from tumors using immunostaining in colonic tissue from patients with CAC (Fig. 4l,m). In addition, Arid1a upregulation was observed in tumors of patients with CAC, as was seen in the *Muc2*^hom^ + ENU model. These findings underscore the translational relevance of this study.

## Discussion

Mutant clones undergoing positive selection in IBD may impact pathogenesis and development of CAC. Detailed cataloging of mutations and assessment of their location and function in patients is complicated by the paucity of mutational events, limited sampling methods and confounding effects of treatment. Here, a mouse model of colitis was leveraged to profile inflammation and cancer associated mutant clones, and the selective environments in which they arise, which together shape the conditions favoring different phenotypic outcomes, including neoplasia. Enrichment for *Mmp7* and other extracellular matrix remodeling genes in tumor neighborhoods (Supplementary Table 1), a signature of nascency^49^, supports the claim that this model captures early stages of tumor initiation, rarely accessible in human samples.

Tissue repair and the associated extracellular matrix remodeling is commonly viewed as cancer promoting^50,51^. Recently, epigenetic memory of the repair state has been shown to accelerate tumor growth following oncogenic mutation^52^.

Here, we outlined the process by which epithelial repair promotes clonal expansion by crypt fission and showed that mutations conferring higher fission capacity expand even more within the repairing epithelium. Using spatial transcriptomics, we linked the expansion of a cellular neighborhood associated with epithelial repair to increasing tumor burden and neutrophil-rich neighborhoods. Neutrophil infiltration is known to contribute to oxidative stress and genomic instability^53^. It is therefore plausible that recruitment of neutrophils promotes the malignant transformation of cells participating in epithelial repair.

In contrast to tissue repair, we demonstrated that the establishment of an immune-refractory environment is negatively associated with tumor burden. This immune-refractory cellular neighborhood is positively associated with accumulation of mutant clones previously found to undergo positive selection in the nondysplastic epithelium of patients with IBD, including those harboring *Arid1a* inactivation or mutations in the IL-17 and NF-κB pathways^2–4^. Expansion of these nontumor-associated mutated clones reported both in the present study and previous work suggests they may contribute to the maintenance of such immune-refractory environments. This aligns with recently published evidence that *Arid1a*-deficient crypt patches create immune-exclusion environments in the normal human colonic epithelium^5^.

Despite evidence from previous studies indicating that the extent of these mutant clone expansions may increase the severity of ulcerative colitis^2–4^, our work implies that establishment of an immune-refractory environment may simultaneously act to prevent expansion of tumor-associated mutations, thereby delaying malignant transformation in diseased patients. This is analogous to the expansion of *Notch1* mutated clones in the esophagus, which could outcompete cancer-driver clones, limiting their ability for malignant transformation^54,55^.

Our findings highlight the importance of strategies for long-term management of IBD that promote epithelial repair while limiting malignant transformation. This may be particularly relevant considering the incidence of IBD in children and trend toward younger age of onset^56^. Therapeutic approaches modulating the clonal composition of the chronically inflamed epithelium therefore hold promise for reducing cancer risk in patients with IBD.

## Methods

### Mice

Mice were of C57BL/6 background. The *Muc2*^KO^ line used was described by Velcich et al.^16^. Mice containing inter-crossed *R26*^*Confetti*^ and *VillinCre*^*ERt2*^ alleles^18^ were bred to *Muc2*^KO^ mice to generate *VillinCreERt2; Muc2*^KO^*; R26*^*Confetti*^ mice. To model sporadic colon carcinogenesis, the intestinal epithelium-specific inducible Cre *VillinCre*^*ERt2*^ line was crossed with *Apc*^*fl/*+^(ref. ^58^). All genotyping was outsourced to Transnetyx.

### Animal husbandry

Animal care and procedures were performed at the Cancer Research UK Cambridge Institute Biological Resource Unit according to the UK Home Office under the authority of a Home Office project licence (PD5F099BE) approved by the Animal Welfare and Ethical Review Body at the CRUK Cambridge Institute, University of Cambridge. Mice were housed under controlled conditions (temperature (21 ± 2 °C), humidity (55% ± 10%), 12 h light–dark cycle) in a specific-pathogen-free facility (tested according to the recommendations for health monitoring by the Federation of European Laboratory Animal Science Associations). Animals had unrestricted access to food and water. None of the mice had been involved in any procedure before the study. To trigger acute inflammation, 2% DSS was provided in drinking water for 5 days. To trigger colon carcinogenesis, 200 mg kg^−1^ ENU was injected intraperitoneally. Mice which showed clinical signs of tumor burden (anemia, hunching and loss of body condition) before experimental endpoints were culled and removed from the study.

### Human tissue

Colon tissue samples were obtained from patients with IBD and/or CAC at St James University Hospital Leeds under full local research ethical committee approval (12/LO/1217 approved by London–Bloomsbury Research Ethics Committee) according to the Health Research Authority institution. Patient consent was obtained at the time of surgery, and they did not receive compensation. Colectomy specimens were fixed in 10% neutral buffered formalin and embedded en face in paraffin (FFPE) blocks.

### Three-dimensional imaging and clone counting

The whole colon of *VillinCreERt2; Muc2*^KO^*; R26*^*Confetti*^ mice was dissected, flushed with cold PBS, cut longitudinally and whole-mounted. Following fixation in 4% paraformaldehyde overnight at 4 °C, the tissue was washed in PBS and selected colon segments were excised. Optical clearing was performed using the CUBIC protocol^59^. In brief, excised segments were incubated with CUBIC-1a solution (10% urea, 5% *N*,*N*,*N*′,*N*′-tetrakis(2-hydroxypropyl) ethyl-enediamine, 10% Triton X-100 and 25 mM NaCl in distilled water) at 37 °C for 7–10 days with alternate day solution changes. 4′,6-diamidino-2-phenylindole (DAPI) was used for nuclear counterstaining at a dilution of 1:1,000. The cleared tissue was then washed in PBS for 24 h. Additional clearing and refractive index matching were performed with Rapiclear 1.52 (152002, SunJin Labs) for 24 h. Samples were mounted in a 0.25-mm i-Spacer (Sunjin Labs) for confocal imaging on a Leica SP5 TCS confocal microscope (LAS software v2.8.0, Leica Biosystems) with a 10× objective, 1.4–1.7 optical zoom and 8–12 μm *z*-steps throughout the whole thickness of the tissue. Clone counting was performed using the cell counter tool in the ImageJ software.

### Immunohistochemistry

Mouse colons were opened longitudinally and cut in approximately 15-mm^2^ sections placed in cassettes and fixed overnight at 4 °C in 4% paraformaldehyde. H&E staining was performed using an automated ST5020 Multistainer (Leica Biosystems). Staining for β-catenin was performed on Leica’s automated Bond-III platform in conjunction with the Polymer Refine Detection System (DS9800, Leica Biosystems). In brief, epitope retrieval was performed using Leica Epitope Retrieval Solution 1 (AR9961, Leica Biosystems) at 100 °C. Blocking was performed with Protein Block Buffer (X090930-2, Dako). Following incubation with primary antibody against β-catenin (0.25 μg ml^−1^, mouse, 610154, BD Biosciences), sections were incubated with secondary antibody (1:1,500, rabbit anti-mouse IgG1, ab125913, Abcam) before development and mounting. For β-catenin staining, an additional mouse-on-mouse blocking step was performed.

### Immunofluorescence

Heat-mediated epitope retrieval was performed on rehydrated 3-μm-thick paraffin sections in 10 mM sodium citrate (pH 6.0). The sections were then incubated in blocking solution (10% donkey serum and 0.05% Tween-20 in PBS) at room temperature for 30 min. Primary antibodies against RFP (1:100, rabbit, R10367, Thermo Fisher), GFP (1:100, chicken, ab13970, Abcam), E-cadherin (1:200, mouse, 610182, BD Biosciences), Trop2 (1:100, goat, AF1122, R&D systems) and Arid1a (1:100, rabbit, 12354S, Cell Signaling) were diluted in blocking solution, in which sections were then incubated in the dark overnight at 4 °C. Sections were washed and incubated with fluorophore-conjugated secondary antibodies (donkey anti-rabbit A31572, donkey anti-goat A21447 and/or donkey anti-chicken 703-6-5-155, Thermo Fisher and/or donkey anti-mouse ab150109, Abcam) diluted 1:200 in 0.05% Tween-20 in PBS for 45 min at room temperature. DAPI (1:1,000) was used for nuclear counterstaining. After washing, the stained sections were mounted using ProLong Gold Antifade Mountant (P36930, Thermo Fisher).

### RNAscope

Simultaneous detection of *Notum* and *Reg4* was performed on paraffin embedded sections using Advanced Cell Diagnostics (ACD) RNAscope 2.5 LS Duplex Reagent Kit (322440), RNAscope 2.5 LS Probe- Mm- Notum C1 (428988) and RNAscope 2.5 LS Probe-Mm-Reg4-C2 (409608). The 3-µm-thick sections were baked for 1 h at 60 °C before loading onto a Bond RX instrument (Leica Biosystems). Slides were deparaffinized and rehydrated on board before pre-treatments using Epitope Retrieval Solution 2 (AR9640, Leica Biosystems) at 95 °C for 15 min and ACD Enzyme from the Duplex Reagent kit at 40 °C for 15 min. Probe hybridization and signal amplification were performed according to manufacturer’s instructions. Fast red detection of C2 was performed on the Bond Rx using the Bond Polymer Refine Red Detection Kit (DS9390, Leica Biosystems) according to ACD protocol. Slides were then removed from the Bond Rx and detection of the C1 signal was performed using the RNAscope 2.5 LS Green Accessory Pack (322550, ACD) according to kit instructions. Slides were heated at 60 °C for 1 h, dipped in xylene and mounted using VectaMount Permanent Mounting Medium (H-5000, Vector Laboratories). The slides were imaged on the Aperio AT2 (Leica Biosystems) to create whole slide images. Images were captured at 40× magnification, with a resolution of 0.25 μm per pixel.

### Inference of effective fission rate from lineage tracing data

The statistical model for crypt fission described in Nicholson et al.^22^ and implemented in the R package RHClones (https://github.com/ElEd2/RHClones) was adapted to infer effective fission rates using counts of clone sizes from *n* = 3 equal size tissue areas from *n* = 3 *Muc2*^het^ and *n* = 3 *Muc2*^hom^ mice with different average *Trop2* expression levels, selected at random. Clone sizes were counted manually using the ImageJ cell counter tool, then the number of pixels stained with Trop2 was obtained using the Area tool in the measure function in ImageJ. Each tissue region was assigned a local fission rate ${\rho }_{i}$ (units, per day) that is sampled from a hierarchical Student’s *T* prior

$${\rho }_{i}\, \sim \,{StudentT}\left(\nu ,\mu ,\sigma \right)$$

with population-level parameters

$$\sigma \,\approx \,\mathrm{normal}\left(0,{10}^{-2}\right)$$

$$\sigma \,\approx \,\mathrm{normal}\,\left(0,{10}^{-2}\right)$$

$$\nu \,\approx \,\mathrm{gamma}\left(2,{10}^{-1}\right).$$

The vector of observed clone sizes, ${{\boldsymbol{g}}}_{i}$, for region *i* is then distributed according to

$${{\boldsymbol{g}}}_{i}\, \sim \,{Multinomial}\left({\boldsymbol{f}}({\rho }_{i},{t}_{i})\right)$$

where

$${{\bf{f}}}_{n}\left(\rho ,t\right)={{\rm{e}}}^{-\rho t}{(1-{{\rm{e}}}^{-\rho t})}^{n-1}$$

is the solution of the Yule–Furry pure birth process, and ${t}_{i}$ is the time (in days) since clone induction in region *i*.

The model for fission inference in this study differs from the model for fission after continuous clone induction described in Nicholson et al.^22^, where this solution is integrated over the age of the individual. However, as in the aforementioned study, a correction was applied to ${f}_{1}$ and ${f}_{2}$ to correct for the chance occurrence of neighboring, nonclonally related crypts. This model does not incorporate crypt fusion, nor does it account for tissue remodeling that may partly explain clone sizes in tissue areas associated with repair following inflammation. In this respect, inferred fission rates should be interpreted as ‘effective’ in the sense that they only capture the value of ${\rho }_{i}$ that is required to explain clone sizes using the Yule–Furry pure birth process introduced in Nicholson et al.^22^.

### Simulations of crypt dynamics

Crypt dynamics were simulated using an on-lattice, voter-type model. The square lattice is of shape M×M. For a site $(i,{j})$ on the lattice, the neighboring sites are defined to be the set

$${N}_{i,j}\,=\left\{\left(i,\,j\,+\,1\right),\,\left(i,j-\,1\right),\,\left(i\,+\,1,\,j\right),\,\left(i-1,j\right),\,\left(i\,+\,1,\,j\,+\,1\right),\,\left(i\,-\,1,\,j-1\right)\right\}$$

resembling a configuration equivalent to a hexagonal lattice in two dimensions with periodic boundary conditions. The state of a lattice site $(i,{j})$, denoted by ${s}_{i,j}$, is defined as ${s}_{i,j}=-1$ for unlabeled (UL) sites, ${s}_{i,j}=0$ for empty sites and ${s}_{i,j}=+1$ for labeled (L) sites. Assuming a labeling efficiency of 20%, the initial population on the lattice consists of 80% of UL and 20% of L sites randomly assigned to each site. The parameters governing the simulations are the fission (${\rho }_{s}$), fusion (${f}_{s}$) and fixation (${P}_{s}$) probabilities of the state s=±1. All are assumed to be neutral and equal for L and UL crypts as the baseline scenario (${\rho }_{s}$, ${f}_{s},\,{{P}}_{s}=\,0.5$ for $s=\,\pm 1$, undefined otherwise). To determine the impact of fission bias in tissue repair, the fission probability of L sites was increased to ${\rho }_{1}=0.95$, with all other parameters remaining the same.

The simulation rules are that, at each time step, we choose a random site $\left(i,{j}\right)$ and update the lattice state according to

1. If $\left(i,\,j\right)$ is an empty site $\left({s}_{i,j}=\,0\right):$

i. All neighbors are empty sites $({s}_{k,l}\,=\,0\,\forall \,\left(k,{l}\right)\in \,{N}_{i,j}\,),$ do nothing;
ii. Otherwise, choose a nonempty neighbor site $\left(k,{l}\right)\in {N}_{i,{j}}:\,{s}_{k,{l}}\ne 0$ at random to undergo fission by setting ${s}_{i,{j}}={s}_{k,l}$ with probability ${\rho }_{{s}_{k,l}}$ (Fig. 1a and Supplementary Fig. 1).

2. If $(i,{j})$ is nonempty$\,{(s}_{i,j}\ne 0)$, then choose a neighbor $\left(k,{l}\right)\in {N}_{i,{j}}$ at random:

i. If $\left(k,{l}\right)$ is empty, then $(i,{j})$ undergoes fission by setting ${s}_{k,l}={s}_{i,j}$ with probability ${\rho }_{{s}_{i,j}}$;
ii. Otherwise, fusion occurs with probability $\frac{{f}_{{s}_{i,j}}+{f}_{{s}_{k,l}}}{2}$ by choosing $\left(h,{w}\right)\in \{\left(i,j\right),(k,l)\}$ with equal probability and setting ${s}_{h,w}=0$. The outcome of monoclonal fixation following fusion is then determined to be the state $s{\prime}$ according to the fixation probabilities ${P}_{{s}_{i,j}}$ and ${P}_{{s}_{k,l}}$ as outlined in Fig. 1b,c and Supplementary Fig. 1 and then setting ${s}_{p,q}=s{\prime}$ where $\left(p,{q}\right)\in \{\left(i,j\right),\left(k,l\right)\}\backslash \{\left(h,{w}\right)\}$.

We note that our definition of homeostasis in in silico models is not equivalent to an equilibrium since, while the average numbers of labeled and unlabeled crypts remain constant, their spatial distribution across the lattice undergoes domain coarsening as captured by a ‘site aggregation’ metric that slowly increases across the course of simulations (Fig. 1f,l). Furthermore, fission-biased conditions lead to a nonhomeostatic system even in the absence of damage, as higher expansion rates generally outpace the creation of empty space by crypt fusion. This is reminiscent of a regime where additional mechanisms, such as diffusion (not considered here), may be required to spatially accommodate mutant crypts with higher rates of fission (Olpe et al.^21^).

### Calculation of site aggregation

The number of distinct neighbor pairs (DNPs) was quantified to represent aggregation of labeled and unlabeled sites in the lattice. The number of the DNPs for a $(i,{j})$ on the lattice is defined to be the cardinality of the set

$${G}_{i,j}=\left\{\left({s}_{i,j}\,,\,{s}_{k,l}\right):\left(k,\,l\right)\in \,{N}_{i,j}\,,\,{s}_{i,j}\,\ne \,{s}_{k,l},\,{s}_{i,j}\,\ne 0,\,{s}_{k,l}\ne 0\right\}.$$

The neighbor pairs of a site (*i, j*) is the set:

$${K}_{i,j}=\left\{\left({s}_{i,j}\,,\,{s}_{k,l}\right):\left(k,\,l\right)\in \,{N}_{i,j}\,,\,{s}_{i,j}\,\ne 0,\,{s}_{k,l}\ne 0\right\}.$$

To obtain a measure of DNPs independent of grid size, we consider the concentration of DNPs, defined as

$$C=\,\frac{\alpha }{\beta }$$

where α and β are the total number of DNPs and neighbor pairs over the whole lattice, respectively. Importantly, we considered aggregation of labeled and unlabeled sites, excluding empty sites and plotted site aggregation as 1−C, so that increasing values denote a decreasing concentration of DNPs.

### Bulk RNA sequencing

RNA was collected from *n* = 3 *Muc2*^hom^ and *n* = 3 *Muc2*^het^ mouse colons from 10-month-old littermates. mRNA extraction was performed following instructions from the extraction kit (180244, Qiagen). Library prep was performed by the CRUK Cambridge Institute Genomics Core using the Illumina Stranded mRNA Prep kit (20040532, Illumina) according to manufacturer’s instructions. Samples were sequenced using the Illumina Novaseq platform with 50-bp paired end reads. Differential expression analysis was performed using DESeq2. Expressed genes were ranked by descending log_2_ fold change for use in gene set enrichment analysis^60^.

### Setup for paired spatial transcriptomic and DNA sequencing

A cohort of five *Muc2*^hom^ mice were treated with ENU 8 weeks post birth and aged for 16 more weeks before collection. Whole-mounted colons were fixed, and six serial tissue sections of 3–5-μm thickness were cut at the crypt base, for at least three areas per colon representing varied levels of pathology. Levels were used for: (1) spatial transcriptomics profiling, (2) pathology assessment with H&E, (3) tumor counting with β-catenin immunohistochemistry, (4) clone counting with GFP, RFP + DAPI and E-cadherin counterstain, and (5) Arid1a-mutated clone counting with Arid1a + DAPI counterstain.

### Spatial transcriptomics

Slide processing and library preparation were performed according to the 10x Visium Cytassist FFPE protocol. In brief, 5-μm sections of tissue were transferred to fit each of the two 11-m^2^ oligo-barcoded capture areas on Visium 10x Genomics slides. Libraries were processed by the CRUK Cambridge Institute Genomics Core according to the manufacturer’s instructions and sequenced on Illumina’s NovaSeqX Plus to an average depth of 8 million mapped reads per sample. Fastq files were processed using the Spaceranger command line tool (10x Genomics v3.0.1) and mapped to the pre-built mm10 reference genome. Processed gene expression matrices for each slide were converted to a Seurat object. Number of counts and features were capped at a minimum of 500 and 200, respectively, to remove low quality spots. After normalization by variance stabilizing transformation using SCTransform, objects corresponding to each slide were integrated into a merged Seurat object using Harmony^61^. Clusters were identified using FindNeighbors using integration anchors at a UMAP dimension of 0.5. Genes differentially expressed in each cluster compared with all other clusters were derived using FindMarkers. To select top markers for each cluster, differentially expressed (DE) genes were filtered for expression in at least 50% of barcoded spots in one cluster (pct.1 >0.5) and expression in less than 50% of other clusters (pct.2 <0.3). This filtered list was ranked on descending log_2_ fold change and ascending *P* value and the top two markers per cluster were selected. For pseudo-bulk analysis, LoupeBrowser was used to extract barcoded spots assigned to each of the 2-mm-diameter biopsies from which DNA was sequenced.

### Transfer of cluster labels

Cluster identities from a reference sample (SITSA1) were transferred to query samples (*Muc2*^het^ and *Muc2*^hom^) using Seurat’s label transfer workflow. Reference and query objects were merged, SCT-normalized and PCA and UMAP dimensionality reduction were performed. The merged object was split by sample, and for each query, transfer anchors were computed on shared features using FindTransferAnchors. Cluster labels from the reference were then predicted with TransferData and added to each query as metadata. Query samples were saved by cohorts for downstream analysis.

### DWLS cell-type deconvolution

Cell-type proportions in each barcoded spot were estimated using DWLS^38^. Cell-type signatures were derived from a single-cell RNA-sequencing dataset of ten mouse colons in different health conditions: *n* = 3 healthy, *n* = 3 acute DSS colitis and *n* = 4 chronic DSS colitis^39^ (accession GSE264408), using the author curated ‘major’ cell-type annotations with the top ten markers for each cell type. The average proportion of each cell type in each cluster was calculated by taking the mean of the DWLS-estimated fractions across all spots assigned to that cluster. Of note, the cellular origin of the tumor cluster 10 could not be appropriately defined using this healthy cell-type reference.

### GSVA

Variation in pathway activity between clusters was quantified using GSVA. Mouse Hallmark gene sets were obtained from MSigDB, excluding sets with fewer than five genes. SCT-normalized expression data was used, after removing genes with near-zero variance (expressed in ≤1 spot). Pathway scores were scaled before downstream analyses. Differential pathway activity for each cluster was identified by comparing that cluster against all other clusters, using FindAllMarkers without thresholds for log fold change or minimum expression.

### Correlation in cluster coverage

Correlations between proportions of barcoded spots mapping to the tumor cluster and other clusters were calculated based on symmetric balances for compositional parts data, as described by Kynclova, Hron and Filzmoser^41^. For each tumor and nontumor cluster pair, two sets of orthonormal coordinate systems were constructed according to symmetric balances of log ratios of either cluster relative to all other clusters in each sample. Pearson correlation coefficients for the first coordinate in each of the two coordinate systems then captures the association of nontumor cluster with the tumor cluster across samples: a positive correlation coefficient implies that enrichment of the two clusters over the respective ‘average representatives’ of other clusters increase simultaneously and vice versa for negative correlation. A coefficient of zero implies that enrichment of these two clusters is controlled by uncorrelated processes.

### Pseudo-spatiotemporal mapping

SpaceFlow^42^ was used to combine expression matrices and spatial coordinates from spatial transcriptomics data in a graph-convolutional deep model, to produce low-dimensional embeddings reflecting both gene expression similarity and spatial proximity between barcoded spots. Pseudo-spatiotemporal maps (pSM) are a pseudotime-like ordering of the clusters according to the embeddings, as described in the original SpaceFlow paper.

### Cluster correlation and matching for additional tumorigenesis models

Gene expression was correlated between clusters identified in the *Muc2*^hom^ + ENU dataset, and the dataset integrating the *Muc2*^hom^ + ENU cohort, as well as DSS + ENU, *Apc* + ENU, *Muc2*^hom^ and *Muc2*^het^ cohorts. For each dataset, average expression profiles were computed using Seurat (v5) and restricted to the set of DE genes detected in both datasets. DE genes (*P* value <0.05) were identified using Seurat’s FindAllMarkers function. To ensure comparability, the number of DE genes used per cluster was limited to 124, the smallest number of DE genes observed in any cluster in the reference dataset.

Pairwise Spearman correlation coefficients were computed between all ‘old’ (*Muc2*^hom^ + ENU) and ‘new’ (full dataset) cluster mean expression vectors. For each old cluster, new clusters exhibiting a correlation coefficient <0.8 were identified as matched. Cluster correspondences were visualized using an alluvial plot generated with the ggalluvial R package (v0.12.5).

### NGS library preparation with targeted DNA sequencing library assays

Genes of interest were imported in the Fluidigm D3 Assay design platform and dual coverage primers were designed (Supplementary Table 7). Eight amplicon pools were prepared and used in the LP 8.8.6 integrated fluidic circuit (101-7663) according to manufacturer’s instructions. Samples were sequenced in two separate runs by the CRUK Cambridge Institute Genomics Core on Illumina NovaSeqX Plus for 150-bp paired end reads.

### Mutation calling

RePlow^62^ was used for mutation calling based on dual replicate amplicon coverage using combinatorial pooling of amplicons and applied to the first 20 million reads of each sample (Supplementary Table 7). In brief, RePlow exploits replicate library preparations to separate the contribution of background errors occurring in library preparation from sequencing errors occurring in sequencing. This is done with a statistical model that calculates the total log ratio of probabilities for variant candidates compared with background errors across all replicates simultaneously. In doing so, adjusted VAFs are constructed by subtracting the contribution from sequencing errors, whereas background error profiles for the error model are calculated independently for the six base pair substitution types (A > C, A > G, A > T, C > A, C > G, C > T) across the targeted regions. An additional stringent filtering step was applied whereby only variants with a positive log ratio of probabilities in both replicates individually were retained, to exclude false positive calls at low VAF values. Orthogonal validation of mutation calls was done using the Ampliconseq pipeline (https://github.com/crukci-bioinformatics/ampliconseq). In brief, variants are called using Vardict from sequence reads aligned to the reference genome (GRCm39). The pipeline models the background substitution noise at each amplicon position to identify and filter SNV calls with an allele fraction indistinguishable from noise. A minimum of 0.01% VAF was set as the lower detection threshold. Outliers in the VAF/noise threshold plane, corresponding to remaining artefacts and germline SNVs were removed post hoc.

### Tessellation algorithm for clone calling

To parsimoniously assign multiple calls of the same mutation made from the same piece of colon tissue to individual clones, as well as resolve the spatial context of the colon that was opened longitudinally before biopsy sampling, a Voronoi tessellation with periodic boundary conditions along the radial axis of each tissue section was calculated using the spatial coordinates of the corresponding biopsies. A depth-first-search algorithm was then used to call single clones by enumerating all connected components of the graph with edges defined by adjacent Voronoi tiles (or those found within 2 mm from one another) containing the given mutation.

### Quantifying selection using dN/dS

The latest version of the maximum likelihood implementation of dN/dS, as originally described in Martincorena et al.^46^ and available in the *R* package dNdScv (https://github.com/im3sanger/dndscv), was applied to identify genes under positive selection. A custom reference object was built from the assembly GRCm39 by subsetting the trinucleotide context-dependent substitution consequence matrix to the set of possible mutations based on the region of the genome selected for targeted sequencing in this study. Counts of mutations fed into the algorithm were those based on individual clones, as defined above.

### Statistical analyses and reproducibility

Visualization and statistical analysis of data were performed in the R statistical computing environment (version 2024.04.0 + 735). All experiments were performed on at least three independent biological replicates (three different mice). Micrographs depict representative data derived from at least three independent biological replicates. Normality was assessed using Shapiro–Wilk’s tests and relevant statistical tests applied. Tests and corresponding *P* values are indicated in the figure legends and figures, respectively. Box plots display the distribution of data using the following components: lower whisker show the smallest observation greater than or equal to lower hinge minus 1.5× IQR; lower hinge shows the 25% quantile; the center line shows the median, 50% quantile; the upper hinge shows the 75% quantile; the upper whisker shows the largest observation less than or equal to upper hinge plus 1.5× IQR.

### Reporting summary

Further information on research design is available in the Nature Portfolio Reporting Summary linked to this article.

## Online content

Any methods, additional references, Nature Portfolio reporting summaries, source data, extended data, supplementary information, acknowledgements, peer review information; details of author contributions and competing interests; and statements of data and code availability are available at 10.1038/s41588-026-02673-0.

## Supplementary information

Supplementary Information Supplementary Fig. 1. Possible outcomes of crypt dynamics simulations.

Reporting Summary

Peer Review File

Supplementary Tables 1–7 Excel file containing Supplementary Tables 1–7 as separate tabs: Supplementary Table 1. Muc2^hom^ + ENU Visium cluster markers. Supplementary Table 2. GSVA for clusters. Supplementary Table 3. All Visium cluster markers. Supplementary Table 4. Targeted amplicon sequencing gene panel. Supplementary Table 5. Unique mutations detected. Supplementary Table 6. Tumor-associated mutations. Supplementary Table 7. Pooling of amplicons for RePlow.

## Data Availability

The RNA-sequencing data generated in the study are publicly available through the Gene Expression Omnibus (GEO) with the accession code GSE297394. The spatial transcriptomics data is publicly available through GEO with the accession code GSE334067. Amplicon sequencing data have been deposited to the Sequence Read Archive (SRA) with BioProject ID PRJNA1366784.
