# Supplementary material for: Paired mutation calling and spatial transcriptomics identify cellular neighborhoods associated with the neoplastic outcome of mouse colitis
Source: Nat Genet. 2026 Jul 28;58(8):1918–27. doi: 10.1038/s41588-026-02673-0 (PMC13447109; doi:10.1038/s41588-026-02673-0)
Supplement: Supplementary file 1 [file 41588_2026_2673_MOESM1_ESM.pdf]

nature genetics

Supplementary information https://doi.org/10.1038/s41588-026-02673-0

Paired mutation calling and spatial transcriptomics identify cellular neighborhoods associated with the neoplastic outcome of mouse colitis

In the format provided by the authors and unedited

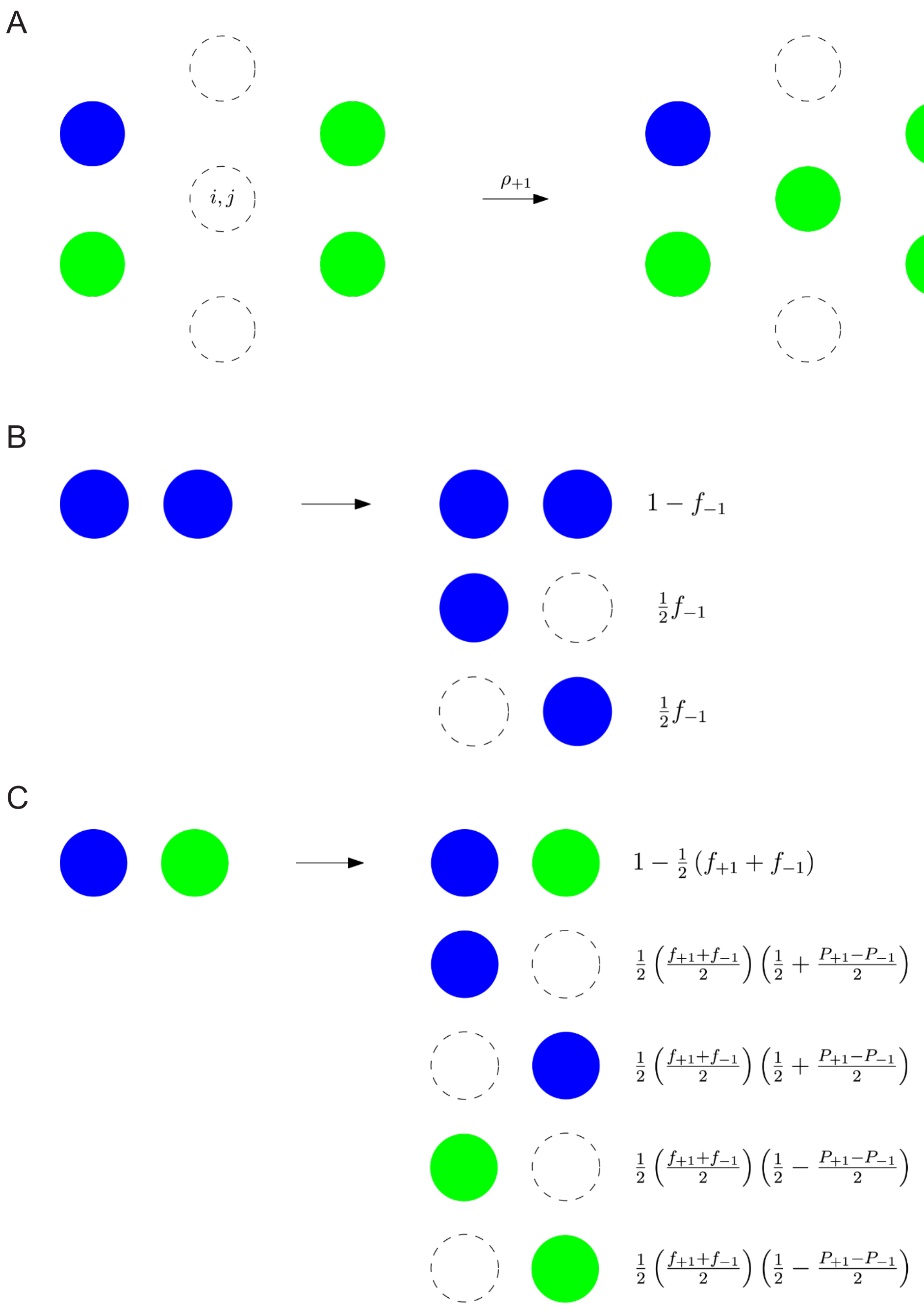

Supplementary Figure 1: Possible outcomes of crypt dynamics simulations. A) Example of a fission event according to Rule A(b) or B(a) in the methods section. In this case a labelled site is the chosen neighbour and has probability to undergo fission and occupy the empty site. B) Example of a fusion event between two unlabelled sites according to Rule B(b) (where the state is the same for both the chosen site and chosen neighbour). In the first case, no fusion occurs. In the second and third case, fusion occurs and leaves behind an empty site. C) Possible outcomes of fusion and monoclonal conversion according to Rule B(b) where the chosen site and its chosen neighbour have different states. In the first case, no fusion occurs. In the remaining cases, fusion occurs with monoclonal conversion determining the final state according to the fixation probabilities as shown.
