# Supplementary material for: Paired mutation calling and spatial transcriptomics identify cellular neighborhoods associated with the neoplastic outcome of mouse colitis
Source: Nat Genet. 2026 Jul 28;58(8):1918–27. doi: 10.1038/s41588-026-02673-0 (PMC13447109; doi:10.1038/s41588-026-02673-0)
Supplement: Supplementary file 2 [file 41588_2026_2673_MOESM2_ESM.pdf]

nature portfolio

Corresponding author(s): Douglas J. Winton, David S. Tourigny

Last updated by author(s): Nov 14, 2025

Reporting Summary

Nature Portfolio wishes to improve the reproducibility of the work that we publish. This form provides structure for consistency and transparency in reporting. For further information on Nature Portfolio policies, see our Editorial Policies and the Editorial Policy Checklist.

Statistics

For all statistical analyses, confirm that the following items are present in the figure legend, table legend, main text, or Methods section.

n/a	Confirmed	
☐	☒	The exact sample size (*n*) for each experimental group/condition, given as a discrete number and unit of measurement
☐	☒	A statement on whether measurements were taken from distinct samples or whether the same sample was measured repeatedly
☐	☒	The statistical test(s) used AND whether they are one- or two-sided<br>*Only common tests should be described solely by name; describe more complex techniques in the Methods section.*
☒	☐	A description of all covariates tested
☐	☒	A description of any assumptions or corrections, such as tests of normality and adjustment for multiple comparisons
☐	☒	A full description of the statistical parameters including central tendency (e.g. means) or other basic estimates (e.g. regression coefficient) AND variation (e.g. standard deviation) or associated estimates of uncertainty (e.g. confidence intervals)
☐	☒	For null hypothesis testing, the test statistic (e.g. *F*, *t*, *r*) with confidence intervals, effect sizes, degrees of freedom and *P* value noted<br>*Give P values as exact values whenever suitable.*
☒	☐	For Bayesian analysis, information on the choice of priors and Markov chain Monte Carlo settings
☒	☐	For hierarchical and complex designs, identification of the appropriate level for tests and full reporting of outcomes
☐	☒	Estimates of effect sizes (e.g. Cohen's *d*, Pearson's *r*), indicating how they were calculated

Our web collection on statistics for biologists contains articles on many of the points above.

Software and code

Policy information about availability of computer code

Data collection

Microscopy and scoring:
Leica SP5 TCS confocal microscope with LAS X software (v2.8.0, Leica)
QuPath (v.0.4.3, https://github.com/qupath/qupath)
ImageJ2 (version 2.14.0/1.54f)

Amplicon design:
Standard Biotool D2 Design Software (https://d3.standardbio.com/account/login)

Data analysis

Analyses were performed predominantly with R version 3.6.1 to current version 2024.04.0+735

RNA sequencing analysis:
MutiQC version 1.9
Picard version 2.18.24
DeSeq2 version 3.19

Effective fission inference:
RHClones (https://github.com/ElEd2/RHClones)

Mutational analysis:
RePlow (https://sourceforge.net/p/replow/wiki/Home/)
AmpliconSeq (https://github.com/crukci-bioinformatics/ampliconseq)

dndscv.0.1.0 (https://github.com/im3sanger/dndscv)

Spatial transcriptomics analysis:
Seurat v4 (https://satijalab.org/seurat/)
Harmony v0.1 (https://github.com/immunogenomics/harmony/tree/master)
SpaceFlow (https://github.com/hongleir/SpaceFlow)
Slingshot (https://github.com/kstreet13/slingshot)
Giotto (https://github.com/drieslab/Giotto)
DWLS (https://github.com/dtsoucas/DWLS)

For manuscripts utilizing custom algorithms or software that are central to the research but not yet described in published literature, software must be made available to editors and reviewers. We strongly encourage code deposition in a community repository (e.g. GitHub). See the Nature Portfolio guidelines for submitting code & software for further information.

Data

Policy information about availability of data

All manuscripts must include a data availability statement. This statement should provide the following information, where applicable:

- Accession codes, unique identifiers, or web links for publicly available datasets
- A description of any restrictions on data availability
- For clinical datasets or third party data, please ensure that the statement adheres to our policy

The RNA-sequencing data generated in the study are publicly available through the Gene Expression Omnibus (GEO) with the accession code GSE297394. Amplicon sequencing data have been deposited to the Sequence Read Archive (SRA) with BioProject ID PRJNA1366784. The spatial transcriptomic data are also available via figshare at https://figshare.com/s/d103da2d0d624cd87548.

Research involving human participants, their data, or biological material

Policy information about studies with human participants or human data. See also policy information about sex, gender (identity/presentation), and sexual orientation and race, ethnicity and racism.

Reporting on sex and gender	1 male
Reporting on race, ethnicity, or other socially relevant groupings	NA
Population characteristics	Age 76
Recruitment	Informed consent was obtained and no monetary compensation was given.
Ethics oversight	Samples were collected from St James University Hospital Leeds. This study was performed under full local research ethical committee approval (REC 15/WA/0131, 12/LO/1217) according to UK Home Office regulations, with informed consent obtained from all participants. No monetary compensation was given.

Note that full information on the approval of the study protocol must also be provided in the manuscript.

Field-specific reporting

Please select the one below that is the best fit for your research. If you are not sure, read the appropriate sections before making your selection.

☒ Life sciences ☐ Behavioural & social sciences ☐ Ecological, evolutionary & environmental sciences

For a reference copy of the document with all sections, see nature.com/documents/nr-reporting-summary-flat.pdf

Life sciences study design

All studies must disclose on these points even when the disclosure is negative.

Sample size	Formal sample size and power analysis were not performed due to the lack of extensive a priori data and uncertain estimates of effect size with this model. Instead, the sample sizes took into consideration preliminary effect sizes from pilot experiments and balanced the 3Rs (Replacement, Reduction and Refinement) to limit the unnecessary use of animals.
Data exclusions	No data were excluded, unless mentioned otherwise.
Replication	Number of animals used for each experiment is mentioned in figure legends, including at least 3 independent animals per experiment.<br>Clone counting = 3 independent animals per condition<br>Bulk RNA sequencing = 3 independent animals per condition<br>Paired spatial transcriptomics and mutation calling: 5 Muc2hom+ENU, n=3 Muc2hom, n=3 Muc2het, n=3 ENU+DSS D7, n=3 DSS+ENU D26, n=4 Apc het + ENU

Randomization	Control and experimental animals were co-housed independent of genotype and cohorts were comprised of a balance of both male and female animals. In order to reduce the impact of covariates such as gender or housing, animals were recruited to treatment groups in a partially randomised manner while taking these factors into account.
Blinding	For animal welfare reasons, researchers were not blinded to genotype during study and data collection

Reporting for specific materials, systems and methods

We require information from authors about some types of materials, experimental systems and methods used in many studies. Here, indicate whether each material, system or method listed is relevant to your study. If you are not sure if a list item applies to your research, read the appropriate section before selecting a response.

Materials & experimental systems

n/a	Involved in the study	
☐	☒	Antibodies
☒	☐	Eukaryotic cell lines
☒	☐	Palaeontology and archaeology
☐	☒	Animals and other organisms
☒	☐	Clinical data
☒	☐	Dual use research of concern
☒	☐	Plants

Methods

n/a	Involved in the study	
☒	☐	ChIP-seq
☒	☐	Flow cytometry
☒	☐	MRI-based neuroimaging

Antibodies

Antibodies used	Antibody, catalog number and dilutions used for immunohistochemistry or immunofluorescence included in the manuscript.<br>β-catenin (0.25 ug/ml, mouse, 610154, BD Biosciences)<br>RFP (1:100, rabbit, R10367, Thermo Fisher)<br>GFP (1:100, chicken, ab13970, Abcam)<br>E-cadherin (1:200, mouse, 610182, BD Biosciences<br>Trop2 (1:100, goat, AF1122, R&D systems)<br>Arid1a (1:100, rabbit, 12354S, Cell Signaling)<br><br>Secondary antibodies:<br>Rabbit anti-mouse (ab125913, Abcam) at 1:500<br>Donkey anti-rabbit (A31572, Thermofisher)<br>Donkey anti-goat A21447 , Thermofisher)<br>Donkey anti-chicken (703-6-5-155, Thermofisher)<br>Donkey anti-mouse (ab150109, Abcam)
Validation	1. β-catenin used for IHC. Species reactivity: Human, Mouse, Rat, Dog, Chicken. This antibody was validated by the company and used in our previous study (PMID: 33093165).<br>2. RFP used for IF. Species independent. This antibody was validated by the company.<br>3. GFP used for IF. Species independent. This antibody was validated by the company.<br>4. E-cadherin used for IF. This antibody was validated by the company and used in our previous study (PMID: 38199279)<br>5. Trop2 used for IF. Species independent. This antibody was validated by the company.<br>6. Arid1a used for IF. Species independent. This antibody was validated by the company and used in our previous study (PMID: 39920335).

Animals and other research organisms

Policy information about studies involving animals; ARRIVE guidelines recommended for reporting animal research, and Sex and Gender in Research

Laboratory animals	Mice of both sexes aged at least 8 weeks old were induced. C57/BL6J mice with a combination of VillinCreERT2, Muc2KO or Apc het and R26R-Confetti alleles were used
Wild animals	No wild animals were used in the study.
Reporting on sex	Both male and female mice were used in all experiments.
Field-collected samples	No field-collected samples were used in this study.
Ethics oversight	All animal experiments were performed in accordance with UK Home Office regulations (under project licence PD5F099BE). They adhered to the ARRIVE guidelines and were subject to review by the Animal Welfare and Ethical Review Board of the CRUK Cambridge Institute.

Note that full information on the approval of the study protocol must also be provided in the manuscript.

Plants

Seed stocks	NA
Novel plant genotypes	NA
Authentication	NA
