# Supplementary material for: Paired mutation calling and spatial transcriptomics identify cellular neighborhoods associated with the neoplastic outcome of mouse colitis
Source: Nat Genet. 2026 Jul 28;58(8):1918–27. doi: 10.1038/s41588-026-02673-0 (PMC13447109; doi:10.1038/s41588-026-02673-0)
Supplement: Supplementary file 3 [file 41588_2026_2673_MOESM3_ESM.pdf]

nature portfolio

Peer Review File

Paired mutation calling and spatial transcriptomics identify cellular neighbourhoods dictating the neoplastic outcome of colitis

Corresponding Author: Dr Douglas Winton

Version 0:

Decision Letter:

13th Jun 2025

Dear Dr Winton,

First, please forgive me for the delay in returning this decision to you. Thank you for your patience.

Your Article, "Paired mutation calling and spatial transcriptomics identify cellular neighbourhoods dictating the neoplastic outcome of colitis" has now been seen by 4 referees. You will see from their comments below that while they find your work of interest, some important points are raised. We are interested in the possibility of publishing your study in Nature Genetics, but would like to consider your response to these concerns in the form of a revised manuscript before we make a final decision on publication.

We therefore invite you to revise your manuscript taking into account all reviewer and editor comments. Please highlight all changes in the manuscript text file. At this stage we will need you to upload a copy of the manuscript in MS Word .docx or similar editable format.

We are committed to providing a fair and constructive peer-review process. Do not hesitate to contact us if there are specific requests from the reviewers that you believe are technically impossible or unlikely to yield a meaningful outcome.

When revising your manuscript:

*1) Include a “Response to referees” document detailing, point-by-point, how you addressed each referee comment. If no action was taken to address a point, you must provide a compelling argument. This response will be sent back to the referees along with the revised manuscript.

*2) If you have not done so already please begin to revise your manuscript so that it conforms to our Article format instructions, available
here.
Refer also to any guidelines provided in this letter.

*3) Include a revised version of any required Reporting Summary: https://www.nature.com/documents/nr-reporting-summary.pdf
It will be available to referees (and, potentially, statisticians) to aid in their evaluation if the manuscript goes back for peer review.
A revised checklist is essential for re-review of the paper.

Please be aware of our guidelines on digital image standards.

EXTENDED DATA FIGURES

When re-submitting your manuscript, please ensure that any supplementary figures and tables that are crucial to the manuscript’s conclusions are converted into Extended Data figures and tables to increase visibility of these data. Extended Data figures and tables are online-only (present in the online PDF and full-text HTML versions of the paper), peer-reviewed

display items that provide essential background to the article but are not included in the main article due to space constraints. A maximum of ten Extended Data display items (figures and tables) is permitted.

Please use the link below to submit your revised manuscript and related files:

Link Redacted

Note: This URL links to your confidential home page and associated information about manuscripts you may have submitted, or that you are reviewing for us. If you wish to forward this email to co-authors, please delete the link to your homepage.

We hope to receive your revised manuscript within four to eight weeks. If you cannot send it within this time, please let us know.

Please do not hesitate to contact me if you have any questions or would like to discuss these revisions further.

Nature Genetics is committed to improving transparency in authorship. As part of our efforts in this direction, we are now requesting that all authors identified as 'corresponding author' on published papers create and link their Open Researcher and Contributor Identifier (ORCID) with their account on the Manuscript Tracking System (MTS), prior to acceptance. ORCID helps the scientific community achieve unambiguous attribution of all scholarly contributions. You can create and link your ORCID from the home page of the MTS by clicking on 'Modify my Springer Nature account'. For more information please visit please visit www.springernature.com/orcid.

We look forward to seeing the revised manuscript and thank you for the opportunity to review your work.

Sincerely,

Safia Danovi, PhD
Senior Editor, Nature Genetics
ORCiD: 0009-0007-7822-5479

Referee expertise:

Referee #1: intestinal mucosal immunity

Referee #2: CRC, -omics

Referee #3: intestinal mucosal immunity, ST

Referee #4: CRC, in vivo models

Reviewers' Comments:

Reviewer #1 (Remarks to the Author):

During the transition from Inflammatory Bowel Disease to cancer, the mutational profile undergoes a fundamental shift: cells initially acquire mutations in inflammation-related genes, but over time, cancer-driving mutations become dominant. The cellular microenvironment's influence on how these different mutated cell populations establish, survive, and expand—and consequently how this impacts cancer development in colitis—remains poorly understood.

To address this question, authors employed a comprehensive experimental approach combining live cell tracking, computational modeling, mutation analysis, and spatial gene expression mapping in a mouse model of inflammation-induced colon cancer. This allowed them to monitor the fate of mutated cell clones throughout chronic inflammation. Their analysis revealed distinct tissue neighborhoods enriched with either epithelial cells or immune cells. They propose that when tissues shift toward a repair-focused state, this microenvironment inadvertently promotes cancer initiation. Specifically, reparative conditions favor the survival and growth of cells carrying cancer-promoting mutations while simultaneously reducing the presence of inflammation-resistant tissue areas that would otherwise harbor non-cancerous mutated cells.

These findings suggest that the tissue's attempt to heal from chronic inflammation creates conditions that accelerate the selection of cancer-driving mutations, providing new insight into how inflammatory diseases progress to malignancy.

To broaden the conclusions, I think the manuscript will benefit greatly from including another more common model of CAC, like the AOM-DSS model, that has a well-defined trajectory of inflammatory changes followed by tumor development.

Furthermore, the majority of colorectal cancer is not associated with IBD in humans. However, 90% of CRC is associated to some extent with infiltration of immune cells. Therefore, comparing the expression and mutation neighborhoods that develop

in the scenario where tumorigenesis precedes inflammation, like the CPC-APC model, will be very interesting. Could such a scenario explain the accumulation of Arid1a mutations in human CRC? And will similar immune-exclusion zones, IL-17RC mutations or neutrophil-cluster association with tumors be detected? I think these studies will potentially answer the fundamental questions of tumors arising because of aberrant, chronic inflammation or the tumor itself subverting the inflammatory process.

Reviewer #2 (Remarks to the Author):

Moutin and colleagues have employed a genetically engineered confetti mouse model, focusing on the loss of Mucin 2 (Muc2), to investigate clonal expansion in colitis. They further incorporate the chemical mutagen ENU to simulate colitis-associated tumorigenesis. Through robust molecular characterization, utilizing spatial transcriptomics and genetic profiling, the authors propose that specific cellular neighborhoods, indicative of reparative tissue environments, play an important role in facilitating tumor initiation. The manuscript is well-written and advances our understanding of neoplastic progression in the context of inflammation.

However, several issues need addressing or further clarification based on the data provided.

- The authors discuss the DSS-induced colitis model and the emergence of a fetal-like state. They note similar enrichment patterns in their Muc2het vs. Muc2ko model using GSEA (Figure 1G). The Trop2 IF (Figure 1H) suggests these fetal-like cells are mutually exclusive concerning their labeled clones. Could the authors elaborate on this observation?
- The authors utilize Visium to explore cellular neighborhoods within the Muc2ko model. However, the dataset presented includes only slides from the Muc2ko model. Incorporating Muc2het and/or WT samples would allow for comparisons against a non-colitis background. Is neighborhood Cluster 4 unique to the Muc2ko model compared to Muc2het or WT?
- Presumably, the reparative Cluster 4 is present in Muc2ko tissues before ENU treatment. What, if any, are the differences between the Trop2 positive neighborhoods identified in Figure 1H, and the neighborhoods of Cluster 4 described in Figure 2? Profiling Muc2ko samples without ENU treatment could help to resolve this.
- In Figure 4B, the authors identify numerous tumor-associated mutations within clusters 4 and 10, suggesting a mechanistic link explaining tumor initiation in repairing epithelium during chronic inflammation. However, the system's dynamics remain unclear. Is cluster 4 a precursor to cluster 10? Does this transition require additional genetic alterations, or does it indicate differing stages of tumorigenesis? Analyzing tissues across multiple time points might help clarify this matter.
- What factors do the authors suggest could render reparative niches more prone to acquiring oncogenic mutations?
- Further incorporation of human samples to corroborate the key findings would significantly lift the impact of the study.
- Is the expression module of Cluster 4 prognostic for the development of colitis associated cancer in humans?

Minor Points:

- In Figure 1G, the P-value is stated as '0.000'. This seems to be a rounding issue and should be expressed in scientific notation to convey the precise value.
- In Figure 2E, several paired GSEA comparisons are presented. Given the intention to compare multiple groups, the authors might consider employing GSVA for this analysis.
- For Figure 2, a deconvolution analysis using scRNAseq as a reference could enhance the interpretation of cellular neighborhoods.

Reviewer #3 (Remarks to the Author):

While the topic is highly relevant and the authors employ a broad range of methods, the current manuscript lacks the mechanistic rigor, analytical depth, and conceptual innovation expected for publication in Nature Genetics. The findings remain largely descriptive, speculative, and insufficiently validated. For these reasons, I do not recommend the manuscript for publication.

Reviewer #4 (Remarks to the Author):

The manuscript “Paired mutation calling and spatial transcriptomics identify cellular neighbourhoods dictating the neoplastic outcome of colitis” assesses the mutational and transcriptional landscape of tissue progressing from IBD tissue to IBD-associated cancer. The work and investigative/analytical approach adopted in this manuscript are both interesting and very worthy of publication. Whilst many of the key findings are not entirely novel (e.g. inflammation promotes expansion of pro-tumorigenic clones, the association between inflammation duration and clonal expansion), the study provides robust evidence supporting the clonal dynamics underlying the previously established molecular pathogenesis of colitis-associated colorectal cancer (CRC). This contributes valuable confirmation and clarity to the existing body of knowledge.

Overall manuscript comments
Overall, the manuscript is well-written and easy to follow. However, the final sections discussing the analysis of the mutational landscape are somewhat more complex and could benefit from simplification to improve readability and

comprehension.

Specific comments
Figure 1a, 1b and 1c / Lines 104-112
• Could the author explain the reason behind their choice to use Muc2het mice as controls instead of Muc2Wt. Do Muc2het mice get any inflammation? If yes, would a Muc2wt mouse be a better baseline control? Moreover, could the comparison between Muc2het and Muc2hom used to infer on the relationship between inflammation extent and clonal expansion (as we already know that in humans more severe inflammation is associated with great CRC risk)?
Figure 1e and 1f:
• The labelling and colouring used in Figures 1E and 1F is slightly confusing. My understanding is that the grey colour represents the undamaged grid, which also corresponds to the grid under homeostatic conditions. In Figure 1F (left), the grey line appears to represent both the undamaged grid from Figure 1E and the grid prior to the application of damage. If this is correct, then in Figure 1F (right), the grey line should correspond to the undamaged/homeostatic condition, and the red line should represent the damaged/regenerating condition. Could you please clarify if this interpretation is accurate?
o Could the authors consider making the figure legends for Figures 1E and 1F slightly more detailed to better guide the reader through the data presentation? A similar suggestion applies to Figures 1K and 1L, where additional explanation would help improve clarity and aid interpretation.
• If my understanding of Figure 1F (right) is correct, the persistence of the damaged grid/line is expected and illustrates that, following an initial increase in site aggregation, this begins to decline over time. If the simulation was extended further, would the level of aggregation eventually plateau at the same level as the undamaged grid, or would it stabilise at a new, elevated homeostatic state? (hinting at a form of long-term memory following damage)
• Typo in line 131
Supplementary Figure 1:
• Statistical analysis appears to be missing from the survival plot.
• What does the Muc2het small bowel look like?
Lines 139-144:
• While the findings are certainly interesting, the conclusions regarding increased crypt fission are not particularly novel, as increased crypt fission has been previously reported in the context of both regeneration and IBD. Nonetheless, the authors present elegant proof of concept experiments that reinforce and illustrate these observations effectively.
Lines 154-157:
• The authors state "This highlight how tissue repair conditions can accelerate and promote the accumulation and expansion of mutant clones in damaged tissue." in relation to their in-silico observation of fission-biased clones. Would it be more correct to state that the data highlights how tissue repair conditions can accelerate and promote the accumulation and expansion of clones with a proliferative (or fission biased) advantage rather mutant clones in general.
Lines 169-172
• Should these lines be merged with the next paragraph?.
Identification of cellular neighbourhoods in colitis-associated neoplasia/biological processes defining epithelial neighbourhoods
• The authors should be commended for their elegant experimental design, robust analytical approach, and their ability to extract key insights into clonal dynamics and cellular neighbourhoods from spatial transcriptomics data.
Line 205
• Typo in neighbourhood
Lines 188-200
• The authors report: high, low, abundant… expression of the different keratins. How is high/low being defined? Is there a numerical/statical value that is being used as cut off. As for example, visually cluster 11 also appears to be enriched for keratin 7 and keratin 14
Figure 2g / Lines 237-242:
• The association between regenerative epithelium and tumorigenic tissue is not a novel finding, especially in the context of IBD-CRC, as previously reported by Gil Vazquez et al., 2022. Nevertheless, the current study provides valuable proof of this relationship at the cellular level.
Figure 3 / Lines 268-289
• The mutational landscape detected in the Muc2hom + ENU mice has some clear differences from that reported in human IBD-CRC (e.g. Apc highly mutated in the mice, but reported to be mutated only in 10% of IBD-CRC, p53 highly mutated in IBD-CRC (>70%) but lowly mutated in the mice). Can the authors comment on this. While the overall message with regards to association of specific mutational signatures with specific clusters remains intact, from a mutational point of view is this perhaps not the best model of IBD-CRC? Does the Muc2hom + ENU model at the genetic level represent more of a model of sporadic CRC with an inflammatory push.
• Having said that the findings on Arid1a are compelling and do fit well with the high mutational burden of the gene in human IBD-CRC.
Supplementary Figure 6
• In terms of the tumour associated genes and their distribution: are different Wnt mutations were mutually exclusive?
• Can the association between specific mutations and specific cluster be used to infer a temporal acquisition of the mutations?

Minor Comment:
The introduction is well written and provides a solid background for the manuscript. However, it would benefit from referencing key publications that have previously examined the mutational landscape of colitis-associated colorectal cancer,

such as Baker AM, Cross W, Curtius K, et al. Gut, 2019 Including these studies would help contextualise the current work within the existing literature.

Reviewer #4 (Remarks on figshare data availability):

The data loos robust and agreement with the claims in the paper

Version 1:

Decision Letter:

2nd Dec 2025

Dear Dr Winton,

Thank you so much for sending back the revised version of your manuscript "Paired mutation calling and spatial transcriptomics identify cellular neighbourhoods dictating the neoplastic outcome of colitis".

I was ready to send t back to your reviewers when I realised (with some horror) that I'd made a mistake when sending you my last revise decision.

I won't bore you with the details but, in short, you were sent Reviewer #3's confidential comments instead of their remarks to you and your co-authors. Below, you'll find the feedback that was intended for you to receive.

I can not apologise enough for this. These letters are generated by the system so I don't know what happened but I am so sorry. I've been through their comments and I am hoping that their concerns have largely been addressed by your response to the other reviewers, or may be tackled textually. Please let me know if anything isn't clear.

We therefore invite you to revise your manuscript and your point-by-point letter taking into account these new comments. Please highlight all changes in the manuscript text file. At this stage we will need you to upload a copy of the manuscript in MS Word .docx or similar editable format.

We are committed to providing a fair and constructive peer-review process. Do not hesitate to contact us if there are specific requests from the reviewers that you believe are technically impossible or unlikely to yield a meaningful outcome.

When revising your manuscript:

*1) Include a “Response to referees” document detailing, point-by-point, how you addressed each referee comment. If no action was taken to address a point, you must provide a compelling argument. This response will be sent back to the referees along with the revised manuscript.

*2) If you have not done so already please begin to revise your manuscript so that it conforms to our Article format instructions, available
here.
Refer also to any guidelines provided in this letter.

*3) Include a revised version of any required Reporting Summary: https://www.nature.com/documents/nr-reporting-summary.pdf
It will be available to referees (and, potentially, statisticians) to aid in their evaluation if the manuscript goes back for peer review.
A revised checklist is essential for re-review of the paper.

Please be aware of our guidelines on digital image standards.

EXTENDED DATA FIGURES

When re-submitting your manuscript, please ensure that any supplementary figures and tables that are crucial to the manuscript’s conclusions are converted into Extended Data figures and tables to increase visibility of these data. Extended Data figures and tables are online-only (present in the online PDF and full-text HTML versions of the paper), peer-reviewed display items that provide essential background to the article but are not included in the main article due to space constraints. A maximum of ten Extended Data display items (figures and tables) is permitted.

Please use the link below to submit your revised manuscript and related files:

Link Redacted

Note: This URL links to your confidential home page and associated information about manuscripts you may have

submitted, or that you are reviewing for us. If you wish to forward this email to co-authors, please delete the link to your homepage.

We hope to receive your revised manuscript within four to eight weeks. If you cannot send it within this time, please let us know.

Please do not hesitate to contact me if you have any questions or would like to discuss these revisions further.

Nature Genetics is committed to improving transparency in authorship. As part of our efforts in this direction, we are now requesting that all authors identified as ‘corresponding author’ on published papers create and link their Open Researcher and Contributor Identifier (ORCID) with their account on the Manuscript Tracking System (MTS), prior to acceptance. ORCID helps the scientific community achieve unambiguous attribution of all scholarly contributions. You can create and link your ORCID from the home page of the MTS by clicking on ‘Modify my Springer Nature account’. For more information please visit please visit www.springernature.com/orcid.

I can not apologise enough for this mix-up.

Sincerely,

Safia Danovi, PhD
Senior Editor, Nature Genetics
ORCiD: 0009-0007-7822-5479

Referee expertise:

REVIEWER #1
During the transition from Inflammatory Bowel Disease to cancer, the mutational profile undergoes a fundamental shift: cells initially acquire mutations in inflammation-related genes, but over time, cancer-driving mutations become dominant. The cellular microenvironment's influence on how these different mutated cell populations establish, survive, and expand—and consequently how this impacts cancer development in colitis—remains poorly understood.

To address this question, authors employed a comprehensive experimental approach combining live cell tracking, computational modeling, mutation analysis, and spatial gene expression mapping in a mouse model of inflammation-induced colon cancer. This allowed them to monitor the fate of mutated cell clones throughout chronic inflammation. Their analysis revealed distinct tissue neighborhoods enriched with either epithelial cells or immune cells. They propose that when tissues shift toward a repair-focused state, this microenvironment inadvertently promotes cancer initiation. Specifically, reparative conditions favor the survival and growth of cells carrying cancer-promoting mutations while simultaneously reducing the presence of inflammation-resistant tissue areas that would otherwise harbor non-cancerous mutated cells.

These findings suggest that the tissue's attempt to heal from chronic inflammation creates conditions that accelerate the selection of cancer-driving mutations, providing new insight into how inflammatory diseases progress to malignancy.

To broaden the conclusions, I think the manuscript will benefit greatly from including another more common model of CAC, like the AOM-DSS model, that has a well-defined trajectory of inflammatory changes followed by tumor development.

Furthermore, the majority of colorectal cancer is not associated with IBD in humans. However, 90% of CRC is associated to some extent with infiltration of immune cells. Therefore, comparing the expression and mutation neighborhoods that develop in the scenario where tumorigenesis precedes inflammation, like the CPC-APC model, will be very interesting.
Could such a scenario explain the accumulation of Arid1a mutations in human CRC? And will similar immune-exclusion zones, IL-17RC mutations or neutrophil-cluster association with tumors be detected? I think these studies will potentially answer the fundamental questions of tumors arising because of aberrant, chronic inflammation or the tumor itself subverting the inflammatory process.

REVIEWER #2
Moutin and colleagues have employed a genetically engineered confetti mouse model, focusing on the loss of Mucin 2 (Muc2), to investigate clonal expansion in colitis. They further incorporate the chemical mutagen ENU to simulate colitis-associated tumorigenesis. Through robust molecular characterization, utilizing spatial transcriptomics and genetic profiling, the authors propose that specific cellular neighborhoods, indicative of reparative tissue environments, play an important role in facilitating tumor initiation. The manuscript is well-written and advances our understanding of neoplastic progression in the context of inflammation.

However, several issues need addressing or further clarification based on the data provided.

- The authors discuss the DSS-induced colitis model and the emergence of a fetal-like state. They note similar enrichment patterns in their Muc2het vs. Muc2ko model using GSEA (Figure 1G). The Trop2 IF (Figure 1H) suggests these fetal-like cells are mutually exclusive concerning their labeled clones. Could the authors elaborate on this observation?
- The authors utilize Visium to explore cellular neighborhoods within the Muc2ko model. However, the dataset presented includes only slides from the Muc2ko model. Incorporating Muc2het and/or WT samples would allow for comparisons

against a non-colitis background. Is neighborhood Cluster 4 unique to the Muc2ko model compared to Muc2het or WT?
● Presumably, the reparative Cluster 4 is present in Muc2ko tissues before ENU treatment. What, if any, are the differences between the Trop2 positive neighborhoods identified in Figure 1H, and the neighborhoods of Cluster 4 described in Figure 2? Profiling Muc2ko samples without ENU treatment could help to resolve this.
● In Figure 4B, the authors identify numerous tumor-associated mutations within clusters 4 and 10, suggesting a mechanistic link explaining tumor initiation in repairing epithelium during chronic inflammation. However, the system's dynamics remain unclear. Is cluster 4 a precursor to cluster 10? Does this transition require additional genetic alterations, or does it indicate differing stages of tumorigenesis? Analyzing tissues across multiple time points might help clarify this matter.
● What factors do the authors suggest could render reparative niches more prone to acquiring oncogenic mutations?
● Further incorporation of human samples to corroborate the key findings would significantly lift the impact of the study.
● Is the expression module of Cluster 4 prognostic for the development of colitis associated cancer in humans?

Minor Points:

● In Figure 1G, the P-value is stated as '0.000'. This seems to be a rounding issue and should be expressed in scientific notation to convey the precise value.
● In Figure 2E, several paired GSEA comparisons are presented. Given the intention to compare multiple groups, the authors might consider employing GSVA for this analysis.
● For Figure 2, a deconvolution analysis using scRNAseq as a reference could enhance the interpretation of cellular neighborhoods.

REVIEWER #3 (THESE ARE THE COMMENTS YOU DIDN'T RECEIVE IN THE LAST ROUND)
In this manuscript, Moutin and colleagues investigate the relationship between somatic mutations and clonal fate in colitis-associated tumorigenesis using a combination of in vivo lineage tracing, murine models of intestinal inflammation, spatial transcriptomics, in silico modeling, and histological analyses. This is a timely and important area of research, with potential to yield valuable insights into how chronic inflammation contributes to colorectal cancer (CRC) initiation and progression. However, the current manuscript falls short of providing the necessary mechanistic depth, conceptual clarity, and validation required for publication in Nature Genetics.

While the study integrates several experimental platforms, it lacks a cohesive narrative and fails to generate robust or novel conclusions. Many of the findings are descriptive, speculative, or confirmatory of what is already known in the field, without offering significant new biological insights or resolving fundamental questions about mutation selection and clonal dynamics in inflammatory contexts. For instance, while omics approaches are employed to uncover molecular features, the validation studies rely heavily on already known genes (e.g., Arid1a), rather than identifying and functionally testing novel candidates from their own dataset. The rationale for focusing on certain markers, such as TROP2 or Arid1a, is not clearly supported by the spatial transcriptomics or histological data.

A major concern is that key conclusions are not substantiated by the presented data. For example, the assertion that repair zones favor clonal expansion remains speculative and lacks experimental support. The spatial transcriptomics analysis is underdeveloped, with insufficient quality control, annotation, or validation. The predictive modeling component is also weakly justified and lacks validation, remaining anecdotal in its contribution.

Furthermore, the study design and figure structure suffer from poor organization. Figures 1–4 appear disconnected, and the manuscript reads as a fragmented collection of analyses rather than a focused investigation driven by a central hypothesis. The frequent shifts between different mouse models (Confetti, Muc2 KO, ENU-treated) without adequate characterization further dilute the overall message. The manuscript does not address how mutant clones are selected or maintained in inflammatory settings, nor how these dynamics contribute to CRC progression in IBD patients.

Specific concerns include:
1. The experimental and analytical design lacks clarity, and the manuscript would benefit from schematic overviews to aid reader comprehension.
2. TROP2 analysis is unconvincing; figures selectively highlight favorable data and fail to show unbiased correlation analyses (e.g., Figure 1H–I).
3. Bold claims regarding clonal expansion in regenerative zones are not sufficiently supported by experimental evidence.
4. The Confetti model is introduced but not meaningfully integrated into the remainder of the study.
5. In silico modeling is poorly motivated and lacks empirical validation, reducing its interpretability.
6. Spatial transcriptomic findings are largely descriptive and overlap with prior literature (e.g., immune-rich tumor margins), which are not adequately cited.
7. Annotation of the “inflammation-resistant” cluster (Figure 4) lacks clear rationale or supporting evidence.
8. Use of three distinct mouse models is not well justified and potentially confounds interpretation regarding IBD-related tumorigenesis.
9. Several speculative statements throughout (e.g., lines 250–252, 263) are not backed by data.
10. No meaningful differential gene expression or pathway analysis is presented between genotypes in the Muc2 model.
11. The statement that Arid1a IHC “demonstrates” the expansion of clones in inflammation-resistant regions is overstated.
12. Final conclusions (lines 378–380) are vague and unsupported.
13. Central questions: how inflammation selects mutant clones, and how this impacts tumorigenesis, remain unaddressed.
Minor issues:

• The manuscript is difficult to follow, with poor flow between sections and abrupt transitions. Removal of subheadings (e.g., line 175) may improve readability.
• Several figure panels lack clarity, annotations, and appropriate controls.

REVIEWER #4
The manuscript “Paired mutation calling and spatial transcriptomics identify cellular neighbourhoods dictating the neoplastic outcome of colitis” assesses the mutational and transcriptional landscape of tissue progressing from IBD tissue to IBD-associated cancer. The work and investigative/analytical approach adopted in this manuscript are both interesting and very worthy of publication. Whilst many of the key findings are not entirely novel (e.g. inflammation promotes expansion of pro-tumorigenic clones, the association between inflammation duration and clonal expansion), the study provides robust evidence supporting the clonal dynamics underlying the previously established molecular pathogenesis of colitis-associated colorectal cancer (CRC). This contributes valuable confirmation and clarity to the existing body of knowledge.

Overall manuscript comments
Overall, the manuscript is well-written and easy to follow. However, the final sections discussing the analysis of the mutational landscape are somewhat more complex and could benefit from simplification to improve readability and comprehension.

Specific comments
Figure 1a, 1b and 1c / Lines 104-112
• Could the author explain the reason behind their choice to use Muc2het mice as controls instead of Muc2Wt. Do Muc2het mice get any inflammation? If yes, would a Muc2wt mouse be a better baseline control? Moreover, could the comparison between Muc2het and Muc2hom used to infer on the relationship between inflammation extent and clonal expansion (as we already know that in humans more severe inflammation is associated with great CRC risk)?
Figure 1e and 1f:
• The labelling and colouring used in Figures 1E and 1F is slightly confusing. My understanding is that the grey colour represents the undamaged grid, which also corresponds to the grid under homeostatic conditions. In Figure 1F (left), the grey line appears to represent both the undamaged grid from Figure 1E and the grid prior to the application of damage. If this is correct, then in Figure 1F (right), the grey line should correspond to the undamaged/homeostatic condition, and the red line should represent the damaged/regenerating condition. Could you please clarify if this interpretation is accurate?
o Could the authors consider making the figure legends for Figures 1E and 1F slightly more detailed to better guide the reader through the data presentation? A similar suggestion applies to Figures 1K and 1L, where additional explanation would help improve clarity and aid interpretation.
• If my understanding of Figure 1F (right) is correct, the persistence of the damaged grid/line is expected and illustrates that, following an initial increase in site aggregation, this begins to decline over time. If the simulation was extended further, would the level of aggregation eventually plateau at the same level as the undamaged grid, or would it stabilise at a new, elevated homeostatic state? (hinting at a form of long-term memory following damage)
• Typo in line 131
Supplementary Figure 1:
• Statistical analysis appears to be missing from the survival plot.
• What does the Muc2het small bowel look like?
Lines 139-144:
• While the findings are certainly interesting, the conclusions regarding increased crypt fission are not particularly novel, as increased crypt fission has been previously reported in the context of both regeneration and IBD. Nonetheless, the authors present elegant proof of concept experiments that reinforce and illustrate these observations effectively.
Lines 154-157:
• The authors state “This highlight how tissue repair conditions can accelerate and promote the accumulation and expansion of mutant clones in damaged tissue.” in relation to their in-silico observation of fission-biased clones. Would it be more correct to state that the data highlights how tissue repair conditions can accelerate and promote the accumulation and expansion of clones with a proliferative (or fission biased) advantage rather mutant clones in general.
Lines 169-172
• Should these lines be merged with the next paragraph?.
Identification of cellular neighbourhoods in colitis-associated neoplasia/biological processes defining epithelial neighbourhoods
• The authors should be commended for their elegant experimental design, robust analytical approach, and their ability to extract key insights into clonal dynamics and cellular neighbourhoods from spatial transcriptomics data.
Line 205
• Typo in neighbourhood
Lines 188-200
• The authors report: high, low, abundant… expression of the different keratins. How is high/low being defined? Is there a numerical/statical value that is being used as cut off. As for example, visually cluster 11 also appears to be enriched for keratin 7 and keratin 14
Figure 2g / Lines 237-242:
• The association between regenerative epithelium and tumorigenic tissue is not a novel finding, especially in the context of IBD-CRC, as previously reported by Gil Vazquez et al., 2022. Nevertheless, the current study provides valuable proof of this relationship at the cellular level.

Figure 3 / Lines 268-289
• The mutational landscape detected in the Muc2hom + ENU mice has some clear differences from that reported in human IBD-CRC (e.g. Apc highly mutated in the mice, but reported to be mutated only in 10% of IBD-CRC, p53 highly mutated in IBD-CRC (>70%) but lowly mutated in the mice). Can the authors comment on this. While the overall message with regards to association of specific mutational signatures with specific clusters remains intact, from a mutational point of view is this perhaps not the best model of IBD-CRC? Does the Muc2hom + ENU model at the genetic level represent more of a model of sporadic CRC with an inflammatory push.
• Having said that the findings on Arid1a are compelling and do fit well with the high mutational burden of the gene in human IBD-CRC.
Supplementary Figure 6
• In terms of the tumour associated genes and their distribution: are different Wnt mutations were mutually exclusive?
• Can the association between specific mutations and specific cluster be used to infer a temporal acquisition of the mutations?

Minor Comment:
The introduction is well written and provides a solid background for the manuscript. However, it would benefit from referencing key publications that have previously examined the mutational landscape of colitis-associated colorectal cancer, such as Baker AM, Cross W, Curtius K, et al. Gut, 2019 Including these studies would help contextualise the current work within the existing literature.

Reviewers' Comments:

Version 2:

Decision Letter:

19th Mar 2026

Dear Dr Winton,

First, please accept my sincere apologies for the delay in returning this decision to you. I have been on medical leave for 8 weeks and have only just returned to the office. Thank you so much for bearing with me.

Your Article, "Paired mutation calling and spatial transcriptomics identify cellular neighbourhoods dictating the neoplastic outcome of colitis" has now been seen by 4 referees. You will see from their comments below that while they find your work of interest, some important points are raised. We are interested in the possibility of publishing your study in Nature Genetics, but would like to consider your response to these concerns in the form of a revised manuscript before we make a final decision on publication.

We therefore invite you to revise your manuscript taking into the comments from Reviewer #3. We believe that these can largely be addressed through textual edits rather than extensive additional analyses. Please highlight all changes in the manuscript text file. At this stage we will need you to upload a copy of the manuscript in MS Word .docx or similar editable format.

We are committed to providing a fair and constructive peer-review process. Do not hesitate to contact us if there are specific requests from the reviewers that you believe are technically impossible or unlikely to yield a meaningful outcome.

When revising your manuscript:

*1) Include a “Response to referees” document detailing, point-by-point, how you addressed each referee comment. If no action was taken to address a point, you must provide a compelling argument. This response will be sent back to the referees along with the revised manuscript.

*2) If you have not done so already please begin to revise your manuscript so that it conforms to our Article format instructions, available
here.
Refer also to any guidelines provided in this letter.

*3) Include a revised version of any required Reporting Summary: https://www.nature.com/documents/nr-reporting-summary.pdf
It will be available to referees (and, potentially, statisticians) to aid in their evaluation if the manuscript goes back for peer review.
A revised checklist is essential for re-review of the paper.

Please be aware of our guidelines on digital image standards.

EXTENDED DATA FIGURES

When re-submitting your manuscript, please ensure that any supplementary figures and tables that are crucial to the manuscript's conclusions are converted into Extended Data figures and tables to increase visibility of these data. Extended Data figures and tables are online-only (present in the online PDF and full-text HTML versions of the paper), peer-reviewed display items that provide essential background to the article but are not included in the main article due to space constraints. A maximum of ten Extended Data display items (figures and tables) is permitted.

Please use the link below to submit your revised manuscript and related files:

Link Redacted

Note: This URL links to your confidential home page and associated information about manuscripts you may have submitted, or that you are reviewing for us. If you wish to forward this email to co-authors, please delete the link to your homepage.

We hope to receive your revised manuscript within four to eight weeks. If you cannot send it within this time, please let us know.

Please do not hesitate to contact me if you have any questions or would like to discuss these revisions further.

Nature Genetics is committed to improving transparency in authorship. As part of our efforts in this direction, we are now requesting that all authors identified as 'corresponding author' on published papers create and link their Open Researcher and Contributor Identifier (ORCID) with their account on the Manuscript Tracking System (MTS), prior to acceptance. ORCID helps the scientific community achieve unambiguous attribution of all scholarly contributions. You can create and link your ORCID from the home page of the MTS by clicking on 'Modify my Springer Nature account'. For more information please visit please visit www.springernature.com/orcid.

We look forward to seeing the revised manuscript and thank you for the opportunity to review your work.

Sincerely,

Safia Danovi, PhD
Senior Editor, Nature Genetics
ORCiD: 0009-0007-7822-5479

Reviewers' Comments:

Reviewer #1 (Remarks to the Author):

Authors have followed my suggestions by incorporating new models of inflammation-associated and spontaneous CRC in their comparisons. This work indeed represents foundational datasets and a systematic characterization of a disease process.

Reviewer #2 (Remarks to the Author):

The authors addressed my concerns. In particular the addition of the additional spatial data and the human data improved this manuscript including the translational insights considerably . I have no further comments.

Reviewer #3 (Remarks to the Author):

The manuscript has improved substantially in its revised form. Nevertheless, significant work remains before the study can meet the standards required for publication in Nature Genetics.

At present, the manuscript does not fully support the stated aim of "providing a unique resource and revealing biological insights that were not previously accessible." Much of the analysis appears confirmatory rather than conceptually or technically advancing the field. While validation of known biology is appropriate, the framing continues to overstate the level of novelty relative to what is demonstrated.

My primary concern remains the identification and interpretation of cellular neighborhoods. The spatial transcriptomics (ST) analysis, as currently presented, is not sufficiently rigorous to justify the strength of the claims. Several conclusions extend beyond what the resolution and analytical framework can robustly support.

Below, I outline specific issues in the order they appear in the manuscript:

Line 197 – Spatially contiguous neighborhoods.
The statement that “most clusters formed spatially contiguous, cellular neighbourhoods” does not appear novel. With barcoded spot–based spatial transcriptomics, spatial contiguity is largely expected due to tissue architecture and local mixing of cell types. It is unclear what additional biological insight this observation provides. If the authors intend to convey more than a technical consequence of the method, this should be clearly articulated and supported.

Differentially expressed genes (DEGs).
DEGs are not shown in the main figures and are reportedly provided only in Table 1, which is not accessible. Throughout the manuscript, similarities and differences between clusters are described without clear visual or quantitative support. This limits interpretability and makes it difficult to assess the robustness of the cluster annotations.

scRNA-seq reference dataset.
The reference dataset consists of only three healthy mouse colon samples. This is a limited basis for annotation and may not capture the full diversity of relevant cell states. As a result, important populations may be absent, directly affecting annotation accuracy.
In Figure 2E, cell type proportions are presented based on gene expression, yet the underlying marker genes are not clearly shown. Cluster 10 does not convincingly correspond to a defined cell type. It is unclear whether tumor annotation is based solely on β-catenin together with KRT7 and KRT20 expression (Line 219). This is insufficient to justify definitive tumor classification without additional supporting evidence.

Distribution across sections.
The manuscript states that the first clusters cover most of the tissue; however, 16 sections were analyzed. The distribution of clusters across sections is not described. It remains unclear whether specific clusters are enriched in tumor regions, follicles, or other structures. Without spatial stratification across sections, the biological interpretation of the clustering remains incomplete.

Figure 2G – Heatmap legend.
The meaning of the grey color in the heatmap is not defined and should be clarified.

Figure 2I – Cluster proportions and ordering.
Cluster 1 appears to have similar proportions in Muc2^het and Muc2^hom samples, yet the text (Line 258) states that it is increased. This discrepancy requires correction.
In addition, cluster ordering throughout the manuscript is inconsistent and difficult to follow. While numerical ordering (0, 1, 11, 12, etc.) may reflect bioinformatic conventions, it is not reader-friendly. Reordering clusters across figures further reduces clarity.

Cluster annotation clarity.
Clusters are compared without consistent annotation, making interpretation unnecessarily challenging. For example, in Line 254 the authors refer to an “intermediate phenotype” between clusters 0 and 4, yet these clusters are not clearly defined at that point in the text. They are described as epithelial-enriched, but clusters 12, 13, and 15 are not adequately contextualized in Figure 2E. A systematic and consistent labeling strategy is required throughout.

Arid1a.
Arid1a continues to appear isolated within the analysis and is not clearly integrated into the broader biological narrative.

Overall, the spatial transcriptomics analysis remains the most concerning component of the study. The conclusions drawn from these data are stronger than what the analysis convincingly supports. The manuscript would benefit from more rigorous validation, clearer and consistent annotation, improved visualization of supporting data—particularly DEGs—and a more restrained interpretation of novelty.

Reviewer #4 (Remarks to the Author):

The authors have done a very robust job answering my questions . They have added an additional cac model enu/dss that adds value to the paper and answered all my other concerns .
Overall I believe this a very important study for the literature in colitis associated cancer.

Version 3:

Decision Letter:

Our ref: NG-A68680R2

10th Apr 2026

Dear Dr Winton,

Thank you for submitting your revised manuscript "Paired mutation calling and spatial transcriptomics identify cellular

neighbourhoods dictating the neoplastic outcome of colitis" (NG-A68680R2). It has now been seen by Reviewer #3 who did not provide any comments for the authors but stated in confidential remarks to the Editors that they supported publication of the paper. I am therefore delighted to say that we'll be happy in principle to publish it in Nature Genetics, pending minor revisions to comply with our editorial and formatting guidelines.

If the current version of your manuscript is in a PDF format, please email us a copy of the file in an editable format (Microsoft Word or LaTex)-- we can not proceed with PDFs at this stage.

We are now performing detailed checks on your paper and will send you a checklist detailing our editorial and formatting requirements soon. Please do not upload the final materials and make any revisions until you receive this additional information from us.

Thank you again for your interest in Nature Genetics Please do not hesitate to contact me if you have any questions.

Sincerely,

Safia Danovi, PhD
Senior Editor, Nature Genetics
ORCiD: 0009-0007-7822-5479

Open Access This Peer Review File is licensed under a Creative Commons Attribution 4.0 International License, which permits use, sharing, adaptation, distribution and reproduction in any medium or format, as long as you give appropriate credit to the original author(s) and the source, provide a link to the Creative Commons license, and indicate if changes were made.
In cases where reviewers are anonymous, credit should be given to 'Anonymous Referee' and the source.
The images or other third party material in this Peer Review File are included in the article’s Creative Commons license, unless indicated otherwise in a credit line to the material. If material is not included in the article’s Creative Commons license and your intended use is not permitted by statutory regulation or exceeds the permitted use, you will need to obtain permission directly from the copyright holder.
To view a copy of this license, visit https://creativecommons.org/licenses/by/4.0/

Reviewer #1 (Remarks to the Author):
During the transition from Inflammatory Bowel Disease to cancer, the mutational profile undergoes a fundamental shift: cells initially acquire mutations in inflammation-related genes, but over time, cancer-driving mutations become dominant. The cellular microenvironment's influence on how these different mutated cell populations establish, survive, and expand—and consequently how this impacts cancer development in colitis—remains poorly understood.
To address this question, authors employed a comprehensive experimental approach combining live cell tracking, computational modeling, mutation analysis, and spatial gene expression mapping in a mouse model of inflammation-induced colon cancer. This allowed them to monitor the fate of mutated cell clones throughout chronic inflammation. Their analysis revealed distinct tissue neighborhoods enriched with either epithelial cells or immune cells. They propose that when tissues shift toward a repair-focused state, this microenvironment inadvertently promotes cancer initiation. Specifically, reparative conditions favor the survival and growth of cells carrying cancer-promoting mutations while simultaneously reducing the presence of inflammation-resistant tissue areas that would otherwise harbor non-cancerous mutated cells.
These findings suggest that the tissue's attempt to heal from chronic inflammation creates conditions that accelerate the selection of cancer-driving mutations, providing new insight into how inflammatory diseases progress to malignancy.

Response
We thank the reviewer for their expert assessment of our study.

To broaden the conclusions, I think the manuscript will benefit greatly from including another more common model of CAC, like the AOM-DSS model, that has a well-defined trajectory of inflammatory changes followed by tumor development.
Furthermore, the majority of colorectal cancer is not associated with IBD in humans. However, 90% of CRC is associated to some extent with infiltration of immune cells. Therefore, comparing the expression and mutation neighborhoods that develop in the scenario where tumorigenesis precedes inflammation, like the CPC-APC model, will be very interesting.

Response
Thank you for these suggestions. In the revised manuscript, we address both points by including additional data from two new experimental cohorts:

1. To assess the relevance of our findings in a more classical, chemical-driven inflammation model as the reviewer suggests, whilst keeping in line with the mutagenic insult used in the study, we treated mice with the chemical mutagen ENU followed by DSS, reproducing the classical AOM+DSS model in which mice are first injected with AOM, followed by round of DSS. Mice were collected at two timepoints: 7 days after the end of DSS treatment (n=3 mice) and 26 days after DSS treatment (n=3 mice). At D7, the tissue was largely ulcerated but no tumours could be found. At D26, ulcerated tissue was being repaired, and several tumours could be detected. FFPE colon tissue underwent spatial transcriptomic analysis using the Visium platform.

2. To examine similarities and differences in the case where inflammation precedes or follows tumorigenesis (inflammation-driven vs sporadic tumorigenesis), we collected colon tissue from n=4 mice which were treated with ENU 30 days before the deletion of one copy of the *Apc* gene (*Apc^{het}* model). All mice developed large colonic tumours that are morphologically akin to sporadic colon cancer.

Results from both new experimental cohorts and a comparison of these with the original *Muc2^{hom}* + ENU cohort were included to the manuscript in a paragraph entitled "Exploring cellular neighbourhoods in additional tumourigenesis models", from line 307 to 354 and in Supplementary Figure 5. We found a high degree of similarity between the distributions and representations of cellular neighbourhoods in colitis chemically induced using DSS versus spontaneous colitis in the *Muc2*^{KO} model. In both cases, tumours were spatially close to areas corresponding to the tissue repair cluster. On the other hand, sporadic tumours arising in the *Apc^{het}* model showed a distinct stromal composition from those in the colitis models, with the expansion of a neighbourhood rich in cancer-associated fibroblasts. This serves to highlight context-specific stromal adaptations to tumorigenesis, and we thank the reviewer for motivating these new results.

Could such a scenario explain the accumulation of Arid1a mutations in human CRC? And will similar immune-exclusion zones, IL-17RC mutations or neutrophil-cluster association with tumors be detected? I think these studies will potentially answer the fundamental questions of tumors arising because of aberrant, chronic inflammation or the tumor itself subverting the inflammatory process.

Response

Thank you for this question. In response, please also see the response to Reviewer #2's point 6 regarding the accumulation of inactivating Arid1a mutations in humans. In the revised manuscript, we have now included human immunostaining data showing Arid1a upregulation in colitis-associated tumours and Arid1a outside of tumours, respectively (now added as Fig. 4l, m and text lines 417-424). While we cannot comment on the accumulation of Arid1a in sporadic CRC here, we postulate such Arid1a-depeleted clones potentially form a protective role against CAC, again highlighting a key difference between both types of cancers.

Reviewer #2 (Remarks to the Author):

Moutin and colleagues have employed a genetically engineered confetti mouse model, focusing on the loss of Mucin 2 (Muc2), to investigate clonal expansion in colitis. They further incorporate the chemical mutagen ENU to simulate colitis-associated tumorigenesis. Through robust molecular characterization, utilizing spatial transcriptomics and genetic profiling, the authors propose that specific cellular neighborhoods, indicative of reparative tissue environments, play an important role in facilitating tumor initiation. The manuscript is well-written and advances our understanding of neoplastic progression in the context of inflammation.

Response

Thank you for the very positive evaluation of our work.

However, several issues need addressing or further clarification based on the data provided.

1. The authors discuss the DSS-induced colitis model and the emergence of a fetal-like state. They note similar enrichment patterns in their Muc2het vs. Muc2ko model using GSEA (Figure 1G). The Trop2 IF (Figure 1H) suggests these fetal-like cells are mutually exclusive concerning their labeled clones. Could the authors elaborate on this observation?

Response

Thank you for this request. Labelled clones expand in Trop2-positive areas, and whilst these clones are not usually 100% Trop2-positive, some cells do express Trop2. To highlight this, we added two inserts in Fig.1h, zooming in on a clone in a Trop2-negative and a Trop2-positive area, where yellow staining indicates overlap between Trop2 (red) and GFP labelling clones (green). We hope this adds clarification to the observation.

2. The authors utilize Visium to explore cellular neighborhoods within the Muc2ko model. However, the dataset presented includes only slides from the Muc2ko model. Incorporating Muc2het and/or WT samples would allow for comparisons against a non-colitis background. Is neighborhood Cluster 4 unique to the Muc2ko model compared to Muc2het or WT?

Response

The reviewer makes an important point regarding the exclusivity of clusters identified in *Muc2^hom^* mice to the setting of chronic inflammation. In the revised manuscript, Visium was applied to generate spatial transcriptomics data using colonic tissue from a new cohort of three *Muc2^het^* mice. Cluster labels generated for the *Muc2*^hom^ + ENU dataset were transferred to the additional samples to identify the representation of cellular neighbourhoods in non-inflamed mice. Most barcodes from *Muc2*^het^ samples were found to map to clusters 1, 2 and 3, validating the assignment of these clusters as healthy epithelium. Conversely, *Muc2^het^* samples displayed fewer barcodes mapping to the repair cluster 4 and immune-refractory cluster 0, which were largely associated with the *Muc2^hom^* genotype. In further support of this point, we also included Visium datasets with another new cohort of three *Muc2^hom^* mice (without ENU treatment), and found larger proportion of spatial barcodes mapping to clusters 4 and 0 in these untreated *Muc2^hom^* samples (see also the same reviewer's point 3 below), reflecting their inflamed state (Figure 2i, Supplementary Fig. 4b), added to the text lines 250-257. We thank the reviewer for motivating these new results, which provide additional support to the claim that clusters 0 and 4 are colitis-associated clusters.

3. Presumably, the reparative Cluster 4 is present in Muc2ko tissues before ENU treatment. What, if any, are the differences between the Trop2 positive neighborhoods identified in Figure 1H, and the neighborhoods of Cluster 4 described in Figure 2? Profiling Muc2ko samples without ENU treatment could help to resolve this.

Response

As Trop2 is expressed in *Muc2*^{hom} mice that have not been treated with ENU and is also a top marker gene of cluster 4, the assumption of the reviewer is valid. As described in the response to the reviewer's previous point above, to assess whether cluster 4 is present in the context of chronic inflammation in the absence of treatment with the chemical mutagen, we performed spatial transcriptomics on colonic samples from a new cohort of n=3 *Muc2*^{hom} mice (not ENU-treated) using Visium. Transfer of cluster labels revealed cluster 4 is indeed present in *Muc2*^{hom} samples (Figure 2i, Supplementary Fig. 4b), added to the text lines 250-257.

4. In Figure 4B, the authors identify numerous tumor-associated mutations within clusters 4 and 10, suggesting a mechanistic link explaining tumor initiation in repairing epithelium during chronic inflammation. However, the system's dynamics remain unclear. Is cluster 4 a precursor to cluster 10? Does this transition require additional genetic alterations, or does it indicate differing stages of tumorigenesis? Analyzing tissues across multiple time points might help clarify this matter.

Response

The reviewer makes a great point regarding the dynamics of lineage relationships between clusters, specifically the repair cluster 4 and tumour cluster 10. Visium data from multiple timepoints would answer this question if inflammation in the *Muc2*^{KO} model was increasing in a linear, tractable fashion. As this is not the case, we instead employed SpaceFlow, a graph-based deep-learning method, to generate a pseudo-spatiotemporal ordering of clusters based on gene expression and spatial proximity (Fig. 3f,g text lines 281-305). Since integration of multiple samples is not supported by the SpaceFlow workflow, the analysis was applied to the sample with the highest tumour burden to identify pseudo-spatiotemporal proximity of tumour and surrounding epithelial-rich clusters. As now shown in the revised Fig. 3, this analysis revealed that the repair cluster 4 is spatiotemporally closest to the tumour cluster 10, which could possibly be interpreted as a direct lineage relationship between both clusters. However, we are cautious to point out that spatiotemporal proximity alone is not a direct indicator of *genotypic* lineage relationships and are therefore unable to make this claim in the revised manuscript. Instead, identification of common mutations mapping to spatial regions containing barcodes from both clusters 4 and 10 as the reviewer points out could simply be a consequence of the strong symmetric balance correlations between these two cellular neighbourhoods (viz. Fig. 3b). To elucidate *phenotypic* lineage relationships between the major non-tumour cellular neighbourhoods, we used Slingshot to infer the topology of trajectories between clusters and interpreted these in the context of the pseudo-spatiotemporal ordering predicted by SpaceFlow. This extended analysis identified two distinct trajectories originating at the healthy cluster 3, one leading to the tumour-associated repair lineage and one leading to the immune-refractory lineage (Fig. 3h, text lines 281-305). We thank the reviewer for motivating this detailed lineage analysis.

5. What factors do the authors suggest could render reparative niches more prone to acquiring oncogenic mutations?

Response

Interestingly, both the tissue repair cluster 4 and the neutrophil-rich cluster 11 correlate with increasing tumour burden (Fig. 3b), and the marker of tissue repair Trop2 is up-regulated in cluster 11 (log2 fold change 3.52, p value $<1.5e^{-300}$). Neutrophils are known to contribute to oxidative stress and genomic instability (Coussens & Werb., *Nature*, 2002; Putoczki et al., *Cancer Cell* 2013). To test whether neutrophils could promote tumourigenesis within cluster 4 cells, we looked at spatial proximity between spots mapped to each cluster using the Giotto suite. We found repair cluster 4 to be the only epithelial-rich cluster with positive spatial correlation with the neutrophil-rich cluster 11 (Fig. 3e). Furthermore, looking at markers of cluster 4 we identified many neutrophil attracting factors (*Cxcl3*, *Cxcl5*, *S100a8*, *S100a9* and *Ccl5*), suggesting possible recruitment of neutrophils. This is aligned with an interpretation where the repairing epithelium recruits neutrophils, contributing to genomic instability leading to the acquisition of oncogenic mutations in this niche. This was added in results (lines 264-280) and discussion (lines 440-445).

6. Further incorporation of human samples to corroborate the key findings would significantly lift the impact of the study.

Response

We thank the reviewer for the opportunity to lift the impact of our study. We obtained samples from IBD patients spanning different severity, from mild inflammation to moderate, severe, high-grade dysplasia and cancer. Immunostaining for Arid1a showed upregulation in tumours, and we could detect Arid1a-depleted clones away from tumours as seen in the *Muc2*^{hom}+ENU model of CAC (Fig. 4l, m), indicating that analogous epithelial neighbourhoods are present in human IBD and CAC, underscoring the translational relevance of this study (text lines 417-424).

7. Is the expression module of Cluster 4 prognostic for the development of colitis associated cancer in humans?

Response

This question is extremely relevant to clinical application, and we would love to be able to answer it. Whilst we can use previously published data to comment on the survival of colon cancer patients with tumours enriched for specific markers (Trop2: Svec et al., 2022), to determine the prognostic value of such marker would require expression data from a much more defined cohort of patients. Specifically, we would need to analyse the expression level of the cluster 4 signature in IBD patients before cancer diagnostic and relate the expression of the signature to the time of the cancer diagnostic. No such data is currently publicly available, and conducting such a study is outside the scope for this article but we thank the reviewer for a suggestion that could perhaps be addressed in future work.

Minor Points:

1. In Figure 1G, the P-value is stated as '0.000'. This seems to be a rounding issue and should be expressed in scientific notation to convey the precise value.

Response
We apologize for the standardisation, which is due to the use of the GSEA software. A p value of zero (0.0) indicates an actual p value of less than 1/number-of-permutations. As 1000 permutations were used, the p value was lower than 0.001. For more accuracy, the analysis was run again using the GSEA R package, and a p value of 2.2e-7 was found. This value was indicated on the updated GSEA plot in Fig. 1J.

2. In Figure 2E, several paired GSEA comparisons are presented. Given the intention to compare multiple groups, the authors might consider employing GSVA for this analysis.

Response
Thank you for this suggestion. In the revised manuscript and corresponding analysis and figure, GSEA was replaced by GSVA, more suitable to the comparison of enrichment of specific pathways between clusters (Fig. 2g, Supplementary Table 2, text lines 230-250).

3. For Figure 2, a deconvolution analysis using scRNAseq as a reference could enhance the interpretation of cellular neighborhoods.

Response
We thank the reviewer for this suggestion. In the revised manuscript, the Dampened Weighted Least Squares (DWLS) deconvolution algorithm was used to obtain cell type proportion for each cluster, based on major cell type signatures derived from a single-cell dataset with mouse colon in various stages of inflammation (Accession GSE264408). This new approach was incredibly useful for confirming and refining our definition of clusters. The corresponding figure is now Fig. 2d and the discussion of results has now been added the main text (text lines 219-226):

Reviewer #3 (Remarks to the Author):

While the topic is highly relevant and the authors employ a broad range of methods, the current manuscript lacks the mechanistic rigor, analytical depth, and conceptual innovation expected for publication in Nature Genetics. The findings remain largely descriptive, speculative, and insufficiently validated. For these reasons, I do not recommend the manuscript for publication.

Reviewer #4 (Remarks to the Author):
The manuscript "Paired mutation calling and spatial transcriptomics identify cellular neighbourhoods dictating the neoplastic outcome of colitis" assesses the mutational and transcriptional landscape of tissue progressing from IBD tissue to IBD-associated cancer. The work and investigative/analytical approach adopted in this manuscript are both interesting and very worthy of publication. Whilst many of the key findings are not entirely novel (e.g. inflammation promotes expansion of pro-tumorigenic clones, the association between inflammation duration and clonal expansion), the study provides robust evidence supporting the clonal dynamics underlying the previously established molecular pathogenesis of colitis-associated colorectal cancer (CRC). This contributes valuable confirmation and clarity to the existing body of knowledge.

Overall manuscript comments
Overall, the manuscript is well-written and easy to follow. However, the final sections discussing the analysis of the mutational landscape are somewhat more complex and could benefit from simplification to improve readability and comprehension.

Response
We thank the reviewer for their positive and constructive comments on our work.

Specific comments
Figure 1a, 1b and 1c / Lines 104-112
Could the author explain the reason behind their choice to use Muc2het mice as controls instead of Muc2Wt. Do Muc2het mice get any inflammation? If yes, would a Muc2wt mouse be a better baseline control? Moreover, could the comparison between Muc2het and Muc2hom used to infer on the relationship between inflammation extent and clonal expansion (as we already know that in humans more severe inflammation is associated with great CRC risk)?

Response
We hope the following answers this question appropriately. For added clarity on this matter, in the revised manuscript a panel was added to Supplementary Figure 1 illustrating mucin production in $Muc2^{wt}$, $Muc2^{het}$ and $Muc2^{hom}$ small bowel and colon, showing reduction in mucin production only in the $Muc2^{hom}$ colon. $Muc2^{wt}$ and $Muc2^{het}$ small bowels and colons are alike, and in accordance we never saw any clinical signs of inflammation in $Muc2^{het}$ mice, and their survival is similar to $Muc2^{wt}$ mice (Moutin *et al.*, 2023). $Muc2^{het}$ mice can be bred and maintained in the same cages as $Muc2^{hom}$ mice in an efficient breeding strategy, which explains our decisions to use them as controls.

Figure 1e and 1f:
The labelling and colouring used in Figures 1E and 1F is slightly confusing. My understanding is that the grey colour represents the undamaged grid, which also corresponds to the grid under homeostatic conditions. In Figure 1F (left), the grey line appears to represent both the undamaged grid from Figure 1E and the grid prior to the application of damage. If this is correct, then in Figure 1F (right), the grey line should correspond to the undamaged/homeostatic condition, and the red line should represent the damaged/regenerating condition. Could you please clarify if this interpretation is accurate?
• Could the authors consider making the figure legends for Figures 1E and 1F slightly more detailed to better guide the reader through the data presentation? A similar suggestion applies to Figures 1K and 1L, where additional explanation would help improve clarity and aid interpretation.

Response
We apologise for the confusion regarding the interpretation of our *in silico* simulations, which was caused in part by our previous presentation of the corresponding figure panels. In the revised Fig. 1 d-h, we have reorganised the illustration of key results by presenting both fission neutral (upper) and fission bias (lower) scenarios in direct

alignment to help guide the reader's eye. We have additionally simplified the colour code to enable a more intuitive mapping between the quantities of interest. In panels Fig. 1 d,g we now show simulation snapshots with undamaged (left) and damaged (right) sides of the grid bordered in grey and red, respectively. Next, in panels Fig. 1 e,h we now plot the total number of empty (black), occupied labelled (green) and occupied unlabelled (blue) sites averaged across 1000 simulations, the important point here being that the undamaged side (left, grey border) attains a homeostatic state to which the damaged side (right, red border) returns to post-repair. Finally, in panels Fig. 1f,i we now plot the aggregation of sites for both undamaged (grey line) and damaged (red line) on the same axis to display the elevated aggregation in the damaged region following repair. The corresponding text and figure legend has been modified to accommodate these improved figures, and we hope the interpretation of key results is now much more apparent to the reviewer and general reader.

• If my understanding of Figure 1F (right) is correct, the persistence of the damaged grid/line is expected and illustrates that, following an initial increase in site aggregation, this begins to decline over time. If the simulation was extended further, would the level of aggregation eventually plateau at the same level as the undamaged grid, or would it stabilise at a new, elevated homeostatic state? (hinting at a form of long-term memory following damage)

Response
We thank the reviewer for this remark and raising the fascinating question of whether our simulations can address the role of clone aggregation as a form of long-term tissue memory. To investigate the point raised regarding the asymptotic behaviour of the aggregation metric, we ran simulations for many more simulation time steps (1 million, up from 200,000) and provide a zoomed-in comparison of site aggregation at late time on both the left- (undamaged) and right-hand (damaged) sides of the grid-- see Figure below. At this level of resolution, it appears that the level of aggregation does indeed plateau at a higher value on the right-hand grid, potentially suggesting some lasting form of memory following damage. However, we are quick to highlight that this observation could be impacted by imperfect averaging over a finite number (1000) of simulations which, while sufficient for capturing the larger trends over shorter timeframes, may be insufficient for capturing the subtle, asymptotic features of aggregation. Analysis of the asymptotic properties of our model instead requires a rigorous mathematical treatment of the stochastic process, which will be the focus of future work.

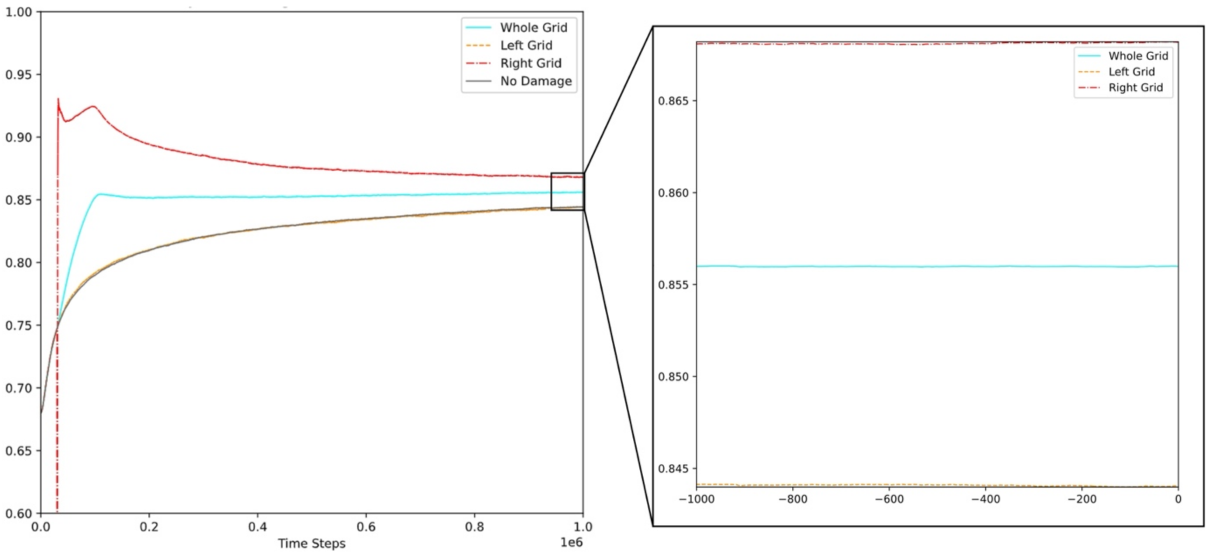

• Typo in line 131

Response
The sentence was rephrased and typo corrected.

Supplementary Figure 1:
• Statistical analysis appears to be missing from the survival plot.

Response
P values for log-rank test were added to the Kaplan-Meiri survival plot in Supp. Fig. 1D.

• What does the Muc2het small bowel look like?

Response
The *Muc2^het^* small bowel does show any sign of inflammation, just as the *Muc2^hom^* small bowel. To illustrate this, a panel was added to Supplementary Figure 1 showing Alcian Blue/Pas staining of mucins in *Muc2^wt^*, *Muc2^het^* and *Muc2^hom^* mice small bowel and colon. This shows a reduction in the production of mucins in the *Muc2^hom^* colon specifically, whilst *Muc2^wt^* and *Muc2^het^* small bowel and colon are alike.

Lines 139-144:
• While the findings are certainly interesting, the conclusions regarding increased crypt fission are not particularly novel, as increased crypt fission has been previously reported in the context of both regeneration and IBD. Nonetheless, the authors present elegant proof of concept experiments that reinforce and illustrate these observations effectively.

Response
Thank you for these positive comments.

Lines 154-157:
• The authors state “This highlights how tissue repair conditions can accelerate and promote the accumulation and expansion of mutant clones in damaged tissue.” in

relation to their in-silico observation of fission-biased clones. Would it be more correct to state that the data highlights how tissue repair conditions can accelerate and promote the accumulation and expansion of clones with a proliferative (or fission biased) advantage rather mutant clones in general.

Response
We thank the reviewer for spotting this generalisation. Text lines 154-157 were updated to: "This highlights how tissue repair conditions can accelerate and promote the accumulation and expansion of fission-biased clones in damaged tissue."

Lines 169-172
• Should these lines be merged with the next paragraph?. Identification of cellular neighbourhoods in colitis-associated neoplasia/biological processes defining epithelial neighbourhoods

Response
Text lines 169-172 were included into the "identification of cellular neighbourhoods in colitis-associated neoplasia" paragraph.

• The authors should be commended for their elegant experimental design, robust analytical approach, and their ability to extract key insights into clonal dynamics and cellular neighbourhoods from spatial transcriptomics data.

Response
We thank the reviewer for this very positive comment.

Line 205
• Typo in neighbourhood

Response
The typo was corrected. Text line 228: "Biological processes defining epithelial neighbourhoods"

Lines 188-200
• The authors report: high, low, abundant... expression of the different keratins. How is high/low being defined? Is there a numerical/statical value that is being used as cut off. As for example, visually cluster 11 also appears to be enriched for keratin 7 and keratin 14

Response
Bar plots showing the percentage of cells expressing each of the keratins presented in each cluster were added to Supp. Fig3e. This shows a high expression of Krt7 and 14 in cluster 11 (neutrophil-rich cluster) as the reviewer highlighted. This is in line with the spatial correlation we observed between cluster 11 and the tumour cluster 10 and adds to the evidence that neutrophils infiltrating epithelial neighbourhoods may be participating in the promotion of a tumorigenic environment.

Figure 2g / Lines 237-242:

• The association between regenerative epithelium and tumorigenic tissue is not a novel finding, especially in the context of IBD-CRC, as previously reported by Gil Vazquez et al., 2022. Nevertheless, the current study provides valuable proof of this relationship at the cellular level.

Response

Thank you for pointing this out. We added a sentence to the discussion, mentioning the specific reference suggested by the reviewer, as well as Levi-Galibov et al., 2020: "Tissue repair and the associated remodelling of the extracellular matrix is commonly viewed as cancer promoting[45,46]." (Lines 437-438).

Figure 3 / Lines 268-289

• The mutational landscape detected in the Muc2hom + ENU mice has some clear differences from that reported in human IBD-CRC (e.g. Apc highly mutated in the mice, but reported to be mutated only in 10% of IBD-CRC, p53 highly mutated in IBD-CRC (>70%) but lowly mutated in the mice). Can the authors comment on this. While the overall message with regards to association of specific mutational signatures with specific clusters remains intact, from a mutational point of view is this perhaps not the best model of IBD-CRC? Does the Muc2hom + ENU model at the genetic level represent more of a model of sporadic CRC with an inflammatory push.

Response

The reviewer is correct, tumourigenesis in our model is Wnt-driven, which is not commonly the case for IBD-related colon cancer. Unfortunately, this is also the case in the more established AOM+DSS model which is also Wnt-driven (Chatila et al., 2023). Importantly, using the Muc2hom+ENU model we were able to detect some mutations present in human IBD-related colon cancer, such as Smad4 mutations, as well as mutations present in human IBD: Arid1a, Nfkbiz, Il17rc etc.

• Having said that the findings on Arid1a are compelling and do fit well with the high mutational burden of the gene in human IBD-CRC.

Response

Thank you for this positive comment.

Supplementary Figure 6

• In terms of the tumour associated genes and their distribution: are different Wnt mutations were mutually exclusive?

Response

The co-occurrence of tumour-associated *Ctnnb1* and *Apc* mutations was assessed across cores. Most cores did not carry either mutation (154 cores). Among mutated cores, 20% carried both mutations, at face value giving the impression that *Ctnnb1* and *Apc* mutations are largely mutually exclusive. However, Fisher's exact test showed no

statistically significant association between the two mutations ($p = 0.31$), indicating that the observed co-occurrence could arise by chance.

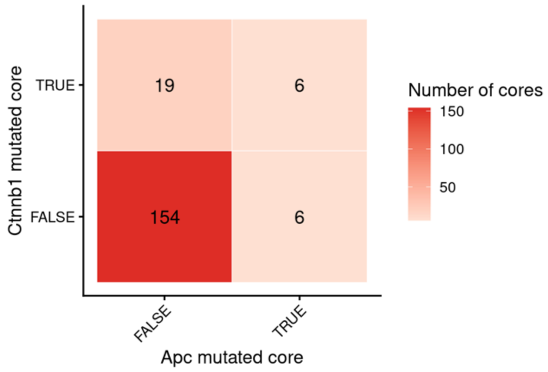

• Can the association between specific mutations and specific cluster be used to infer a temporal acquisition of the mutations?

Response

We thank the reviewer for this interesting suggestion. We looked at the distribution of Ctnnb1 mutations identified as tumour-associated in tumour vs non-tumour cores. T41I, S37P, I35S and D32G were found in both tumour and non-tumour cores, suggesting they may be acquired earlier than the other Ctnnb1 mutations profiled.

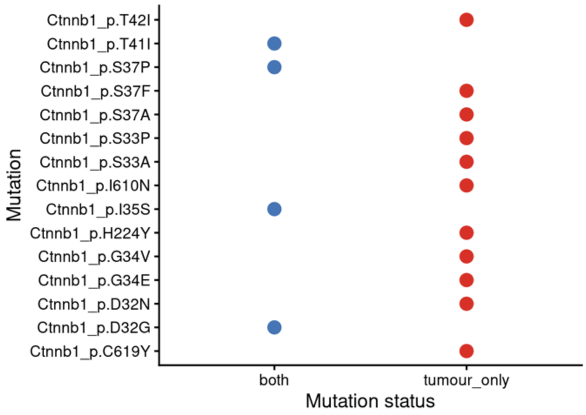

Minor Comment:

The introduction is well written and provides a solid background for the manuscript. However, it would benefit from referencing key publications that have previously examined the mutational landscape of colitis-associated colorectal cancer, such as Baker AM, Cross W, Curtius K, et al. Gut, 2019 Including these studies would help contextualise the current work within the existing literature.

Response

Lines 78-80: The introduction was re-worked to include seminal work that characterised the mutational landscape of colitis associated cancer: "Extensive characterisation of the mutational landscape of CAC has revealed a reduced prevalence of inflammation-resistance mutations in tumours arising in the context of IBD[5–9], hinting at a protective role against cancer."

Where references 5-9 are:

5. Chatila, W. K. *et al*. Integrated clinical and genomic analysis identifies driver events and molecular evolution of colitis-associated cancers. *Nat Commun* **14**, 1–13 (2023).
6. Baker, A. M. *et al*. Evolutionary history of human colitis-associated colorectal cancer. *Gut* **68**, 985–995 (2019).
7. Yin, J. *et al*. *P53 Point Mutations in Dysplastic and Cancerous Ulcerative Colitis Lesions*. *Gastroenterology,* **104,** 1633-1639 (1993).
8. Galandiuk, S. *et al*. Field cancerization in the intestinal epithelium of patients with Crohn's ileocolitis. *Gastroenterology* **142**, 855–864 (2012).
9. Brentnall, T. A. *et al*. Mutations in the p53 gene: An early marker of neoplastic progression in ulcerative colitis. *Gastroenterology* **107**, 369–378 (1994).

Reviewer #4 (Remarks on figshare data availability):
The data looks robust and agreement with the claims in the paper

Response
Thank you for this positive comment.

Reviewer #1 (Remarks to the Author):

During the transition from Inflammatory Bowel Disease to cancer, the mutational profile undergoes a fundamental shift: cells initially acquire mutations in inflammation-related genes, but over time, cancer-driving mutations become dominant. The cellular microenvironment's influence on how these different mutated cell populations establish, survive, and expand—and consequently how this impacts cancer development in colitis—remains poorly understood.

To address this question, authors employed a comprehensive experimental approach combining live cell tracking, computational modeling, mutation analysis, and spatial gene expression mapping in a mouse model of inflammation-induced colon cancer. This allowed them to monitor the fate of mutated cell clones throughout chronic inflammation. Their analysis revealed distinct tissue neighborhoods enriched with either epithelial cells or immune cells. They propose that when tissues shift toward a repair-focused state, this microenvironment inadvertently promotes cancer initiation. Specifically, reparative conditions favor the survival and growth of cells carrying cancer-promoting mutations while simultaneously reducing the presence of inflammation-resistant tissue areas that would otherwise harbor non-cancerous mutated cells.

These findings suggest that the tissue's attempt to heal from chronic inflammation creates conditions that accelerate the selection of cancer-driving mutations, providing new insight into how inflammatory diseases progress to malignancy.

Response

We thank the reviewer for their expert assessment of our study.

To broaden the conclusions, I think the manuscript will benefit greatly from including another more common model of CAC, like the AOM-DSS model, that has a well-defined trajectory of inflammatory changes followed by tumor development.

Furthermore, the majority of colorectal cancer is not associated with IBD in humans. However, 90% of CRC is associated to some extent with infiltration of immune cells. Therefore, comparing the expression and mutation neighborhoods that develop in the scenario where tumorigenesis precedes inflammation, like the CPC-APC model, will be very interesting.

Response

Thank you for these suggestions. In the revised manuscript, we address both points by including additional data from two new experimental cohorts:

1. To assess the relevance of our findings in a more classical, chemical-driven inflammation model as the reviewer suggests, whilst keeping in line with the mutagenic insult used in the study, we treated mice with the chemical mutagen ENU followed by DSS, reproducing the classical AOM+DSS model in which mice are first injected with AOM, followed by rounds of DSS. Mice were collected at two timepoints: 7 days after the end of DSS treatment (n=3 mice) and 26 days after DSS treatment (n=3 mice). At D7, the tissue was largely ulcerated but no tumours could be found. At D26, ulcerated tissue was being repaired, and several tumours could be detected. FFPE colon tissue underwent spatial transcriptomic analysis using the Visium platform.

2. To examine similarities and differences in the case where inflammation precedes or follows tumorigenesis (inflammation-driven vs sporadic tumorigenesis), we collected colon tissue from n=4 mice which were treated with ENU 30 days before the deletion of one copy of the *Apc* gene (*Apc^het^* model). All mice developed large colonic tumours that are morphologically akin to sporadic colon cancer.

Results from both new experimental cohorts and a comparison of these with the original *Muc2^hom^* + ENU cohort were included to the manuscript in a paragraph entitled "Exploring cellular neighbourhoods in additional tumourigenesis models", from line 312 to 355 and in Supplementary Figure 5. We found a high degree of similarity between the distributions and representations of cellular neighbourhoods in colitis whether chemically induced using DSS or arising spontaneously in the *Muc2*^KO^ model. In both cases, tumours were spatially close to areas corresponding to the tissue repair cluster.

In contrast, sporadic tumours arising in the *Apc^het^* + ENU model showed a distinct stromal composition from those in the colitis models, with the expansion of a neighbourhood rich in cancer-associated fibroblasts. This serves to highlight context-specific stromal adaptations to tumorigenesis, and we thank the reviewer for motivating these new results.

Could such a scenario explain the accumulation of Arid1a mutations in human CRC? And will similar immune-exclusion zones, IL-17RC mutations or neutrophil-cluster association with tumors be detected? I think these studies will potentially answer the fundamental questions of tumors arising because of aberrant, chronic inflammation or the tumor itself subverting the inflammatory process.

Response
Thank you for this question. The immune refractory cluster 0 is seen in the context of the *Apc^het^* + ENU sporadic colon cancer model, however only within tumours (Figure 2e). We believe this demonstrates a common and context dependent means of achieving a survival advantage by immune suppression. In the context of IBD, this protects the epithelium against oncogenic stress generated by inflammation, whilst immune suppression by tumour cells promotes tumour growth. Regarding the fundamental question the reviewer poses, this is consistent with both the possibilities stated but adds the insight that, in addition, regions of non tumour epithelium can also subvert the inflammatory process to minimise their risk of oncogenic transformation. This is now addressed in the discussion lines 454-460.

Reviewer #2 (Remarks to the Author):
Moutin and colleagues have employed a genetically engineered confetti mouse model, focusing on the loss of Mucin 2 (Muc2), to investigate clonal expansion in colitis. They further incorporate the chemical mutagen ENU to simulate colitis-associated tumorigenesis. Through robust molecular characterization, utilizing spatial transcriptomics and genetic profiling, the authors propose that specific cellular neighborhoods, indicative of reparative tissue environments, play an important role in facilitating tumor initiation. The manuscript is well-written and advances our understanding of neoplastic progression in the context of inflammation.

Response
Thank you for the very positive evaluation of our work.

However, several issues need addressing or further clarification based on the data provided.

1. The authors discuss the DSS-induced colitis model and the emergence of a fetal-like state. They note similar enrichment patterns in their Muc2het vs. Muc2ko model using GSEA (Figure 1G). The Trop2 IF (Figure 1H) suggests these fetal-like cells are mutually exclusive concerning their labeled clones. Could the authors elaborate on this observation?

Response
Thank you for this request. Labelled clones expand in Trop2-positive areas, and whilst these clones are not usually 100% Trop2-positive, some cells do express Trop2. To highlight this, we added two inserts in Fig.1k, zooming in on a clone in a Trop2-negative and a Trop2-positive area, where yellow staining indicates overlap between Trop2 (red) and GFP labelling clones (green). We hope this adds clarification to the observation.

2. The authors utilize Visium to explore cellular neighborhoods within the Muc2ko model. However, the dataset presented includes only slides from the Muc2ko model. Incorporating Muc2het and/or WT samples would allow for comparisons against a non-colitis background. Is neighborhood Cluster 4 unique to the Muc2ko model compared to Muc2het or WT?

Response
The reviewer makes an important point regarding the exclusivity of clusters identified in *Muc2^{hom}* mice to the setting of chronic inflammation. In the revised manuscript, Visium was applied to generate spatial transcriptomics data using colonic tissue from a new cohort of n=3 *Muc2^{het}* mice. Cluster labels generated for the *Muc2*^{hom} + ENU dataset were transferred to these additional samples to identify the representation of cellular neighbourhoods in non-inflamed mice. Most barcodes from *Muc2*^{het} samples were found to map to clusters 1, 2 and 3, validating the assignment of these clusters as healthy epithelium. Conversely, *Muc2^{het}* samples displayed less than half the number of barcodes mapping to the repair cluster 4 and only a few barcodes mapping to the immune-refractory cluster 0, both of which were largely associated with the *Muc2^{hom}* genotype. In further support of this point, we also included Visium datasets with another new cohort of three *Muc2^{hom}* mice (without ENU treatment), and found a large proportion of spatial barcodes mapping to clusters 4 and 0 in these untreated *Muc2^{hom}* samples (see also the same reviewer's point 3 below), reflecting their inflamed state (Figure 2i, Supplementary Fig. 4b), added to the text lines 255-261. We thank the reviewer for motivating these new results, which provide additional support to the claim that clusters 0 and 4 are colitis-associated clusters.

3. Presumably, the reparative Cluster 4 is present in Muc2ko tissues before ENU treatment. What, if any, are the differences between the Trop2 positive neighborhoods identified in Figure 1H, and the neighborhoods of Cluster 4 described in Figure 2? Profiling Muc2ko samples without ENU treatment could help to resolve this.

Response

As Trop2 is expressed in *Muc2*^{hom} mice that have not been treated with ENU and is also a top marker gene of cluster 4, the assumption of the reviewer is valid. As described in the response to the reviewer's previous point above, to assess whether cluster 4 is present in the context of chronic inflammation in the absence of treatment with the chemical mutagen, we performed spatial transcriptomics on colonic samples from a new cohort of n=3 *Muc2*^{hom} mice (not ENU-treated) using Visium. Transfer of cluster labels revealed cluster 4 is indeed present in *Muc2*^{hom} samples (Figure 2i, Supplementary Fig. 4b), added to the text lines 255-261.

4. In Figure 4B, the authors identify numerous tumor-associated mutations within clusters 4 and 10, suggesting a mechanistic link explaining tumor initiation in repairing epithelium during chronic inflammation. However, the system's dynamics remain unclear. Is cluster 4 a precursor to cluster 10? Does this transition require additional genetic alterations, or does it indicate differing stages of tumorigenesis? Analyzing tissues across multiple time points might help clarify this matter.

Response

The reviewer makes a great point regarding the dynamics of lineage relationships between clusters, specifically the repair cluster 4 and tumour cluster 10. Visium data from multiple timepoints would answer this question if inflammation in the *Muc2*^{KO} model was increasing in a linear, tractable fashion. As this is not the case, we instead employed SpaceFlow, a graph-based deep-learning method, to generate a pseudo-spatiotemporal ordering of clusters based on gene expression and spatial proximity (Fig. 3f,g text lines 286-310). Since integration of multiple samples is not supported by the SpaceFlow workflow, the analysis was applied to the sample with the highest tumour burden to identify pseudo-spatiotemporal proximity of tumour and surrounding epithelial-rich clusters. As now shown in the revised Figure 3, this analysis revealed that the repair cluster 4 is spatiotemporally closest to the tumour cluster 10, which could possibly be interpreted as a direct lineage relationship between these two clusters. However, we are cautious to point out that spatiotemporal proximity alone is not a direct indicator of *genotypic* lineage relationships and are therefore unable to make this claim in the revised manuscript. Instead, identification of common mutations mapping to spatial regions containing barcodes from both clusters 4 and 10 could simply be a consequence of the strong correlations of the presence of these two cellular neighbourhoods visualisable in Fig. 3a and b. To elucidate *phenotypic* lineage relationships between the major non-tumour cellular neighbourhoods, we further inferred the topology of trajectories between clusters using Slingshot, a graph-based method that infers lineage relationship from transcriptional similarity, and interpreted these in the context of the pseudo-spatiotemporal ordering predicted by SpaceFlow. This extended analysis identified two distinct trajectories originating at the healthy cluster 3, one leading to the tumour-associated repair lineage and one leading to the immune-refractory lineage (Fig. 3h, text lines 286-310). We thank the reviewer for motivating this detailed lineage analysis.

5. What factors do the authors suggest could render reparative niches more prone to acquiring oncogenic mutations?

Response

Interestingly, both the tissue repair cluster 4 and the neutrophil-rich cluster 11 correlate with increasing tumour burden (Fig. 3b), and the marker of tissue repair Trop2 is up-regulated in cluster 11 (gene *Tacstd2*, log2 fold change 3.52, p value <1.5e^{-300}). Neutrophils are known to contribute to oxidative stress and genomic instability[1,2]. To test whether neutrophils could promote tumourigenesis within cluster 4 cells, we looked at spatial proximity between spots mapped to each cluster. We found repair cluster 4 to be the only epithelial-rich cluster with positive spatial correlation with the neutrophil-rich cluster 11 (Fig. 3e). Furthermore, looking at markers of cluster 4 we identified many neutrophil attracting factors (*Cxcl3*, *Cxcl5*, *S100a8*, *S100a9* and *Ccl5*), suggesting possible recruitment of neutrophils. This aligns with an interpretation where the repairing epithelium recruits neutrophils, contributing to genomic instability leading to the acquisition of oncogenic mutations in this niche. This interpretation was added in the discussion (lines 441-443).

6. Further incorporation of human samples to corroborate the key findings would significantly lift the impact of the study.

Response
We thank the reviewer for the opportunity to lift the impact of our study. Immunostaining for Arid1a in tissue from colitis-associated-cancer patients showed upregulation in tumours, and we could detect Arid1a-depleted clones away from tumours as seen in the *Muc2*^{hom}+ENU model of CAC (Fig. 4l, m), underscoring the translational relevance of this study (text lines 417-422).

7. Is the expression module of Cluster 4 prognostic for the development of colitis associated cancer in humans?

Response
This question is extremely relevant to clinical application, and we would love to be able to answer it. Whilst we can use previously published data to comment on the survival of colon cancer patients with tumours enriched for specific markers[3], to determine the prognostic value of such marker would require expression data from a much more defined cohort of patients. Specifically, we would need to analyse the expression level of the cluster 4 signature in IBD patients before cancer diagnostic and relate the expression of the signature to the time of the cancer diagnostic. No such data is currently publicly available, and conducting such a study is outside the scope for this article, but we thank the reviewer for a suggestion that could perhaps be addressed in future work.

Minor Points:
1. In Figure 1G, the P-value is stated as '0.000'. This seems to be a rounding issue and should be expressed in scientific notation to convey the precise value.

Response
We apologize for the standardisation, which is due to the use of the GSEA software. A p value of zero (0.0) indicates an actual p value of less than 1/number-of-permutations. As 1000 permutations were used, the p value was lower than 0.001. For more accuracy, the analysis was run again using the GSEA R package, and a p value of 2.2e-7 was found. This value was indicated on the updated GSEA plot in Fig. 1J.

2.In Figure 2E, several paired GSEA comparisons are presented. Given the intention to compare multiple groups, the authors might consider employing GSVA for this analysis.

Response
Thank you for this suggestion. In the revised manuscript and corresponding analysis and figure, GSEA was replaced by GSVA, more suitable to the comparison of enrichment of specific pathways between clusters (Fig. 2g, Supplementary Table 2, text lines 236-255).

3. For Figure 2, a deconvolution analysis using scRNAseq as a reference could enhance the interpretation of cellular neighborhoods.

Response
We thank the reviewer for this suggestion. In the revised manuscript, the Dampened Weighted Least Squares (DWLS) deconvolution algorithm was used to obtain cell type proportion for each cluster, based on major cell type signatures derived from a single-cell dataset with mouse colon in various stages of inflammation (Accession GSE264408). This new approach was incredibly useful for confirming and refining our definition of clusters. The corresponding figure is now Fig. 2d and the discussion of results has now been added the main text (lines 223-230).

Reviewer #3 (Remarks to the Author):

In this manuscript, Moutin and colleagues investigate the relationship between somatic mutations and clonal fate in colitis-associated tumorigenesis using a combination of in vivo lineage tracing, murine models of intestinal inflammation, spatial transcriptomics, in silico modeling, and histological analyses. This is a timely and important area of research, with potential to yield valuable insights into how chronic inflammation contributes to colorectal cancer (CRC) initiation and progression. However, the current manuscript falls short of providing the necessary mechanistic depth, conceptual clarity, and validation required for publication in Nature Genetics.

Response
We thank the reviewer for the careful evaluation of our work. We appreciate the reviewer's concerns regarding narrative cohesion, mechanistic depth, and validation. The manuscript has been substantially restructured and the analyses expanded to convey the central message:

Chronic inflammation is known to drive clonal expansion. We now document that it also generates spatially restricted cellular neighbourhoods. Those neighbourhoods engaged in tissue repair are most closely associated with tumorigenesis, whereas immune-refractory cellular neighbourhoods are enriched for clones bearing non-cancer-associated mutations.

The reviewer's specific points are addressed below.

While the study integrates several experimental platforms, it lacks a cohesive narrative and fails to generate robust or novel conclusions. Many of the findings are descriptive, speculative, or confirmatory of what is already known in the field, without offering

significant new biological insights or resolving fundamental questions about mutation selection and clonal dynamics in inflammatory contexts. For instance, while omics approaches are employed to uncover molecular features, the validation studies rely heavily on already known genes (e.g., Arid1a), rather than identifying and functionally testing novel candidates from their own dataset. The rationale for focusing on certain markers, such as TROP2 or Arid1a, is not clearly supported by the spatial transcriptomics or histological data.

Response

A. *Lack of cohesive narrative*

Substantial work has been put into improving the cohesion and narrative flow of the study, with transitions added between paragraphs, figures modified to improve readability and material and methods updated to contain additional experimental details.

B. *Descriptive and speculative findings*

We note that Nature Genetics regularly publishes foundational datasets and discovery-driven studies that provide systematic characterisation of a disease process and facilitate future research[4,5]. Our study identifies and provides a molecular characterisation of cellular neighbourhoods arising in the context of colitis and associated cancer and informs on the functional consequence of their expansion by pairing spatial transcriptomics with targeted amplicon sequencing. We believe this work aligns with Nature Genetics editorial mission by providing a unique resource and revealing biological insights that were not previously accessible.

In the revised manuscript, the upmost care has been put into removing any speculative statement and only keeping substantiated, data-driven statements (see response to specific concern #9.

C. *Lack of novelty, failure to resolve fundamental questions about mutation selection and clonal dynamics in inflammatory contexts*

Like almost any biological processes, the mechanism behind the selection of mutant clones in inflammation and associated tumorigenesis is incredibly complex and multifaceted. Our study aimed to provide insight into this fascinating problem by identifying and characterising the cellular environments favouring selection and expansion of mutant clones in a murine model of chronic inflammation.

We identified spatially discrete cellular neighbourhoods that arise in the context of colitis, and associated two distinct neighbourhoods to two different sets of mutations. We show that the repair neighbourhood is tightly correlated with tumourigenesis, while the immune-refractory neighbourhood, in which inflammation-associated mutations that are under-represented in colitis-associated cancers accumulate, appears protective against tumorigenesis by comparison.

To our knowledge, these results are entirely novel and provide an important advance in understanding the multifaceted relationship between inflammation, expansion of certain mutant clones, and inflammation-associated cancers. In the revised manuscript, our results and the discussion were further re-worked to incorporate new

sets of data from additional mouse models alongside in-depth analyses to support the overall conclusions and refined narrative.

D. Validation studies relying on known genes

We believe the reviewer refers to validation of specific markers associated with the cellular neighbourhoods identified through spatial transcriptomics using conventional methods such as RNAscope or immunostaining. However, we would argue that the power of the study was the spatial aspect of spatial transcriptomics that allowed the aggregate behaviour of transcripts to identify spatially discrete regions or neighbourhoods.

Our approach to validation employed orthogonal approaches to assign functional consequences to the neighbourhoods inferred from the transcriptional profiling. It is in this respect that Arid1a immunostaining was used to validate the loss of function associated with mutations in the *Arid1a* gene, which accumulate in the immune-refractory neighbourhood. Similarly , whilst we appreciate that other foetal genes could have been used to mark epithelial repair, Trop2 is consistently cited as associated with epithelial repair across several organs[6–8]. Trop2 has the additional advantage of clear expression at the membrane of intestinal epithelial cells that enabled identification of repairing epithelium, where other foetal genes (i.e. Anxa1, Ly6a/Sca1) are also expressed in immune cells[9] (Human Protein Atlas, Broad Institute Single Cell Portal).

A major concern is that key conclusions are not substantiated by the presented data. For example, the assertion that repair zones favor clonal expansion remains speculative and lacks experimental support. The spatial transcriptomics analysis is underdeveloped, with insufficient quality control, annotation, or validation. The predictive modeling component is also weakly justified and lacks validation, remaining anecdotal in its contribution.

Response

We have broken down our response to this paragraph in several points in order to answer each concern separately.

E. "The assertion that repair zones favor clonal expansion remains speculative and lacks experimental support"

As a point of clarification, we do not claim to have demonstrated the link between repair and clonal expansion. This association was clearly demonstrated in the Liu *et al* study published in Development Cell in 2023[10], which is now referenced lines 106-107.

We believe the reviewer is referring to the association of the repair neighbourhood with the presence of large Confetti-stained clones.
In the manuscript, clonal expansion is first visualised in discrete regions of the *Muc2*^{hom} colon tissue using Confetti lineage tracing (Fig. 1a-c) We then modelled re-epithelisation after an injury leading to a loss of epithelial cells *in silico*, to show how crypt fission and fusion events can demonstrate the clonal patterns we observe in the Muc2^{hom} mouse colon (Fig. 1d-i). Once we established this relationship between tissue repair and clonal expansion events, we sought to validate it using foetal markers, which have been associated with intestinal tissue repair by both Liu *et al* and other

studies[6,11,12]. This was done by establishing a correlation between Trop2 expression and increased fission rate (Fig. 1l). The same pattern of foetal gene expression is seen in cluster 4 (Fig. 2h), which is why this cluster is from then on referred to as the repair neighbourhood.
To clarify, the expression of foetal genes links the repair "zones" identified in Figure 1 in which clonal expansion is increased, and the repair neighbourhood later on identified through spatial transcriptomics.

F. *"The spatial transcriptomics analysis is underdeveloped, with insufficient quality control, annotation, or validation"*

We acknowledge and apologise that the previous manuscript did not go into a great amount of detail regarding the quality control pipeline applied to the spatial transcriptomic data, which essentially replicates that used by the majority of researchers and that is now standard practice. For the reviewer's benefit, we remark that we profiled about 5,000 spots per tissue piece, for which we obtained on average 100,000 reads and 3,000 genes per spot. The distribution of number of UMIs showed the expected profile for most samples: a smooth single peak with a right skewed tail. A few samples had <50% of reads mapped to the probe set, which is linked to the composition of the sample (swiss-rolled tissue leading to lower RNA content per spot). For further analysis, the number of counts and features were capped at a minimum of 500 and 200 respectively, to remove low quality spots. Additional information outlining the cutoffs in number of counts and features used has now been added to the methods section (lines 853-854).

G. *"The predictive modeling component is also weakly justified and lacks validation, remaining anecdotal in its contribution."*

Following observation of larger clone sizes in discrete regions of the chronically inflamed epithelium, the *in silico* model was implemented to explore in a quantitative manner the hypothesis that such clonal expansions are due to repair following tissue damage, using a simple yet mechanistically consistent model of crypt fission and fusion. To clarify the motivation, we have modified the introductory sentence to the relevant results paragraph (lines 121-123). In terms of empirical validation, the experimental observation that the effective fission rate of confetti labelled clones correlates with the extent of tissue repair (as measured using Trop2) (Figure 1l) provides further support for this claim.

Furthermore, the study design and figure structure suffer from poor organization. Figures 1–4 appear disconnected, and the manuscript reads as a fragmented collection of analyses rather than a focused investigation driven by a central hypothesis. The frequent shifts between different mouse models (Confetti, Muc2 KO, ENU-treated) without adequate characterization further dilute the overall message. The manuscript does not address how mutant clones are selected or maintained in inflammatory settings, nor how these dynamics contribute to CRC progression in IBD patients.

Response

We have broken down our response to this paragraph in several points to answer each concern separately.

H. *“the study design and figure structure suffer from poor organization. Figures 1–4 appear disconnected, and the manuscript reads as a fragmented collection of analyses rather than a focused investigation driven by a central hypothesis”*

As mentioned above, the manuscript has been re-structured to improve narrative flow, including figure structure and transitions between paragraphs.

I. *“The frequent shifts between different mouse models (Confetti, Muc2 KO, ENU-treated) without adequate characterization further dilute the overall message.”*

We would like to clarify that only one model (*Muc2*^{KO}) was used throughout the previous version of the manuscript. Clone dynamics is studied in this model using Confetti lineage tracing, before using the chemical mutagen ENU in the same *Muc2*^{KO} background to induce inflammation-associated tumours. Each relevant main figure panel contains a schematic summarising the experimental design, including treatments given to *Muc2*^{KO} mice.

The *Muc2*^{KO} model has been extensively characterised in previous studies[13–15], cited in the manuscript as references 12-14. In the revised version of this manuscript, to extend this characterisation and also confirm the lack of an inflammatory response in *Muc2*^{het} control mice used for lineage tracing experiments, Ab/Pas staining was performed in *Muc2*^{wt}, *Muc2*^{het} and *Muc2*^{hom} mouse colons (Supplementary Figure 1a). This experiment reveals a similar extent of mucus production in *Muc2*^{wt} compared to *Muc2*^{het} mice, and alteration of mucus production only in *Muc2*^{hom} mice. Moreover, in the revised manuscript, samples from n=3 Muc2^{het} and n=3 Muc2^{hom} mice not treated with ENU were profiled using spatial transcriptomics and annotated using clusters found in the *Muc2*^{hom}+ENU study, extending further still the characterisation of the *Muc2*^{KO} model by showing that inflammation-related neighbourhoods are associated with *Muc2*^{hom} (with or without ENU), but not Muc2^{het} mice.

Finally, we also now include, at the request of reviewers 1 and 2, an extended spatial transcriptomic characterisation of an additional model of colitis-associated-cancer (DSS+ENU), and a model of sporadic colon cancer (Apc^{het}+ENU). Those results are presented in a new separate paragraph entitled “Exploring cellular neighbourhoods in alternative tumour models” (lines 312-355) and a supplementary figure (Supplementary Figure 5), to avoid confusion with the *Muc2*^{KO} model used in the remainder of the study.

The manuscript does not address how mutant clones are selected or maintained in inflammatory settings, nor how these dynamics contribute to CRC progression in IBD patients.

Response

To reiterate our response to a previous point made by the reviewer (C above) we believe the cellular neighbourhood dynamics we describe show conclusively that the repair neighbourhood is highly correlated with tumorigenesis, while the immune-refractory lineage, in which inflammation-associated mutations that are under-represented in colitis-associated cancers accumulate, appears protective against tumorigenesis by comparison. To our knowledge, these results are entirely novel and provide an important advance in understanding the multifaceted relationship between

inflammation, expansion of certain mutant clones, and inflammation-associated cancers.

Specific concerns include:

1. The experimental and analytical design lacks clarity, and the manuscript would benefit from schematic overviews to aid reader comprehension.

Response

We hope that the extensive changes made to the text and figures throughout the manuscript improve the clarity of the analytical design. For Figure 1, presentation of key results was reorganised to align the fission neutral and fission bias scenarios, improving the reader experience. The colour code and nomenclature were also simplified (see response to the first two comments from Reviewer #4). In addition, each relevant main figure contains a schematic outlining the relevant experimental procedure. Supplementary Figure 2 outlines the overall experimental design combining spatial transcriptomic, targeted amplicon sequencing and immunostaining.

2. TROP2 analysis is unconvincing; figures selectively highlight favorable data and fail to show unbiased correlation analyses (e.g., Figure 1H–I).

Response

The Trop2 analysis is used to provide empirical validation of the association between tissue repair and increased clonal expansion, which was observed using lineage tracing in the *Muc2*^{KO} model and tested using *in silico* modelling.

We apologize for the lack of relevant methodical details in the initial submission , and the typo in the text which may have led to the assumption that the correlation analysis was based only on the immunofluorescent picture showed in Figure 1H (now Figure 1K).

The initial wording : Immunodetection revealed presence of large clones in regions where the foetal marker Trop2 (*Tacstd2*) is expressed (Fig. 1h). Estimates of effective crypt fission rates showed a significant positive correlation to extent of Trop2 expression within the region profiled (Fig. 1i).

Should have read: (…) within the regions profiled (Fig. 1i).

A description of the method used was added to the “Inference of effective fission rate from lineage tracing data” section of material and methods (lines 717-719):

“The statistical model for crypt fission described in Nicholson *et al.,* 2018 and implemented in the R package RHClones (https://github.com/ElEd2/RHClones) was adapted to infer effective fission rates using counts of clone sizes from n=3 equal size tissue areas from n=3 *Muc2*^{het} and n=3 *Muc2*^{hom} mice with different average *Trop2* expression levels, selected at random. Clone sizes were counted manually using the ImageJ cell counter tool, then the number of pixels stained with Trop2 was obtained using the Area tool in the measure function in ImageJ.”

The Trop2 analysis is framed within the wider context of foetal genes being associated with tissue repair in a separate paragraph now entitled “foetal gene expression marks repairing epithelium with elevated crypt fission rate”.

3. Bold claims regarding clonal expansion in regenerative zones are not sufficiently supported by experimental evidence.

Response
As mentioned in a previous response to the same reviewer (E above), the expression of foetal genes links the repair “zones” identified in Figure 1 in which clonal expansion is increased, and the repair neighbourhood later on identified through spatial trancriptomics.

We hope the new structure of the paragraph relating to clonal expansion in regions expressing foetal genes, as well as improved explanation of the analysis added to the methods sections (see point above) is sufficient to showcase how our experimental evidence supports the claim that clonal expansion is increased in regenerative zones.

4. The Confetti model is introduced but not meaningfully integrated into the remainder of the study.

Response
We suspect the reviewer wonders why larger expansion identified by Confetti are not used subsequently to direct analysis following ENU treatment in our screen for somatic mutations. In large part, the answer is that while Confetti works well in wildtype tissues, the maximum recombination efficiency is only around 20%. With chronic inflammation and the associated purging of crypts and clonal expansion of surviving ones there are relatively few marked clones (see Figure 3E). Events of large clones overlaying ENU induced mutations are rare and largely denote Ctnnb1 mutations (example described in the manuscript lines 377-379), which is represented in the mutation selection analysis showed in Figure 4C. Effectively this dictated our screening strategy for mutation detection of using tiled arrays of $2mm^2$ tissue cores. The core size allowed profiling of 22 genes by targeted amplicon sequencing, which would not have been possible with less material (i.e. from micro-dissected clones).

5. In silico modeling is poorly motivated and lacks empirical validation, reducing its interpretability.

Response
As stated above (point G) from the reviewer, we modified the introductory sentence to the relevant paragraph to clarify the motivation behind the *in silico* model (lines 121-123). We also added explanations in the methods regarding the analysis which showed a positive correlation between effective fission rate and expression the epithelial repair marker Trop2 (lines 717-719).

6. Spatial transcriptomic findings are largely descriptive and overlap with prior literature (e.g., immune-rich tumor margins), which are not adequately cited.

Response

With regards to overlap with prior literature, we think the reviewer is referring to the known association between inflammation/tissue repair and tumour initiation. To highlight the fact that the association between tissue repair and cancer has previously been studied in the context of colitis, a sentence was added to the discussion (lines 441-443), together with appropriate references. We believe the comment on immune-rich tumour margins is relevant to the context of sporadic colon cancer rather than colitis-associated-cancer. We have added samples from a sporadic colon cancer model to our spatial transcriptomic dataset (see reviewer 1 point #1), which showed differences in cellular populations found at tumour margins compared to our model of colitis-associated-cancer. Namely, a cellular neighbourhood expressing markers of cancer-associated-fibroblasts was identified in close proximity to tumours in the sporadic colon cancer model, which was not seen in the context of colitis-associated-cancer.

Additionally, we highlight that, in the revised manuscript, we have performed extensive quantitative analyses of the data using state-of-the-art algorithms, including trajectory inference with SpaceFlow and SlingShot, spatial proximity enrichment with Giotto, and DWLS deconvolution (Figure 3). Together, these quantitative analyses substantiate the refined narrative, which identifies and characterises the phenotypic and genotypic nature of colitis-associated cellular neighbourhoods and the associations between them. We believe these results substantiate the novel conclusion that the two, major tissue repair and immune-refractory neighbourhoods contain distinct somatic mutations, which are positively and negatively associated with carcinogenesis, respectively.

7. Annotation of the “inflammation-resistant” cluster (Figure 4) lacks clear rationale or supporting evidence.

Response

We note that the nomenclature for this cluster has been updated to “immune refractory.” Deconvolution of cell types from the spatial transcriptomics data using DWLS demonstrated that this cluster is the most immune-enriched among epithelial-rich clusters. However, despite the presence of immune cells, this cluster shows significant negative enrichment for immune-responsive pathways in the new GSVA analysis requested by reviewer #2 (point 2), now provided in Supplementary Table 2, with the example of TGF-B signalling showed in Figure 2G.
In particular, the coordinated downregulation of multiple immune-associated programs (TGF-β signalling, interferon-α response, apoptosis, peroxisome activity, heme metabolism, and reactive oxygen species pathways) indicates that this cluster remains transcriptionally unresponsive to immune cues despite immune infiltration, supporting its designation as immune-refractory.

8. Use of three distinct mouse models is not well justified and potentially confounds interpretation regarding IBD-related tumorigenesis.

Response

As mentioned in response to this reviewer concern (I above), VillinCreERT2; Muc2KO; Rosa26_Confetti mice were used throughout the study, first to study clonal fates in the context of colitis, and then with the addition of ENU to profile cellular neighbourhoods

arising in the context of colitis-associated-cancer and mutations associated with such neighbourhoods.

As prompted by other reviewers, we included another model of chemical colitis and associated cancer, as well as a model of sporadic colon cancer. We hope the nomenclature used and separation of the additional mouse models into a separate result paragraph aids readers to interpret our findings regarding IBD-related tumourigenesis.

9. Several speculative statements throughout (e.g., lines 250–252, 263) are not backed by data.

Response
In the revised manuscript, the utmost care has been taken to remove any speculative statements.

For the specific statements mentioned here:

Previous lines 250–252 have been removed: "Neutrophil binding to ICAM-1 on epithelial cells was shown to trigger proliferation via Akt and β-catenin signalling[16]. In this context, it could enhance wound healing but could also drive Wnt-dependant neoplasia."

We now discuss the role that neutrophils could have in driving tumourigenesis in the discussion section (lines 441-443). Of note, this claim is now additionally supported by a new analysis quantifying the spatial proximity between the neutrophil-rich cluster 11 and tissue repair cluster 4, both associated with the tumour cluster (Figure 3b and 3e).

Previous lines 263: "Overall, this analysis revealed the dynamics of cellular neighbourhoods in the progression of disease, with response to inflammatory cues dictating association with neoplasia."

This statement was removed and replaced with a set of analyses addressing the dynamic relationships between different cellular neighbourhoods (Figure 3, see also response to Reviewer #2, point 4).

10. No meaningful differential gene expression or pathway analysis is presented between genotypes in the Muc2 model.

Response
We think the reviewer refers to differential gene expression between *Muc2^{hom}* and control *Muc2^{het}* mice. Such comparative studies have previously been published, including an account of transcriptomic and proteomic differences between both genotypes[13–15].

Regarding the new data obtained within this study and pertaining to differential gene expression between *Muc2^{hom}* and *Muc2^{het}* mice, we have made the bulk transcriptomic dataset obtained by comparing n=3 *Muc2^{het}* and n=3 *Muc2^{hom}* mouse colons available to download on the GEO website (accession number GSE297394).

Additionally, colon tissue pieces were collected from n=3 *Muc2^{het}* and n=3 *Muc2^{hom}* mice and profiled by spatial transcriptomics, data which are now included in the revised manuscript (Figure 2i) and accessible to download through the Figshare link associated with the manuscript.

11. The statement that Arid1a IHC "demonstrates" the expansion of clones in inflammation-resistant regions is overstated.

Response
This statement has been replaced by a refined interpretation of the corresponding results: "These results support an interpretation where clones carrying *Arid1a* inactivating mutations are selected for in environments where responses to inflammatory cues are dampened" (lines 413-415).

12. Final conclusions (lines 378–380) are vague and unsupported.

Response
The paragraph highlighted by this reviewer has been modified to end with the conclusion cited in response to the preceding point by the same reviewer. We have also included a statement on possible out-competition of neoplastic clones by mutations selected in immune refractory neighbourhoods, an interpretation that we believe is suitably supported by our data and underlies the conclusions of related studies in other tissue types[17] (lines 413-416).

13. Central questions: how inflammation selects mutant clones, and how this impacts tumorigenesis, remain unaddressed.

As outlined in a previous response to the reviewer, the mechanism behind the selection of mutant clones in the context of chronic inflammation and associated tumorigenesis is complex and cannot be fully deciphered in one study. We provided insight into the cellular environments favouring selection and expansion of mutant clones in a murine model for inflammation. Our finding that progression along the repair lineage is highly correlated with increased tumour burden, whilst progression to an immune-refractory state is comparatively protective against tumorigenesis, is entirely novel.

A complete mechanistic understanding of the phenomenon remains the focus of potentially decades of additional work by multiple research groups working in this fascinating field. To emphasise this, we finish the discussion by including some concrete implications of our study (lines 454-460).

Minor issues:
• The manuscript is difficult to follow, with poor flow between sections and abrupt transitions. Removal of subheadings (e.g., line 175) may improve readability.

Response
As mentioned in several points above, the overall cohesion and narrative flow of the study was revised. Transitions between results sections were amended to considerably improve the overall flow between them.

• Several figure panels lack clarity, annotations, and appropriate controls.

Response
We hope the restructuring of figures and legends increases clarity. In addition, *Muc2^{het}* and *Muc2^{hom}* (no ENU) controls were included in the spatial transcriptomic analysis (see response to Reviewer #2, point 2).

Reviewer #4 (Remarks to the Author):
The manuscript "Paired mutation calling and spatial transcriptomics identify cellular neighbourhoods dictating the neoplastic outcome of colitis" assesses the mutational and transcriptional landscape of tissue progressing from IBD tissue to IBD-associated cancer. The work and investigative/analytical approach adopted in this manuscript are both interesting and very worthy of publication. Whilst many of the key findings are not entirely novel (e.g. inflammation promotes expansion of pro-tumorigenic clones, the association between inflammation duration and clonal expansion), the study provides robust evidence supporting the clonal dynamics underlying the previously established molecular pathogenesis of colitis-associated colorectal cancer (CRC). This contributes valuable confirmation and clarity to the existing body of knowledge.

Overall manuscript comments
Overall, the manuscript is well-written and easy to follow. However, the final sections discussing the analysis of the mutational landscape are somewhat more complex and could benefit from simplification to improve readability and comprehension.

Response
We thank the reviewer for their positive and constructive comments on our work.

Specific comments
Figure 1a, 1b and 1c / Lines 104-112
Could the author explain the reason behind their choice to use Muc2het mice as controls instead of Muc2Wt. Do Muc2het mice get any inflammation? If yes, would a Muc2wt mouse be a better baseline control? Moreover, could the comparison between Muc2het and Muc2hom used to infer on the relationship between inflammation extent and clonal expansion (as we already know that in humans more severe inflammation is associated with great CRC risk)?

Response
We hope the following answers this question appropriately. For added clarity on this matter, in the revised manuscript a panel was added to Supplementary Figure 1 illustrating mucin production in *Muc2^{wt}*, *Muc2^{het}* and *Muc2^{hom}* small bowel and colon, showing reduction in mucin production only in the *Muc2^{hom}* colon. *Muc2^{wt}* and *Muc2^{het}* small bowels and colons are alike, and in accordance we never saw any clinical signs of inflammation in *Muc2^{het}* mice, and their survival is similar to *Muc2^{wt}* mice[15]. *Muc2^{het}* mice can be bred and maintained in the same cages as *Muc2^{hom}* mice in an efficient breeding strategy, which explains our decisions to use them as controls.

Figure 1e and 1f:
The labelling and colouring used in Figures 1E and 1F is slightly confusing. My understanding is that the grey colour represents the undamaged grid, which also

corresponds to the grid under homeostatic conditions. In Figure 1F (left), the grey line appears to represent both the undamaged grid from Figure 1E and the grid prior to the application of damage. If this is correct, then in Figure 1F (right), the grey line should correspond to the undamaged/homeostatic condition, and the red line should represent the damaged/regenerating condition. Could you please clarify if this interpretation is accurate?

• Could the authors consider making the figure legends for Figures 1E and 1F slightly more detailed to better guide the reader through the data presentation? A similar suggestion applies to Figures 1K and 1L, where additional explanation would help improve clarity and aid interpretation.

Response

We apologise for the confusion regarding the interpretation of our *in silico* simulations, which was caused in part by our previous presentation of the corresponding figure panels. In the revised Fig. 1 d-h, we have reorganised the illustration of key results by presenting both fission neutral (upper) and fission bias (lower) scenarios in direct alignment to help guide the reader's eye. We have additionally simplified the colour code to enable a more intuitive mapping between the quantities of interest. In panels Fig. 1 d,g we now show simulation snapshots with undamaged (left) and damaged (right) sides of the grid bordered in grey and red, respectively. Next, in panels Fig. 1 e,h we now plot the total number of empty (black), occupied labelled (green) and occupied unlabelled (blue) sites averaged across 1000 simulations, the important point here being that the undamaged side (left, grey border) attains a homeostatic state to which the damaged side (right, red border) returns to post-repair. Finally, in panels Fig. 1f,i we now plot the aggregation of sites for both undamaged (grey line) and damaged (red line) on the same axis to display the elevated aggregation in the damaged region following repair. The corresponding text and figure legend has been modified to accommodate these improved figures, and we hope the interpretation of key results is now much more apparent to the reviewer and general reader.

• If my understanding of Figure 1F (right) is correct, the persistence of the damaged grid/line is expected and illustrates that, following an initial increase in site aggregation, this begins to decline over time. If the simulation was extended further, would the level of aggregation eventually plateau at the same level as the undamaged grid, or would it stabilise at a new, elevated homeostatic state? (hinting at a form of long-term memory following damage)

Response

We thank the reviewer for this remark and raising the fascinating question of whether our simulations can address the role of clone aggregation as a form of long-term tissue memory. To investigate the point raised regarding the asymptotic behaviour of the aggregation metric, we ran simulations for many more simulation time steps (1 million, up from 200,000) and provide a zoomed-in comparison of site aggregation at late time on both the left- (undamaged) and right-hand (damaged) sides of the grid-- see Figure below. At this level of resolution, it appears that the level of aggregation does indeed plateau at a higher value on the right-hand grid, potentially suggesting some lasting form of memory following damage. However, we are quick to highlight that this observation could be impacted by imperfect averaging over a finite number (1000) of simulations which, while sufficient for capturing the larger trends over shorter timeframes, may be insufficient for capturing the subtle, asymptotic features of

aggregation. Analysis of the asymptotic properties of our model instead requires a rigorous mathematical treatment of the stochastic process, which will be the focus of future work.

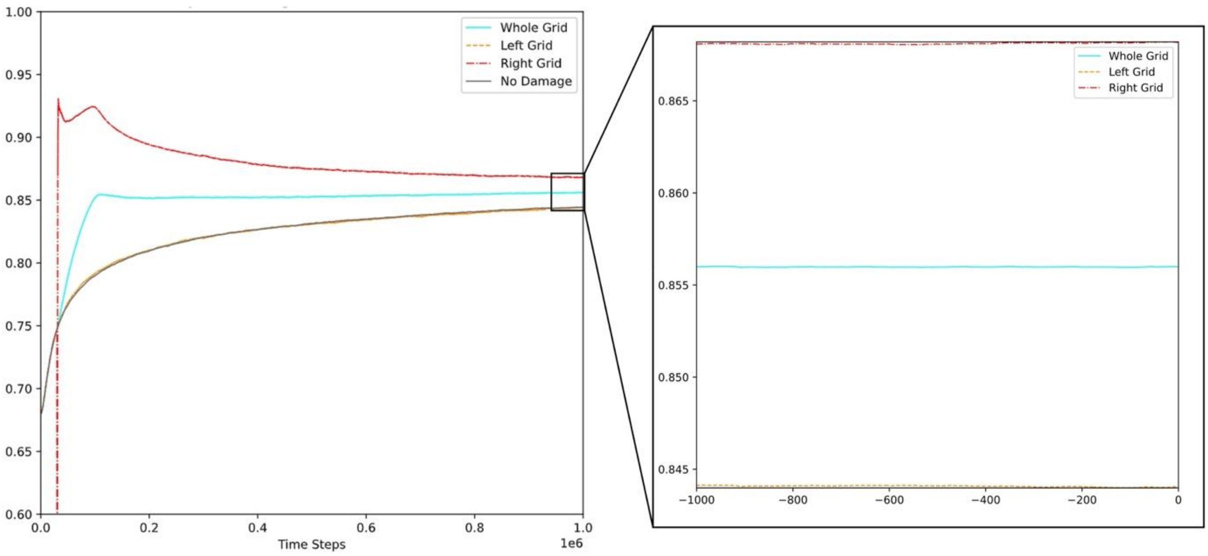

• Typo in line 131

Response
The sentence was rephrased and typo corrected.

Supplementary Figure 1:
• Statistical analysis appears to be missing from the survival plot.

Response
P values for log-rank test were added to the Kaplan-Meiri survival plot in Supp. Fig. 1D.

• What does the Muc2het small bowel look like?

Response
The *Muc2*^{het} small bowel does not show any sign of inflammation, just as the *Muc2*^{hom} small bowel. To illustrate this, we performed Alcian Blue/Pas staining of mucins in *Muc2*^{wt}, *Muc2*^{het} and *Muc2*^{hom} mice small bowel and colon. This shows a reduction in the production of mucins in the *Muc2*^{hom} colon specifically, whilst *Muc2*^{wt} and *Muc2*^{het} small bowel and colon are alike (Supplementary Figure 1a)

Lines 139-144:

• While the findings are certainly interesting, the conclusions regarding increased crypt fission are not particularly novel, as increased crypt fission has been previously reported in the context of both regeneration and IBD. Nonetheless, the authors present elegant proof of concept experiments that reinforce and illustrate these observations effectively.

Response

Thank you for these positive comments.

Lines 154-157:
• The authors state “This highlights how tissue repair conditions can accelerate and promote the accumulation and expansion of mutant clones in damaged tissue.” in relation to their in-silico observation of fission-biased clones. Would it be more correct to state that the data highlights how tissue repair conditions can accelerate and promote the accumulation and expansion of clones with a proliferative (or fission biased) advantage rather mutant clones in general.

Response
We thank the reviewer for spotting this generalisation. Text lines 154-157 were updated to: “This highlights how tissue repair conditions can accelerate and promote the accumulation and expansion of fission-biased clones in damaged tissue.”

Lines 169-172
• Should these lines be merged with the next paragraph?. Identification of cellular neighbourhoods in colitis-associated neoplasia/biological processes defining epithelial neighbourhoods

Response
Text lines 169-172 were included into the “identification of cellular neighbourhoods in colitis-associated neoplasia” paragraph.

• The authors should be commended for their elegant experimental design, robust analytical approach, and their ability to extract key insights into clonal dynamics and cellular neighbourhoods from spatial transcriptomics data.

Response
We thank the reviewer for this very positive comment.

Line 205
• Typo in neighbourhood

Response
The typo was corrected. Text line 232: “Biological processes defining epithelial neighbourhoods”

Lines 188-200
• The authors report: high, low, abundant… expression of the different keratins. How is high/low being defined? Is there a numerical/statical value that is being used as cut off. As for example, visually cluster 11 also appears to be enriched for keratin 7 and keratin 14

Response
Bar plots showing the percentage of cells expressing each of the keratins presented in each cluster were added to Supp. Fig3e. This shows a high expression of Krt7 and 14 in cluster 11 (neutrophil-rich cluster) as the reviewer highlighted. This is in line with the spatial correlation we observed between cluster 11 and the tumour cluster 10 and

adds to the evidence that neutrophils infiltrating epithelial neighbourhoods may be participating in the promotion of a tumorigenic environment.

Figure 2g / Lines 237-242:
• The association between regenerative epithelium and tumorigenic tissue is not a novel finding, especially in the context of IBD-CRC, as previously reported by Gil Vazquez et al., 2022. Nevertheless, the current study provides valuable proof of this relationship at the cellular level.

Response
Thank you for pointing this out. We added a sentence to the discussion, mentioning the specific reference suggested by the reviewer[18], as well as Levi-Galibov et al., 2020[19]: "Tissue repair and the associated remodelling of the extracellular matrix is commonly viewed as cancer promoting47,48." (Lines 435-436).

Figure 3 / Lines 268-289
• The mutational landscape detected in the Muc2hom + ENU mice has some clear differences from that reported in human IBD-CRC (e.g. Apc highly mutated in the mice, but reported to be mutated only in 10% of IBD-CRC, p53 highly mutated in IBD-CRC (>70%) but lowly mutated in the mice). Can the authors comment on this. While the overall message with regards to association of specific mutational signatures with specific clusters remains intact, from a mutational point of view is this perhaps not the best model of IBD-CRC? Does the Muc2hom + ENU model at the genetic level represent more of a model of sporadic CRC with an inflammatory push.

Response
The reviewer is correct, tumorigenesis in our model is Wnt-driven, which is not commonly the case for IBD-related colon cancer. Unfortunately, this is also the case for the more established AOM+DSS model[20]. Importantly, using the *Muc2*^{hom}+ENU model we were able to detect some mutations present in human IBD-related colon cancer, such as *Smad4* mutations, as well as mutations present in human IBD such as *Arid1a*, *Nfkbiz* and *Il17rc*, giving translational value to our findings regarding accumulation of oncogenic or non-oncogenic mutations in distinct cellular neighbourhoods.

• Having said that the findings on Arid1a are compelling and do fit well with the high mutational burden of the gene in human IBD-CRC.

Response
Thank you for this positive comment.

Supplementary Figure 6
• In terms of the tumour associated genes and their distribution: are different Wnt mutations were mutually exclusive?

Response
The co-occurrence of tumour-associated *Ctnnb1* and *Apc* mutations was assessed across cores. Most cores did not carry either mutation (154/185 cores). Among mutated cores, 20% carried both mutations, at face value giving the impression that *Ctnnb1* and *Apc* mutations are largely mutually exclusive. However, Fisher's exact test

showed no statistically significant association between the two mutations ($p = 0.31$), indicating that the observed co-occurrence could arise by chance.

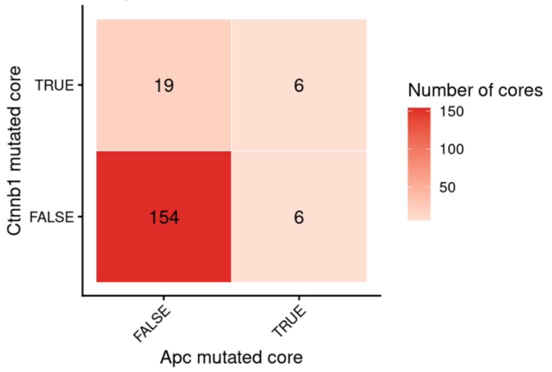

• Can the association between specific mutations and specific cluster be used to infer a temporal acquisition of the mutations?

Response

We thank the reviewer for this interesting suggestion. We looked at the distribution of *Ctnnb1* mutations identified as tumour-associated in tumour vs non-tumour cores. T41I, S37P, I35S and D32G were found in both tumour and non-tumour cores, suggesting they may be acquired earlier than the other *Ctnnb1* mutations profiled.

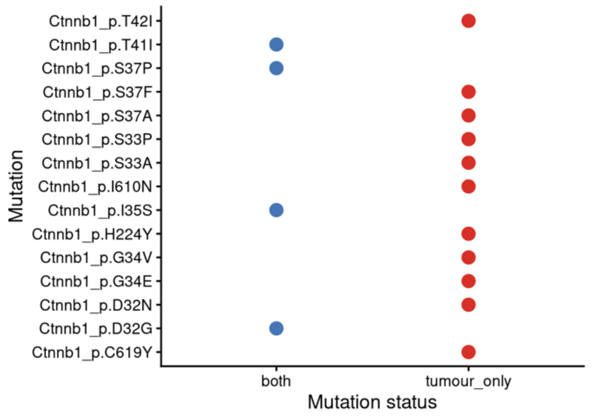

Minor Comment:

The introduction is well written and provides a solid background for the manuscript. However, it would benefit from referencing key publications that have previously examined the mutational landscape of colitis-associated colorectal cancer, such as Baker AM, Cross W, Curtius K, et al. Gut, 2019 Including these studies would help contextualise the current work within the existing literature.

Response

Lines 78-81: The introduction was re-worked to include seminal work that characterised the mutational landscape of colitis associated cancer: "Extensive characterisation of the mutational landscape of CAC has revealed a reduced prevalence of inflammation-resistance mutations in tumours arising in the context of IBD[20–24], hinting at a protective role against cancer."

Reviewer #4 (Remarks on figshare data availability):

The data looks robust and agreement with the claims in the paper

Response
Thank you for this positive comment.

References

1. Coussens, L. M. & Werb, Z. Inflammation and cancer. *Environ Health Prev Med* 23, (2018).
2. Putoczki, T. L. *et al.* Interleukin-11 is the dominant IL-6 family cytokine during gastrointestinal tumorigenesis and can be targeted therapeutically. *Cancer Cell* 24, 257–271 (2013).
3. Švec, J. *et al.* TROP2 Represents a Negative Prognostic Factor in Colorectal Adenocarcinoma and Its Expression Is Associated with Features of Epithelial–Mesenchymal Transition and Invasiveness. *Cancers (Basel)* 14, (2022).
4. Oliveira, M. F. de *et al.* High-definition spatial transcriptomic profiling of immune cell populations in colorectal cancer. *Nature Genetics 2025 57:6* 57, 1512–1523 (2025).
5. Yost, K. E. *et al.* Three-dimensional genome landscape of primary human cancers. *Nature Genetics 2025 57:5* 57, 1189–1200 (2025).
6. Yui, S. *et al.* YAP/TAZ-Dependent Reprogramming of Colonic Epithelium Links ECM Remodeling to Tissue Regeneration. *Cell Stem Cell* 22, 35-49.e7 (2018).
7. Fernandez Vallone, V. *et al.* Trop2 marks transient gastric fetal epithelium and adult regenerating cells after epithelial damage. *Development (Cambridge)* 143, 1452–1463 (2016).
8. Liu, Q. *et al.* Increased expression of TROP2 in airway basal cells potentially contributes to airway remodeling in chronic obstructive pulmonary disease. *Respir Res* 17, 159- (2016).
9. Yui, S. *et al.* YAP/TAZ-Dependent Reprogramming of Colonic Epithelium Links ECM Remodeling to Tissue Regeneration. *Cell Stem Cell* 22, 35-49.e7 (2018).
10. Liu, C. Y. *et al.* Wound-healing plasticity enables clonal expansion of founder progenitor cells in colitis Wound-healing plasticity enables clonal expansion of founder progenitor cells in colitis. *Dev Cell* 1–17 (2023) doi:10.1016/j.devcel.2023.08.011.
11. Nusse, Y. M. *et al.* Parasitic helminths induce fetal-like reversion in the intestinal stem cell niche. *Nature* 559, 109–113 (2018).
12. Ayyaz, A. *et al.* Single-cell transcriptomes of the regenerating intestine reveal a revival stem cell. *Nature* https://doi.org/10.1038/s41586-019-1154-y (2019) doi:10.1038/s41586-019-1154-y.
13. Van der Sluis, M. *et al.* Muc2-Deficient Mice Spontaneously Develop Colitis, Indicating That MUC2 Is Critical for Colonic Protection. *Gastroenterology* 131, 117–129 (2006).
14. Wenzel, U. A. *et al.* Spontaneous colitis in Muc2-deficient mice reflects clinical and cellular features of active ulcerative colitis. *PLoS One* 9, 1–12 (2014).
15. Moutin, E. B. *et al.* Extracellular Matrix Orchestration of Tissue Remodeling in the Chronically Inflamed Mouse Colon. *CMGH* 17, 639–656 (2024).
16. Sumagin, R. *et al.* Neutrophil interactions with epithelial-expressed ICAM-1 enhances intestinal mucosal wound healing. *Mucosal Immunol* 9, 1151–1162 (2016).
17. Colom, B. *et al.* Spatial competition shapes the dynamic mutational landscape of normal esophageal epithelium. *Nat Genet* 52, 1–11 (2020).

18. Gil Vazquez, E. *et al.* Dynamic and adaptive cancer stem cell population admixture in colorectal neoplasia. *Cell Stem Cell* 29, 1612 (2022).
19. Levi-Galibov, O. *et al.* Heat Shock Factor 1-dependent extracellular matrix remodeling mediates the transition from chronic intestinal inflammation to colon cancer. *Nat Commun* 11, 1–19 (2020).
20. Chatila, W. K. *et al.* Integrated clinical and genomic analysis identifies driver events and molecular evolution of colitis-associated cancers. *Nat Commun* 14, 1–13 (2023).
21. Baker, A. M. *et al.* Evolutionary history of human colitis-associated colorectal cancer. *Gut* 68, 985–995 (2019).
22. Yin, J. *et al. P53 Point Mutations in Dysplastic and Cancerous Ulcerative Colitis Lesions*. *GASTROENTEROLOGY* vol. 104 (1993).
23. Susan Galandiuk, Manuel Rodriguez-Justoll, Rosemary Jeffery, Anna M. Nicholson, Yong Cheng, Dahmane Oukrif, George Elia, Simon J. Leedham, Stuart A. C. Mcdonald, Nicholas A. Wright, and T. A. G. Field cancerization in the intestinal epithelium of patients with Crohn's ileocolitis. *Gastroenterology* 142, 855–864 (2012).
24. Brentnall, T. A. *et al.* Mutations in the p53 gene: An early marker of neoplastic progression in ulcerative colitis. *Gastroenterology* 107, 369–378 (1994).

Reviewer #3 (Remarks to the Author):

The manuscript has improved substantially in its revised form. Nevertheless, significant work remains before the study can meet the standards required for publication in Nature Genetics. At present, the manuscript does not fully support the stated aim of "providing a unique resource and revealing biological insights that were not previously accessible." Much of the analysis appears confirmatory rather than conceptually or technically advancing the field. While validation of known biology is appropriate, the framing continues to overstate the level of novelty relative to what is demonstrated. My primary concern remains the identification and interpretation of cellular neighborhoods. The spatial transcriptomics (ST) analysis, as currently presented, is not sufficiently rigorous to justify the strength of the claims. Several conclusions extend beyond what the resolution and analytical framework can robustly support.

Response

We thank the reviewer for acknowledging that our previous revisions lead to a substantial improvement to the manuscript. We hope the additional analysis and improvements to the manuscript outlined in this response and current revised version of the manuscript adequately address to the reviewer's outstanding comments.

We believe these add even further support the main conclusions of the paper, which are that: 1) chronic colitis leads to the expansion of cellular neighbourhoods with distinct phenotypic/cell type compositions (tissue repair and immune refractory, respectively); 2) the immune refractory neighbourhood is favourably enriched for mutations (e.g. Arid1a) found to undergo positive selection in the colon of IBD/UC patients but under-represented in colitis-associated cancers (Kakiuchi et al., 2020; Nanki et al., 2020; Olafsson et al., 2020); 3) the repair neighbourhood is tightly associated with tumorigenesis while the immune refractory neighbourhood is mutually exclusive by comparison.

Below, I outline specific issues in the order they appear in the manuscript:

Line 197 – Spatially contiguous neighborhoods.
The statement that "most clusters formed spatially contiguous, cellular neighbourhoods" does not appear novel. With barcoded spot–based spatial transcriptomics, spatial contiguity is largely expected due to tissue architecture and local mixing of cell types. It is unclear what additional biological insight this observation provides. If the authors intend to convey more than a technical consequence of the method, this should be clearly articulated and supported.

Response

Thank you for pointing this out. Indeed, our intention here was simply to convey a technological consequence of the method used, which leads to the definition of cellular neighbourhoods referenced throughout the remainder of the text. Lines 200-202 have been re-worded to reflect this and clarify that we are not attempting to provide any quantitative insight into the spatial distribution of cell types among spots at this stage in the manuscript:

"Mapping barcoded spots within each cluster to their spatial coordinates revealed the organisation of cellular neighbourhoods in each tissue sample."

Differentially expressed genes (DEGs).

DEGs are not shown in the main figures and are reportedly provided only in Table 1, which is not accessible. Throughout the manuscript, similarities and differences between clusters are described without clear visual or quantitative support. This limits interpretability and makes it difficult to assess the robustness of the cluster annotations.

Response

Thank you for raising these points. We previously uploaded all supplementary tables, including DEGs and GSVA between clusters, in csv format so they could be accessible during review. However, it appears that csv files are automatically converted to PDF format during compilation of the manuscript files, which may mean they are not fully interpretable by the reviewers.

To complement the reporting of top pathways identified for each cluster using GSVA (Fig. 2G) and provide further visual and quantitative support to the differences between clusters, we have now added a dot plot showing the top 2 marker genes per cluster to support cluster annotations (Supplementary Fig. 3e). This replaces annotations of top markers that used to be in Supplementary Fig. 3b.

Selection of top markers is outlined in methods (lines 870-874). Briefly, we filtered the list of genes differentially expressed in each cluster compared to other clusters to keep genes expressed in at least 50% of barcoded spots in the cluster (pct.1>0.5) and expressed in less than 50% of barcoded spots in other clusters (pct.2<0.5). This filtered list was ordered by descending log2 fold change and ascending adjusted p value, and the top 2 markers per clusters are now shown in Supplementary Fig. 3e. Furthermore, selected marker genes from Supplementary Figure 3 are referred to in context while discussing the biological nature of and assigning cellular neighbourhoods to clusters (lines 205-230).

scRNA-seq reference dataset.

The reference dataset consists of only three healthy mouse colon samples. This is a limited basis for annotation and may not capture the full diversity of relevant cell states. As a result, important populations may be absent, directly affecting annotation accuracy.

Response

We appreciate the concern of the reviewer. To address this in this revised version of the manuscript, we have repeated cell type deconvolution using cell signatures based on a reference scRNA-seq now containing ten colon samples (Hong *et al., Com. Biology* 2024; accession: GSE264408) including: n=3 healthy (as before), n=3 acute DSS colitis and n=4 chronic DSS colitis (see methods lines 889-891). It is worth highlighting that, while the major cell types used for annotation with this much larger reference data set remain the same, inclusion of acute and chronic samples now allows for greater annotation accuracy of cell populations (e.g. epithelial populations located in inflamed tissue regions) as the reviewer suggests. Fig. 2e was updated to reflect the results of this more extensive analysis and we include a comparison of the old and new annotations below, for the benefit of the reviewer. Overall, the general distribution of cell types remains highly similar, albeit with a tendency for slight enrichment in the proportion of fibroblast cells in all clusters. We thank the reviewer for motivating this extended analysis, which has helped to strengthen the annotation of clusters.

Original reference dataset (n=3 healthy colon)

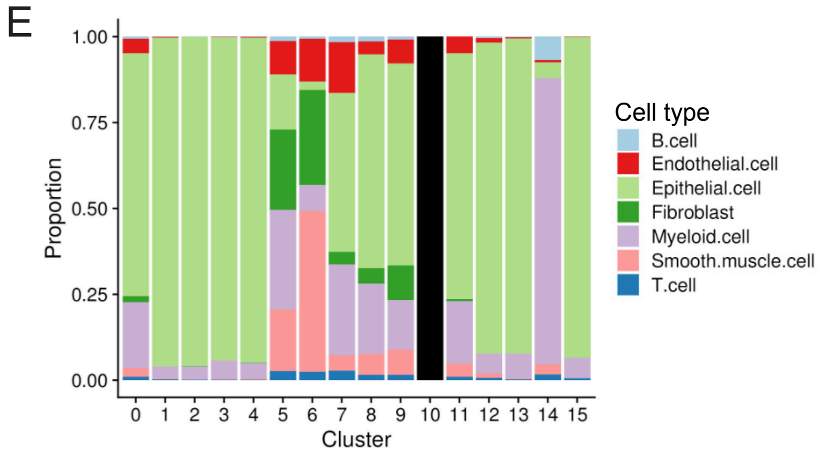

New reference dataset (n=3 healthy colon, n=3 acute DSS colitis, n=4 chronic DSS colitis)

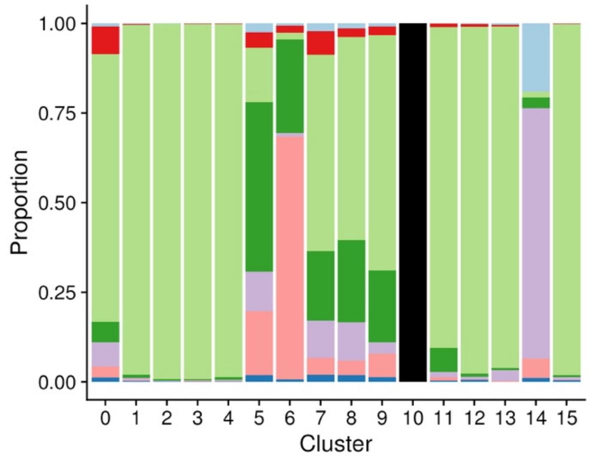

In Figure 2E, cell type proportions are presented based on gene expression, yet the underlying marker genes are not clearly shown. Cluster 10 does not convincingly correspond to a defined cell type. It is unclear whether tumor annotation is based solely on β-catenin together with KRT7 and KRT20 expression (Line 219). This is insufficient to justify definitive tumor classification without additional supporting evidence.

Response

Further information on using marker genes to annotate cellular neighbourhood has now been included in the revised manuscript (see lines 205-230, Supplementary Fig. 3e and response to reviewer's previous point above). We would like to clarify that unambiguous assignment of cluster 10 as the tumour cluster was not based on spatial transcriptomics results, but on *independent* pathological assessment of these regions using haematoxylin and eosin staining, and further validated using β-catenin immunostaining, a combination of staining used in previous studies (Sadien *et al., Nature* 2024). In the revised version of the figures, an example of such H&E staining was added to Fig. 2c to make this clear. Lines 223-226 were also modified to mirror this. The colour scheme excluding the tumour cluster (cluster 10) from Fig. 2e is based on these experimental data combined with an understanding that the scRNA-seq reference used for cell type deconvolution does not contain representatives of tumour cell types, and results would therefore be spurious if applied to this cluster.

Distribution across sections.

The manuscript states that the first clusters cover most of the tissue; however, 16 sections were analyzed. The distribution of clusters across sections is not described. It remains unclear whether specific clusters are enriched in tumor regions, follicles, or other

structures. Without spatial stratification across sections, the biological interpretation of the clustering remains incomplete.

Response

To make the distribution of clusters across sections more transparent, a bar plot showing distribution of barcoded spots mapped to each cluster over each of the 16 sections has been added as Supplementary Fig. 4b, which is referenced in text lines 201 and 244.

Figure 2G – Heatmap legend.

The meaning of the grey color in the heatmap is not defined and should be clarified.

Response

We thank the reviewer for pointing out this discrepancy. The figure legend was amended to include the meaning of the grey colour in the heatmap in Fig. 2g: the specific pathway was not identified in the enrichment analysis for the relevant cluster.

Figure 2I – Cluster proportions and ordering.

Cluster 1 appears to have similar proportions in Muc2^het and Muc2^hom samples, yet the text (Line 258) states that it is increased. This discrepancy requires correction.

Response

As the reviewer points out, the proportion of cluster 1 in $Muc2^{het}$ and $Muc2^{hom}$ samples is approximately the same. To improve the readability of this paragraph, we added a full stop between the sentence which points out that epithelial clusters 1, 2 and 3 make up the largest proportion of barcodes in $Muc2^{het}$ samples, and the sentence stating that the proportion of barcoded spots mapping to the anti-inflammatory cluster 0 and tissue repair cluster 4 is increased in $Muc2^{hom}$ compared to $Muc2^{het}$ samples (lines 268-273).

In addition, cluster ordering throughout the manuscript is inconsistent and difficult to follow. While numerical ordering (0, 1, 11, 12, etc.) may reflect bioinformatic conventions, it is not reader-friendly. Reordering clusters across figures further reduces clarity.

Response

To improve figure readability and the defining features of individual cellular neighbourhoods throughout the revised manuscript, the nature (once established in Fig. 2) of epithelial clusters 0, 4 and 10 has been added as text accompanying the cluster numbers in all relevant

figures. Furthermore, as suggested by the reviewer, clusters in Fig. 3c were reordered numerically to match the presentation in other figures.

Cluster annotation clarity.

Clusters are compared without consistent annotation, making interpretation unnecessarily challenging. For example, in Line 254 the authors refer to an “intermediate phenotype” between clusters 0 and 4, yet these clusters are not clearly defined at that point in the text.

Response

Although the relationship of each cluster to the tumour cluster has not been described by line 254, the differential gene expression and GSVA revealed marker genes and functional features associated with each cluster. The relevant sentence was updated to remove mentions of intermediate phenotype which may be confusing.

Lines 263-265:

“By comparison, pathways active in the remaining epithelial-rich clusters 1, 2 and 3 indicate normal intestinal epithelium function (fatty acid metabolism) and some enrichment for interferon gamma response as seen in the tissue repair cluster 4.”

They are described as epithelial-enriched, but clusters 12, 13, and 15 are not adequately contextualized in Figure 2E.

Response

We agree with the reviewer that additional characterisation of these smaller clusters would help improve the manuscript. We found that clusters 12 and 13 express markers of specialised intestinal cell populations (goblet and enteroendocrine respectively). Their scattered presence is likely due to tissue orientation, which varies for each slide for which Visium was applied.

Cluster 13 is marked by the B cell marker *Iglc1*, which together with cell type deconvolution results identify this cluster as epithelium enriched in B cells.

This characterisation of small clusters 12, 13 and 15 has been added to the manuscript on lines 205-208 and 211-214.

A systematic and consistent labeling strategy is required throughout.

Response

As detailed above in response to a previous point on this topic, annotations for the main epithelial clusters 0, 4 and 10 have been added throughout the relevant figures. Annotations are limited to these clusters to limit clutter in figures.

Arid1a.

Arid1a continues to appear isolated within the analysis and is not clearly integrated into the broader biological narrative.

Arid1a deletion is consistently identified across independent studies as positively selected in the human IBD epithelium (Kakiuchi et al., 2020, Nanki et al., 2020, Olafsson et al., 2020), and its inactivation was linked to immune exclusion in the normal colonic epithelium (Skoufou-Papoutsaki et al., 2025). However, since this relevance of Arid1a mutations was previously confined to the last result section, we agree it could look isolated from the broader, biological narrative. To mitigate this, a sentence referring to Arid1a was added to the revised introduction lines 78-82:

The mechanisms underlying this resistance are diverse: mutations in the IL17 and NF-KB pathways act directly by dampening inflammatory signalling, whilst others such as Arid1a inactivation act through chromatin remodelling to alter the transcriptional response to inflammatory cues[5].

We thank the reviewer for motivating this improvement to the manuscript.

Overall, the spatial transcriptomics analysis remains the most concerning component of the study. The conclusions drawn from these data are stronger than what the analysis convincingly supports. The manuscript would benefit from more rigorous validation, clearer and consistent annotation, improved visualization of supporting data—particularly DEGs—and a more restrained interpretation of novelty.

Response

We would like to thank the reviewer for their input, which we feel has helped us significantly improved the presentation of our work. We hope they agree that these improvements reinforce the validity and impact of the conclusions of our study.
